# Processing of RNA Containing 8-Oxo-7,8-Dihydroguanosine (8-oxoG) by the Exoribonuclease Xrn-1

**DOI:** 10.3389/fmolb.2021.780315

**Published:** 2021-11-15

**Authors:** Cheyenne N. Phillips, Shawn Schowe, Conner J. Langeberg, Namoos Siddique, Erich G. Chapman, Marino J. E. Resendiz

**Affiliations:** ^1^ Department of Chemistry, University of Colorado Denver, Denver, CO, United States; ^2^ Department of Chemistry, University of Denver, Denver, CO, United States

**Keywords:** RNA, XRN-1, oxidized RNA, Xrn-1 stalling, 8-oxoG-RNA MALDI, RNA degradation

## Abstract

Understanding how oxidatively damaged RNA is handled intracellularly is of relevance due to the link between oxidized RNA and the progression/development of some diseases as well as aging. Among the ribonucleases responsible for the decay of modified (chemically or naturally) RNA is the exonuclease Xrn-1, a processive enzyme that catalyzes the hydrolysis of 5′-phosphorylated RNA in a 5′→3′ direction. We set out to explore the reactivity of this exonuclease towards oligonucleotides (ONs, 20-nt to 30-nt long) of RNA containing 8-oxo-7,8-dihydroguanosine (8-oxoG), obtained *via* solid-phase synthesis. The results show that Xrn-1 stalled at sites containing 8-oxoG, evidenced by the presence of a slower moving band (*via* electrophoretic analyses) than that observed for the canonical analogue. The observed fragment(s) were characterized *via* PAGE and MALDI-TOF to confirm that the oligonucleotide fragment(s) contained a 5′-phosphorylated 8-oxoG. Furthermore, the yields for this stalling varied from app. 5–30% with 8-oxoG located at different positions and in different sequences. To gain a better understanding of the decreased nuclease efficiency, we probed: 1) H-bonding and spatial constraints; 2) anti-syn conformational changes; 3) concentration of divalent cation; and 4) secondary structure. This was carried out by introducing methylated or brominated purines (m^1^G, m^6,6^A, or 8-BrG), probing varying [Mg^2+^], and using circular dichroism (CD) to explore the formation of structured RNA. It was determined that spatial constraints imposed by conformational changes around the glycosidic bond may be partially responsible for stalling, however, the results do not fully explain some of the observed higher stalling yields. We hypothesize that altered π-π stacking along with induced H-bonding interactions between 8-oxoG and residues within the binding site may also play a role in the decreased Xrn-1 efficiency. Overall, these observations suggest that other factors, yet to be discovered/established, are likely to contribute to the decay of oxidized RNA. In addition, Xrn-1 degraded RNA containing m^1^G, and stalled mildly at sites where it encountered m^6,6^A, or 8-BrG, which is of particular interest given that the former two are naturally occurring modifications.

## Introduction

Oxidative stress occurs from an imbalance between the generation of reactive oxygen species (ROS) and the biological mechanisms naturally designed to cope with the same. These highly reactive intermediates can be produced endogenously and exogenously (Küpfer et al., 2014) and their interactions with RNA have attracted attention recently due to their relationship to disease, e.g., neurodegeneration, (Nunomura et al., 2017) cardiac hypertrophy, (Seok et al., 2020) bipolar I disorder, (Jacoby et al., 2016) and diabetes (Cejvanovic et al., 2018). Among the characterized outcomes of oxidative stress resulting from ROS with RNA includes strand cleavage, (Zinskie et al., 2018) cell death, (Liu et al., 2012) activation of signaling pathways, (Bazin et al., 2011; Gonzalez-Rivera et al., 2020) altered protein biosynthesis, (Willi et al., 2018) and nucleobase damage that leads to the same lesions that have been observed/characterized in DNA (Cadet and Wagner, 2013). Among the canonical nucleobases, guanine is the most prone to reactions with some ROS, due to its lower oxidation potential (Steenken and Jovanovic, 1997). One of the main outcomes from such reactions leads to the generation of 8-oxo-7,8-dihydroguanosine (8-oxoG), a common biomarker for oxidative stress (Christensen et al., 2018; Rodríguez-Callejas et al., 2019). These aspects bring into question the factors, mechanisms, and biological pathways that have evolved to handle oxidative damage of RNA. Among these, ribonucleases play a major role in the processing and decay of RNA for its maturation or disposal, therefore it is important to understand how these enzymes accommodate modified substrates (Li et al., 2014; Yan and Zaher, 2019). In a recent report, the exoribonuclease Xrn-1 was shown to be directly involved in degrading mRNA damaged by oxidation, specifically RNA containing 8-oxoG (Yan et al., 2019). Another study showed that Xrn-1 (along with Rat1) play an important role in the degradation of mature, hypomodified, tRNA, (Chernyakov et al., 2008) thus highlighting its ability to process and degrade RNA containing various modifications. Furthermore, this exoribonuclease plays an important role in the host immune defense, degrading viral RNA which has been shown to generate subgenomic flavivirus RNA (sfRNA), (Silva et al., 2010) or Xrn-1 resistant RNAs (xrRNAs) in hosts infected with flaviviruses (Akiyama et al., 2021). Xrn-1 has also been suggested as a potential druggable target, due to its involvement in limiting double stranded RNA accumulation in infected/uninfected cells with Vaccinia virus (VacV) (Burgess and Mohr, 2015). We also considered reports from our group that describe differences in the way oxidatively damaged RNA reacts with RNases, as well as structural differences that arise from the presence of these type of lesions (Herbert et al., 2018). This prompted us to explore the degradation of oxidatively damaged RNA by Xrn-1, using oligonucleotides of RNA containing 8-oxoG as models.

Xrn ribonucleases were first described over four decades ago (Stevens, 1980). Xrn-1, the prototypical member of this class of enzymes, is a 5′→3′-exoribonuclease that plays key roles in mRNA processing and turnover, no-go decay (NGD), nonsense mediated decay (NMD), and the RNAi pathway. During 5′→3′ mRNA degradation in eukaryotes, messages are irreversibly targeted for decay by deadenylation which is then followed by removal of the 5′-m^7^G cap by the decapping complex and subsequent 5′→3′ hydrolysis by Xrn-1 (Sheth and Parker, 2003; Gatfield and Izaurralde, 2004; Jones et al., 2012; Boehm et al., 2016; Mugridge et al., 2018; Chang et al., 2019). This exoribonuclease employs a two metal ion mechanism to cleave the substrate RNA yielding 5′-NMP products. A crystal structure of *Drosophila melanogaster* Xrn-1 shows how RNA recognition is achieved, (Jinek et al., 2011) through binding of the 5′-monophosphate-trinucleotide-end *via* a continuous π-π stacking complex between conserved histidine and tryptophan residues.

In this study we found that the presence of 8-oxoG within RNA presents a challenge for Xrn-1 and stalls its processive degradation in ratios that varied on the position of the oxidative lesion within the oligonucleotide, as well as its sequence. To rationalize this observation the following factors were taken into consideration, and approached as follows: 1) to probe the impact of the anti→syn conformational change arising from the functionalization at the C8-position of purine rings, oligonucleotides containing 8-bromoguanosine (8-BrG) were prepared, where this chemical modification adopts the syn-conformation; 2) the efficiency of Xrn-1 in the presence of nucleobases with altered H-bonding patterns was explored *via* the incorporation of methyl groups using 1-methylguanosine (m^1^G) and *N,N*-6-dimethyladenosine (m^6,6^A) as models, where the presence of the methyl groups inhibit the expected canonical Watson-Crick (WC) base pairing interactions, and may serve as probes for spatial variations within the active site; 3) the potential impact of divalent cations, in this case Mg^2+^; and 4) structural changes imposed by 8-oxoG, assessed *via* circular dichroism (CD). It was found that rotation around the glycosidic bond, as well as spatial interactions may be partially responsible for the observed stalling, and we hypothesize that altered π-π stacking interactions or adverse H-bonding may also play a major role in this regard.

## Experimental—Methods

The phosphoramidites of 8-oxoG and 8-BrG were obtained using reported synthetic routes (Choi et al., 2017). The procedures leading to the *t*-butyldimethylsilyl (TBDMS)-protected phosphoramidites of the alkylated modifications, m^1^G and m^6,6^A, as well as the uridine-bisphosphate derivative, are included within the supporting materials (pp. S3-S26, Supplementary Figures S1–S46). All reagents and enzymes were obtained from commercial sources and used without further purification, with the exception of Xrn-1, *vide infra*. Electrophoretic analyses were monitored by tracking the migration of a marker containing bromophenol blue and xylene cyanol.

**RNA synthesis (oligonucleotides 1–19).** Oligonucleotides were synthesized using a 394 ABI DNA/RNA synthesizer using CPG supports (1 μmol) and 2′-O-TBDMS phosphoramidites (Glen Research). 0.25M 5-ethylthio-1H-tetrazole in acetonitrile was used as the coupling reagent; 3% trichloroacetic acid in dichloromethane was used for deblocking; a 2,6-lutidine/acetic anhydride solution was used for capping; and an iodine solution (0.02 M in/THF/pyridine/water) was used in the oxidation step (Glen Research). Coupling times of 10 min were used. Oligonucleotides (ONs) were deacetylated/debenzoylated/deformylated and cleaved from the solid (CPG, 1 μmol loading) support in the presence of 1:1 aq. methylamine (40%) and aq. ammonia (40%) with heat (60°C, 90 min). A mixture of *N*-methylpyrrolidinone/triethylamine/HF (3:2:1) was used for deprotection of the TBDMS groups by heating to 60°C for 90 min followed by purification *via* denaturing acrylamide gel electrophoresis (20% denaturing dPAGE). C18-Sep-Pak cartridges (Waters) were used to desalt the purified oligomers using 5 mM NH_4_OAc as the elution buffer. Oligonucleotides were concentrated under reduced pressure, dissolved in H_2_O and used for subsequent experiments. Typical yields ranged from 45–200 nmol of RNA. All sequences used in this work are displayed in Table 1.

**TABLE 1:** RNA sequences used in this work. **1**-**8** represent one sequence context, while **9**–**16** and **17**–**19** correspond to a different sequence context, canonical and modified.

#	Sequence (RNA)
1	5′- CAU GAA ACA AGG CUA AAA GU
2	5′- CAU GAA ACA A(8-oxoG)G CUA AAA GU
3	5′- CAU GAA ACA A(8-oxoG)(8-oxoG) CUA AAA GU
4	5'- (8-oxoG)G CUA AAA GU
5	5'- (8-oxoG) CUA AAA GU
6	5′- CAU GAA ACA (m6,6A)GG CUA AAA GU
7	5′- CAU GAA ACA A(m1G)G CUA AAA GU
8	5′- CAU GAA ACA A(8-BrG)G CUA AAA GU
9	5′- GAC GAA ACA GGG CUA AAG AU
10	5′- GAC GAA ACA (8-oxoG)GG CUA AAG AU
11	5′- GAC (8-oxoG)AA ACA GGG CUA AAG AU
12	5′- GAC GAA ACA (8-oxoG)(8-oxoG)G CUA AAG AU
13	5′- GAC GAA ACA (8-oxoG)(8-oxoG)(8-oxoG) CUA AAG AU
14	5′- GAC GAA ACA (m1G)GG CUA AAG AU
15	5′- GAC GAA ACA (m1G)(m1G)G CUA AAG AU
16	5′- GAC GAA (m6,6A)CA GGG CUA AAG AU
17	5′-UGU CAA CUC CAG GAC CAC CUA CAC ACC UC
18	5′-UGU CAA CUC CA(8-oxoG) GAC CAC CUA CAC ACC UC
19	5′-UGU CAA CUC CA(8-oxoG) (8-oxoG)AC CAC CUA CAC ACC UC

**UV-vis spectroscopy.** The concentration of all oligonucleotide solutions was assessed *via* UV-vis using a 1 mm path-length with 1 μL volumes (Thermo Scientific Nano Drop Nd-1000 UV-vis spectrometer). Origin 9.1 was used to plot the spectra of monomers and oligonucleotides for comparison.

**RNA characterization (MALDI-TOF).** All oligonucleotides were characterized *via* mass spectrometry (MALDI-TOF MS) using equilibrated C18 Zip Tip pipette tips as follows: 1) wash tip with 50% acetonitrile (10 μL × 2); 2) equilibrate tip with 0.1% TFA (10 μL × 2); 3) load tip with sample (typically 100–200 picomol); 4) wash tip with 0.1% TFA (10 μL × 2); 5) wash tip with water (10 μL × 2); 6) elute sample into matrix (10 uL of 25 mM-2,4,6-trihydroxyacetophenone monohydrate, 10 mM ammonium citrate, 300 mM ammonium fluoride in 50% acetonitrile); 7) spot directly onto MALDI plate. The desalted eluate (1 μ) was spotted on the MALDI target and allowed to air dry. The calibrant (1 μL) was spotted, allowed to dry and then overlaid with the described matrix (1 μL). Molecular weight measurements were performed on a Microflex-TOF mass spectrometer (Bruker Daltonics, Billerica, MA) in positive ion, linear mode using an ion source voltage of 20 kV and laser frequency of 20 Hz. Scan ranges were 300–7,500 m/z and 1,500–7,500 m/z. External calibration was performed using a protein calibration mixture (Bacterial Test Standard, Bruker Daltonics) on a spot adjacent to the sample. The raw data was then processed in the FlexAnalysis software (version 3.4, Bruker Daltonics). See acknowledgements and supporting information, all spectra are available in Supplementary Figures S47–S63 (pp. S28-S33).

**Xrn-1 fragment characterization.** RNA **2** or **3** (200 pmol) was diluted to yield a buffered solution (42 μL, 70 mM Tris-HCl, 10 mM MgCl_2_, 5 mM DTT, pH 7.6), ATP (5 μL, 10 mM), polynucleotide kinase enzyme (3 µL from New England BioLabs Inc.). The resulting solution was incubated for 45 min (37°C) and then heated for 10 min (65°C). A 10X buffered solution (5 μL, 1 M NaCl, 500 mM Tris-HCl, 100 mM MgCl_2_, 10 mM DTT, pH 7.9) was added, followed by addition of exonuclease Xrn-1 (1–5 μL, New England Biolabs) and incubation at rt (1 h). The resulting solution was then passed through a Nanosep 10 K Ω filter (Pall) with centrifugation (15,000 × g, 10 min). The filter was then washed with H_2_O (20 µL) by centrifugation (15,000 × g, 10 min). Sample preparation for MALDI analysis was carried out as described in the previous step (Supplementary Figures S64, S65).

**Circular dichroism spectroscopy and thermal denaturation transitions (T**_**m**_ **).** CD spectra were recorded at various temperatures (PTC-348W1 peltier thermostat) using Quartz cuvettes with a 1 cm path length. Spectra were averaged over two scans (350–200 nm, 3 nm bandwidth, 4 s data integration time) and background corrected with the appropriate buffer or solvent. Solutions containing the RNA had the following composition: 4 μM RNA, 5 mM MgCl_2_, 100 mM NaCl, 10 mM sodium phosphate-pH 7.2. Thermal denaturation transitions (T_m_) were measured by hybridization of the oligonucleotides of interest by heating to 90 C followed by slow cooling to room temperature. T_m_ values were recorded at 270 nm with a ramp of 1.2°C/min and step size of 0.2 with temperature ranges from 4–95 C. A thin layer of mineral oil was added on top of each solution to keep concentrations constant at higher temperatures. Origin 9.1 was used to determine all T_m_ values and to plot all CD spectra. All spectra and T_m_ measurements were carried out in duplicate and are included within the supporting materials (Supplementary Figures S66–S78, pp. S35-S39). T_m_ analyses *via* CD has been previously validated and leads to results that are comparable to those typically obtained using UV-vis spectroscopy.^(31)^

**SCHEME 1:**
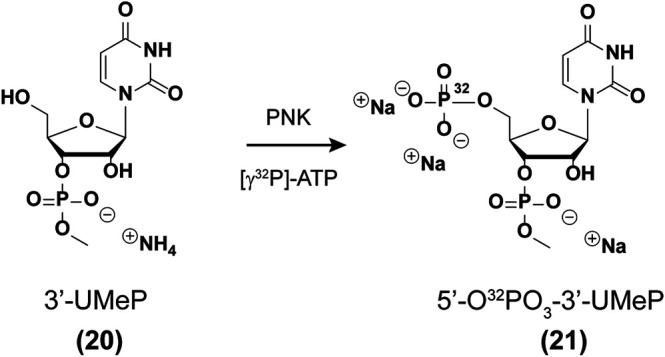
5′-Radiolabeling of phosphate 20.

**5′-Radiolabeling of uridine-3′-methylphosphate (20 → 21).** Prepare a cocktail solution as follows: H_2_O (34 µL), uridine-3′-methylphosphate (**20**, 5 μL, 1.3 mM), ATP [*γ*-^32^P] (4 μL, 10 mCi/ml, 3.3 µM), 10X buffer (4 μL, 700 mM Tris-HCl, 100 mM MgCl_2_, 50 mM DTT, pH 7.6), Polynucleotide Kinase enzyme (3 μL, New England Biolabs Inc.). The solution was incubated at 37 °C for 45 min and filtered using a Nanosep 10 K Ω filter (Pall) with centrifugation (15,000 × g, 10 min). The filter was washed with H_2_O (15 µL) by centrifugation (15,000 × g, 10 min) and both aliquots were combined. A 100% conversion was assumed to yield solutions of uridine bisphosphate **21** (app. 100 µM, Scheme 1), although more studies are underway to confirm the exact efficiency of this transformation.

**3′-Radiolabeling of oligonucleotides of RNA using uridine-methylphosphate (21).** A cocktail solution was prepared as follows: H_2_O (5.5 µL), ligase buffer (3 μL, 400 mM Tris-HCl, 100 mM MgCl_2_, 100 mM DTT, 5 mM ATP, pH 7.8), BSA (3 μL, 50 mg/ml in 20 mM sodium phosphate, pH 6.8), ATP (1 μL, 10 mM), uridine-methylphosphate **21** (12 μL, 100 µM), RNA (5 μL, 10 µM), T4 RNA ligase (1.4 µL, 10 U/µL, ThermoFisher Scientific). The sample was incubated at rt (12 h) and desalted using a Sephadex G25 column, washed with H_2_O (31 µL) prior to use. Loading buffer (40 μL, 90% formamide) was added and the sample was then purified *via* 20% dPAGE. The band of interest (slowest) was excised and eluted over a buffered solution (400 μL, 100 mM sodium phosphate, pH 7.4) at 37°C (12–15 h). The solution was filtered using a polyethyelene column (30 μm, Bio-Rad) and centrifugation (3,400 rpm, 5 min). The sample was then concentrated to dryness and dissolved in H_2_O (55 µL). A concentrated solution of sodium acetate was added (8 μL, 3 M, pH 5.6), followed by ethanol (200 µL). The solution was cooled (15 min, dry ice—ethanol mixture, app. −40°C) and centrifuged while keeping a cool temperature (15 min, 4–10°C, 12,000 x g). The supernatant was removed and the remaining pellet was dried under reduced pressure, followed by addition of H_2_O (50–80 µL). The radiolabeling efficiency was measured using a scintillation counter, prior to use in experiments. A typical experiment yielded 5,000–15,000 cpm/µL in 80 µL H_2_O in our hands.

**Xrn-1.** The protein was expressed as described previously, (Langeberg et al., 2020) and diluted to yield a final concentration of 1.8 ng/ml (Table 2). Alternatively, the exoribonuclease was also obtained from a commercial source (New England BioLabs Inc.).

**TABLE 2:** Exonuclease concentration and storage buffer for Xrn-1 used in this work.

Entry	Xrn-1	Source	Storage buffer
1	1.8 ng/ml–10.3 nM	In-house	50 mM Tris pH 7.5, 250 mM NaCl, 2 mM DTT, 50% glycerol
2	120 ng/ml–0.686 µM	NEB	20 mM Tris-HCl, 500 mM NaCl, 2 mM DTT, 0.1 M EDTA, 50% glycerol, 0.1% Triton^®^ X-100

**Xrn-1 Degradation assays.** A typical degradation assay was carried out by preparing a cocktail solution containing H_2_O (18.5 µL), 10X Buffer solution (2.5 µL: 700 mM Tris-HCl, 100 mM MgCl_2_, 50 mM DTT, pH 7.6), ATP (1.5 µL, 100 mM), polynucleotide kinase enzyme (1.5 µL from New England BioLabs Inc.), 3′-radiolabeled RNA (1 μL, 10,000 cpm/µL, app. 0.6 µM). The solution was then incubated at 37°C (45 min) followed by increased heat (65°C, 10 min) and subsequent cooling to rt. A 10X buffered solution (2.5 µL, 1 M NaCl, 500 mM Tris-HCl, 100 mM MgCl_2_, 10 mM DTT, pH 7.9) was added and used for the degradation assays. The cocktail solution (5 µL) was mixed with diluted Xrn-1 or water (2 µL), followed by incubation at room temperature for 1–3 h. A loading buffer solution (6.5 µL, 90% formamide) was then added and an aliquot of the corresponding mixture was loaded onto a 20% dPAGE (43 cm × 35 cm). Gels were electrophoresed until the xylene cyanol dye reached 1/3 the length of the gel, followed by exposure using an autoradiography cassette (Amersham Biosciences) overnight. Quantification of radiolabeled oligonucleotides was carried out using a Molecular Dynamics Phosphorimager 840 equipped with ImageQuant Version 5.1 software.

**Magnesium dependent Xrn-1 degradation assays.** 5′-Phosphorylation was carried out as in the previous step. A representative cocktail solution was prepared by mixing H_2_O (46 µL), 10x Buffer solution (7.5 µL: 700 mM Tris-HCl, 100 mM MgCl_2_, 50 mM DTT, pH 7.6), ATP (5 μL, 100 mM), polynucleotide kinase enzyme (3.5 µL, New England BioLabs Inc.), 3′-radiolabeled RNA (13 μL, 6,000 cpm/µL, app. 0.6 µM). The solution was then incubated at 37°C (45 min) followed by increased heat (65°C, 10 min) and subsequent cooling to rt. The reaction solution was desalted using a Sephadex G25 column, rinsed with H_2_O (75 µL) prior to use. The solution was then divided into tubes containing the desalted RNA (7.5 µL), a NaCl solution (1 μL, 1M), and a MgCl_2_ solution (1.5 µL–50 mM, 10 mM, 5 mM, 1 mM, 500 μM, 100 μM, or 50 µM). The resulting solution (5 µL) was transferred into a separate tube, followed by addition of an Xrn-1 solution (2 µL), and incubation at rt for 3 h. A loading buffer solution (6.5 µL, 90% formamide) was then added and an aliquot of the corresponding mixture was loaded onto a 20% dPAGE (43 cm × 35 cm). Gels were electrophoresed until the xylene cyanol dye reached 1/3 the length of the gel, followed by exposure using an autoradiography cassette (Amersham Biosciences) overnight. Quantification of radiolabeled oligonucleotides was carried out using a Molecular Dynamics Phosphorimager 840 equipped with ImageQuant Version 5.1 software.

## Results

In order to carry out the 3′-end labeling, we set out to synthesize a nucleotide analog of the previously reported PCP [cytidine 3,5,-bis(phosphate)] derivative (Pellegrini et al., 2008). Uridine-3′-methylphosphate (**20**, Scheme 1) was chosen over the other canonical nucleotides given that it lacks an exocyclic amine, thus not needing additional protecting/deprotecting steps in the synthetic plan. Furthermore, a methyl group was installed at the 3′-phosphate to maintain anionic character at (-1) and better mimic the substrate for a ligation reaction. The synthesis for the uridine derivative (**20**) was adjusted from a previous report (Korhonen et al., 2012) (see SI for full experimental procedures and details pp S18-S26) and is fully characterized herein. The enzymatic phosphorylation was carried out assuming full conversion to the corresponding bis(phosphate) derivative (**21**). Ligation of each RNA with nucleotide (**21**) was carried out using T4 RNA ligase (see experimental section), to yield 3′-end radiolabeled oligonucleotides (Nilsen, 2014).

**FIGURE 1:**
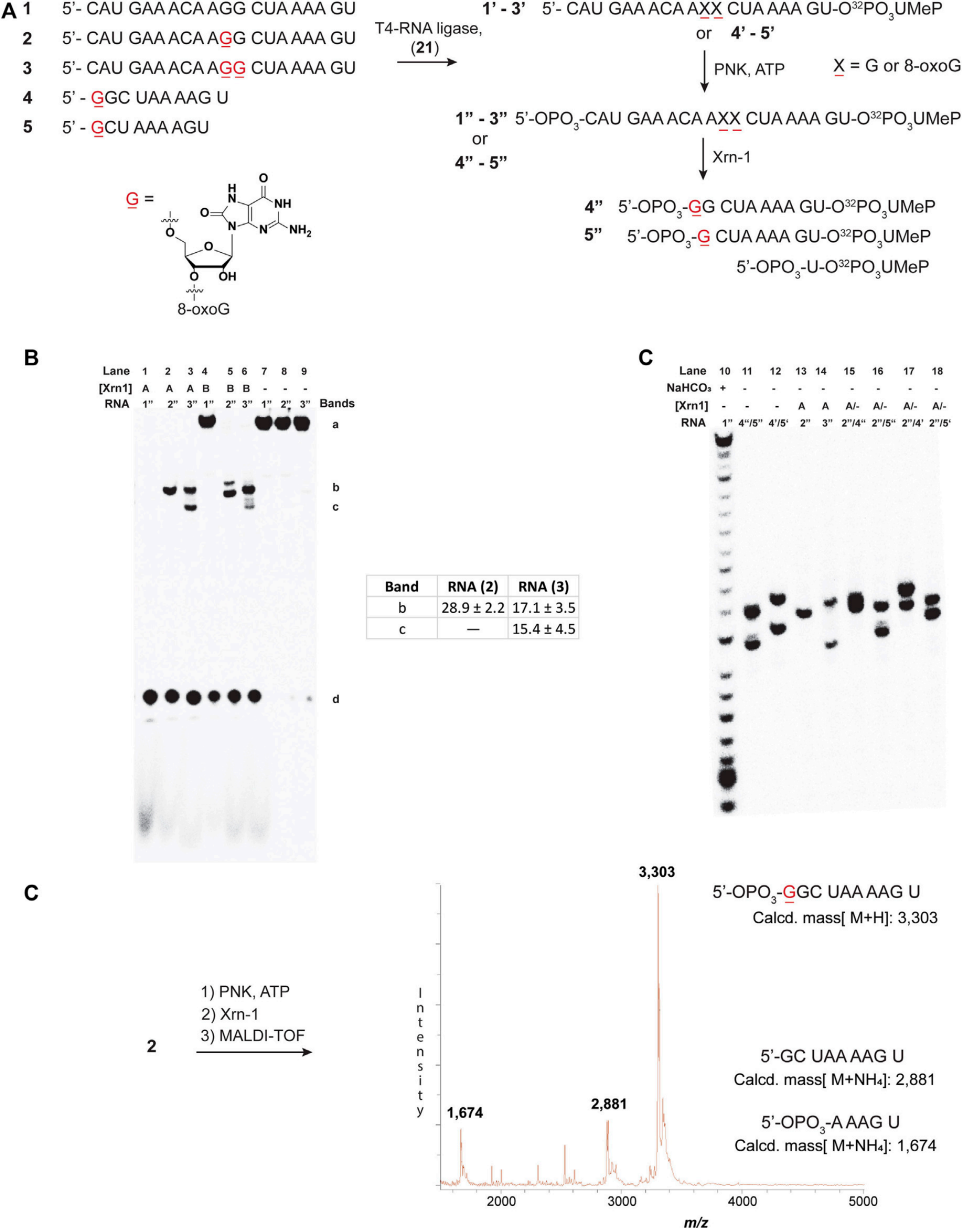
Sequences **1**-**5** and the overall process for 3′-radiolabeling (using nucleotide transformation **20**→**21**), and 5′-phosphorylation of the corresponding oligonucleotides **(A)**; and 20% denaturing PAGE of oligonucleotides 1″-3″ in the presence of Xrn-1 in the absence **(B)** and presence of markers **4** and **5 (C)**. The difference between lanes 1-3 and 4-6 in **(B)** represents a 10-fold dilution in [Xrn-1], respectively, where A = 0.12 pg, B = 0.012 pg, or (−) no enzyme added, per experiment. Reactions were carried out in buffered solutions (100 mM NaCl, 50 mM Tris▪HCl, 10 mM MgCl_2_, 1 mM DTT, pH 7.9) by incubating for 2 h (rt). Further characterization of the fragment of interest was obtained *via* MALDI-TOF, which matched the mass of ON **4** phosphorylated at the 5′-end **4 (D)**.

To explore the reactivity of Xrn-1 with oxidatively damaged RNA, we chose the sequence of a 20-nt fragment (ON **1**) from the 3′-UTR of serotype 2 Dengue Virus (DENV2) where this single stranded portion (position 10,286–10,267) has been shown to be a substrate for degradation by Xrn-1, up to the point where the RNA becomes structured (not included in the chosen construct) (Chapman et al., 2014). Additionally, this sequence contains a section where two consecutive G’s are present, thus making it an attractive model for G-site substitutions with 8-oxoG at one or two positions (Figure 1A). Oligonucleotides **1**-**3** were prepared *via* standard solid-phase synthesis, then radiolabeled, and phosphorylated prior to reactions in the presence of Xrn-1, as depicted in Figure 1A (**1–3 → 1**″-**3**″). We validated that temperature did not play a role in experiments carried out at 37°C or at room temperature, thus all experiments were carried out at rt. The lack of temperature dependent results is consistent with previous reports, where similar data was obtained in temperature ranges between 0°C and 30°C (Stevens, 1978). In agreement with the known reactivity of Xrn-1, the canonical ON (**1**″) was degraded efficiently and showed a single band that was consistent with a short fragment, presumably a dinucleotide (Figures 1B, lanes 1 and 7). Interestingly, we found that the presence of one 8-oxoG lesion (ON **2**″) led to stalling of the ribonuclease, evident by the appearance of a new band that migrated slower than the dinucleotide and with an intensity corresponding to app. 30% yield (Figures 1B, lane 2). Furthermore, the corresponding RNA strand containing two lesions (ON **3**″) also led to stalling and displayed the appearance of two bands (Figures 1B, lane 3), which suggested that stalling was induced by 8-oxoG. To gain more insight into these observations, the enzyme concentration was decreased 10-fold, which displayed the same stalling bands with the exception that in the case for the strand containing two 8-oxoG lesions degradation stopped at the first site, suggesting that the processivity of the enzyme is impacted upon the appearance of the first oxidative lesion. To corroborate the exact location of stalling, and identify the nature of the new band(s), two markers (10-nt and 9-nt long) containing 8-oxoG at the 5′-ends (**4**, **5**) were synthesized and loaded alongside/together with samples treated with Xrn-1 (Figure 1C). These experiments confirmed that the enzyme stalled upon encountering the oxidative lesion at a fragment corresponding to the 5′-phosphorylated ON **4**″. The stalling fragment moves at the same speed as fragment **4**″ (Figure 1C, lanes 11 and 13), which was confirmed by loading both samples on the same well to see co-migration, and the appearance of a single band (lane 15). This was not the case with bands corresponding to **4′, 5**″ or **5′** (lanes 16–18), where two bands were clearly observed in all cases. MALDI-TOF was used to further characterize the fragment of interest, allowing for unequivocal assignment of the stalling position, and confirming the identity of stalling fragment **4** phosphorylated at the 3′-end (Figure 1D, major peak). Other minor fragments were also observed, which can be rationalized by partial sample degradation, presumably, arising from handling and preparation (full spectrum is included in Supplementary Figure S64).

To corroborate this result, the same approach was carried out on ON **3**, followed by MALDI-TOF analysis. This led to the appearance of two peaks with masses corresponding to a 5′-phosphorylated analog of ON **4** (with one additional 8-oxoG), as well as the corresponding 5′-phosphorylated 9-mer **5** (Supplementary Figure S65).

It is important to note that the 3′-labeling efficiencies, along with varying degrees of degradation in the process, results in (RNA) variations (Supplementary Figure S79). This, in turn, leads to varying ribonuclease efficiency, while keeping the enzyme concentration constant between experiments. An example is highlighted in Figure 1B (lanes 4, 5, and 6), where the canonical ON was not fully degraded, while the modified ONS display full degradation. As a consequence, care must be taken when making conclusions about exoribonuclease reactivity.

We also considered exoribonuclease efficiency as a function of time, however, no significant changes were observed, and the band(s) corresponding to stalling were always present. Supplementary Figure S80 shows an example where experiments carried out as a function of time (0–15 h) displayed the band(s) corresponding to stalling, (**2**″ → **4**″ + 5′-NMPs), where the overall % conversion was constant at app. 29.6 ± 1%. Typical % conversions were calculated by comparing both degradation bands [% stalling = 100 (band c/band c + band d), Figure 1B]. Furthermore, analyses of the same experiment using ON **3**″ also displayed the stalling bands at all times in a conversion values app. of 40.6 ± 0.9% [% stalling = 100 (b + c/b + c + d), Figure 1B]. These values are consistent with those obtained following full degradation (Figure 1B, table inset), and suggest that the stalling occurs in a time independent manner. Additionally, experiments where the enzyme concentration varied, resulted in values that were consistent with these ranges (Supplementary Figure S81).

Another variable that was considered, regarded the concentration of the divalent metal (in this case magnesium), which has been previously shown to be necessary for efficient degradation, with concentrations at app.1.5 mM leading to optimal exoribonuclease activity (Stevens, 1978). The active site of the enzyme has been shown to contain magnesium or manganese in the presence/absence of an oligonucleotide substrate (Chang et al., 2011; Jinek et al., 2011). To establish the concentration of magnesium, considering the presence of ATP (known to chelate to Mg) in the cocktails, (Achuthan et al., 2014) samples were passed through a desalting column, a process that also eliminates the presence of nucleotides. Reactions were carried out in Mg^2+^ concentrations that varied in the 10 mM–7.5 µM range. All reactions fully degraded the RNA, and monitoring the amounts of the band assigned to stalling showed that [Mg^2+^] between 10–1.5 mM had the maximum overall yield for this band. Reactions with a concentration of magnesium between 750–75 µM displayed stalling bands with app. 10% diminished intensity (Supplementary Figure S82), and experiments carried out at 15 µM [Mg^2+^] or below resulted in inhibited enzyme activity. Although not associated with the stalling band, a faster moving band, presumably corresponding to 5′-UMeP (**5**) appeared as a function of decreasing divalent cation, reaching a maximum intensity of app. 30–40%.

**FIGURE 2:**
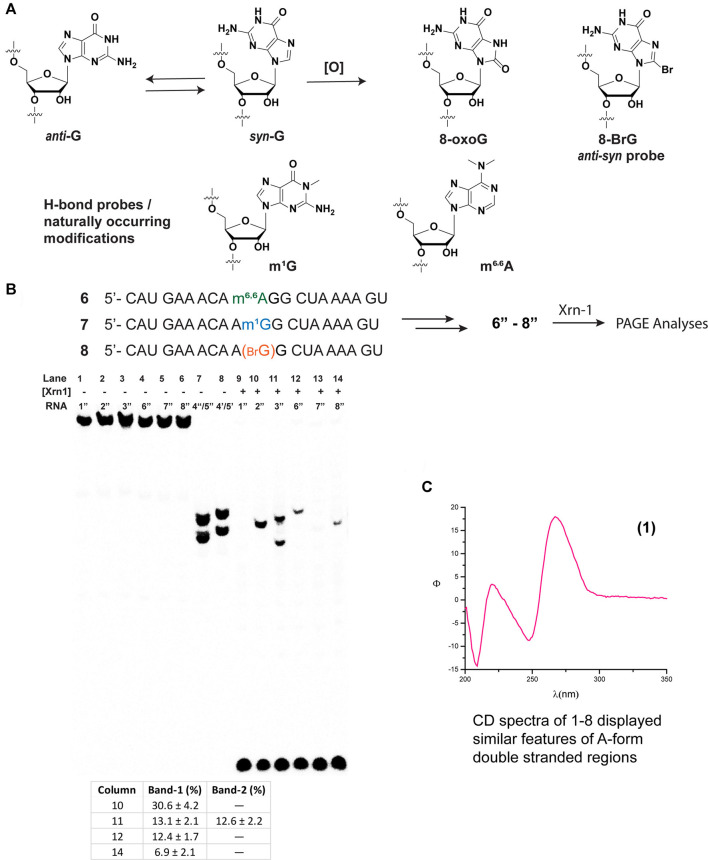
Structure and conformational changes of G and functionalized G/A with methyl groups (to probe H-bonding), or with bromine to probe for anti-syn conformational changes **(A)**; and their incorporation onto sequences **6**–**8** for their corresponding reactivity with xrn-1 and analyses *via* PAGE **(B)**. **(C)** displays the CD spectrum of RNA **1** in a buffered solution (4 μM RNA, 100 mM NaCl, 5 mM MgCl_2_, 10 mM sodium phosphate, pH 7.5).

We then proceeded to study the potential impact arising from spatial constraints, altered H-bonding, as well as conformational changes, that arise from the presence of a group at the C8-position, in this case an oxygen atom (Figure 2A). To this end, we chose to incorporate the following modifications: 1-methylguanine (m^1^G), *N,N*-6,6-dimethyladenine (m^6,6^A), and 8-bromoguanine (8-BrG). The former two are of interest given that 1) alkylative damage was recently explored alongside oxidative stress and m^1^G was one of the studied species; (Yan et al., 2019) 2) both m^1^G and m^6,6^A are naturally occurring modifications with essential biological functions, particularly in the case of the former; (McCown et al., 2020) and 3) the H-bonding pattern of these purine derivatives can serve to probe the hypothesis that, changes in H-bonding may play a role in the enzymatic stalling. Furthermore, 8-BrG is a chemically modified nucleoside that can be used to potentially provide information about conformational changes (*anti-syn*) around the glycosidic bond, (Skinner et al., 2020) given that substitution at the C8-position of purine rings has been shown to favor the equilibrium towards the syn-isomer. Thus, we proceeded to incorporate each of these chemical probes/modifications into the same sequence context and obtained ONs **6–8**, which were converted to radiolabeled-phosphorylated ONs **6**″–**8**″, and treated with Xrn-1 as discussed previously (Figure 2B). Electrophoretic analyses confirmed that the exoribonuclease stalls upon encountering 8-oxoG (Figure 2B, lanes 10–11); on the other hand, methylation of the N1-position of guanosine, only displayed the band corresponding to full degradation (Figure 2B, lane 13). Experiments carried out with ON **6**″, containing the dimethylated modification, resulted in the appearance of a slower band than that observed for 8-oxoG, consistent with exoribonuclease stalling upon encountering this modification in app. 12% yield (measured by the band intensity, lane 12). Combined, these observations suggest that space within the active site may be an important factor in the enzyme efficiency. Furthermore, the incorporation of 8-BrG (ON **8**″) also displayed the appearance of a band that travelled with the same speed as that observed for the ON containing 8-oxoG (Figure 2B, lane 14 compared to lane 10), albeit in a much lower yield than the other ONs described thus far. This suggests that the anti-syn conformational change that is expected to occur with RNA strands containing 8-oxoG may not be a relevant, or very minor, factor in the Xrn-1 stalling observed upon encountering the oxidative lesion.

Since Xrn-1 has been suggested to be involved in the degradation of damaged RNA, (Boo and Kim, 2020) the results that display enzyme stalling in the presence of 8-oxoG were surprising, therefore we considered structural parameters as a possible explanation. Although the canonical strand **1**″ resulted in a suitable Xrn-1 substrate, with efficient degradation, we considered two aspects that led us to probe for RNA structure; 1) that Xrn-1 induced degradation of RNA is halted upon encountering highly structured RNA (albeit much more structured than the 20-mers studied in this work); (Wastika et al., 2020) and 2) that the presence of one 8-oxoG lesion can result in structural changes even when using short oligonucleotides of RNA (Choi et al., 2017). A combination of CD with an established web server (Zuker, 2003) allowed us to establish the formation of secondary structure and predict the potential folds of ONs **1**–**3** and **6**–**8**. While the structural motifs predicted by UNAFOLD may be less accurate with longer sequences, we considered that this would provide a good framework for these short oligonucleotides. As shown on Figure 2C, CD spectra corresponding to RNA **1** (similar spectra were obtained for ONs **1**–**3**, **6**–**8**, Supplementary Figures S66–S71) displayed dichroic signals that are consistent with the formation of secondary structure with features corresponding to an A-form duplex, evident by the presence of a band with negative ellipticity at 210 nm. It is important to note that the relative intensity of the band at 210 nm with that at 238 nm also suggests the presence of single stranded regions, hence not a defined duplex or hairpin (Phillips and Bobst, 1972). This was corroborated upon measurement of the thermal denaturation transitions, which displayed non-sigmoidal curves that are consistent with the lack of a defined, thermally stable, secondary structure (Yang et al., 2007). Furthermore, predicted structures did not display hairpin motifs that were consistent with stalling of Xrn-1 upon encountering the first 8-oxoG lesion, position-11 (structures for **1**″– **3**″ are included in Supplementary Figure S83). Overall these observations suggest that the structure revealed by the CD spectra is not playing a role in the observed stalling.

**FIGURE 3:**
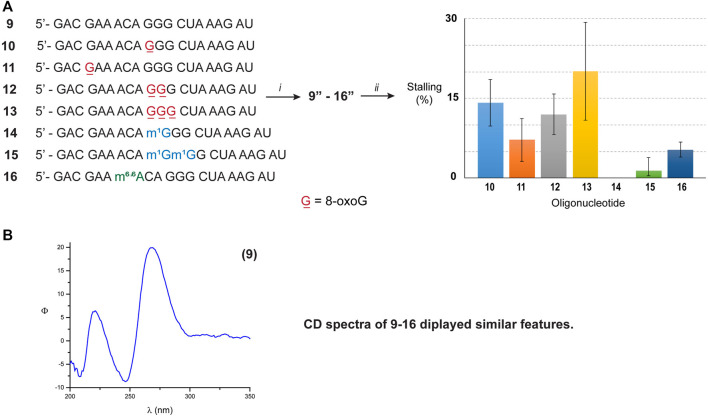
**(A)** Sequence of ONs **9**–**16**. The graph shows the measured intensity for the bands associated with exonuclease stalling. (*i*) T4-RNA ligase, bisphosphate **21**; then T4-PNK, ATP; (*ii*) xrn-1, then PAGE. **(B)** The CD spectrum corresponding to ON **9** was obtained using the following solution composition: (4 μM RNA, 100 mM NaCl, 5 mM MgCl_2_, 10 mM sodium phosphate, pH 7.5).

To corroborate this hypothesis, we opted to explore the reactivity of Xrn-1 using oligonucleotides of the same length and with a different sequence. The canonical RNA sequence **1** was altered (Figure 3A) in a manner that 1) no secondary structure was predicted, or measured; and 2) the number and position of 8-oxoGs could be further explored, while maintaining some sequence similarities. The design led to sequence **9**, which enabled us to explore the impact of exoribonuclease stalling as a function of position (ON **10**, **11**) and quantity of oxidative modifications (ONs **10**, **12**, **13**). Furthermore, this sequence was also modified to incorporate m^1^G and m^6,6^A in a manner to probe two subsequent alkylated modifications, ONs **14**–**16**. The lack of secondary structure was confirmed *via* CD analyses of ONs **9**–**16** (Figure 3B, bottom spectrum and Supplementary Figures S73–S75), which did not display features consistent with A-form double stranded regions. These results are also in agreement with data obtained from the UNAFold server, which predicted no possible folding with the parent sequence **9**. As expected, degradation experiments carried out with the canonical analogue **9** in the presence of Xrn-1, followed by electrophoretic analyses, displayed the presence of a fast moving band that was consistent with full degradation. On the other hand, reactions carried out with RNA strands containing 8-oxoG (ON **10–13**) displayed slower moving band(s) that were consistent with enzyme stalling at the site containing the oxidative modification (Supplementary Figures S84, S85). However, there were differences upon comparing with the previous sequence set in that 1) the overall percent stalling was lower, app. 12%, and this was independent of the number of modifications present; and 2) placing an oxidative lesion closer to the 5′-end (ON **11**) led to lower stalling at app. 5% (Figure 3, graph). As with the previous sequence set, RNA containing one or two m^1^G modifications (ON **14** and **15**) did not pose an obstacle for the exonuclease and reactions progressed to full degradation. On the other hand, the presence of the hypermethylated modification m^6,6^A (ON **16**) led to mild stalling with a band displaying app. 5% intensity.

**FIGURE 4:**
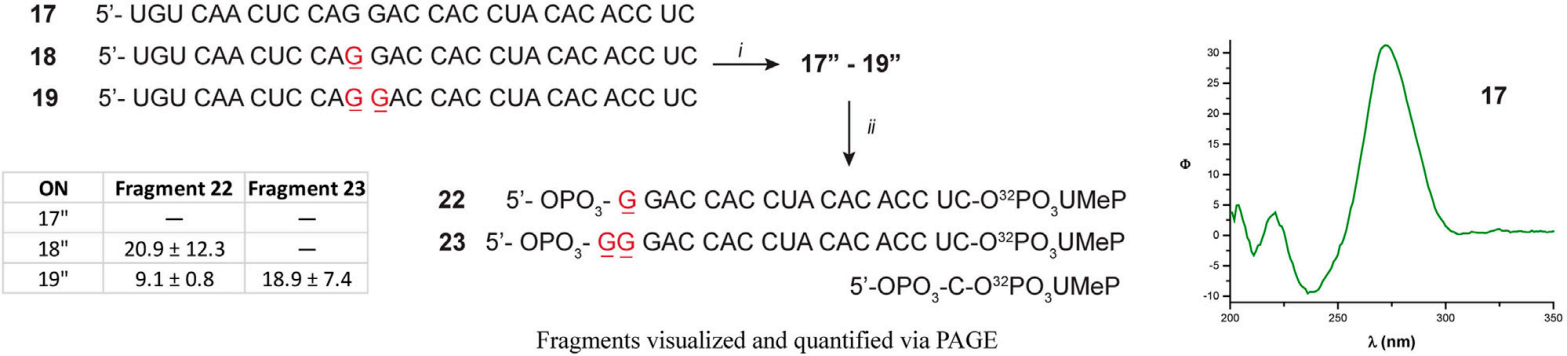
Sequence of oligonucleotides **17**–**19** followed by preparation to the corresponding **17**″-**19** analogues: (*i*) T4-RNA ligase, bisphosphate **21**; then T4-PNK, ATP; (*ii*) xrn-1, then dPAGE. The table inset shows the calculated yields for the observed fragments, corresponding to stalling at 8-oxoG.

Since there were differences in the efficiency of Xrn-1 to catalyze the hydrolysis of oxidized RNA, between the sequences **1**–**3** and **9**–**13**, the presence of some structural features (albeit not thermally stable) could not be ruled out at this stage. We then opted to probe a longer sequence, 29-nt-long (Figure 4). Oligonucleotides **17**–**19** have been previously reported, and do not display the formation of secondary structure (*via* CD, Figure 4 and Supplementary Figures S76–S78) (Glennon et al., 2020). These RNA sequences were used to probe for the presence of one or two consecutive 8-oxoG lesions within the sequence. In agreement with previous observations, stalling was also evident by the presence of one or two slower bands for ONs **18** or **19**, respectively, and in yields ranging 20-30%. The length of the observed fragments was corroborated by loading ON **1**″ alongside experiments carried out with the degradation experiments, corroborating that the site of interest corresponds to stalling at 8-oxoG (Supplementary Figures S86, S87). Overall, the observations confirm that Xrn-1 stalling is independent of secondary structure.

## Discussion

The efficiency of the exonuclease Xrn-1 was probed towards 20-mers of RNA containing 8-oxoG, and to our surprise, the exoribonuclease processing was affected by the presence of this oxidatively generated lesion. It is important to note that the observations described herein do not necessarily contradict previous findings correlating the role of Xrn-1 in the decay of oxidized mRNA, (Yan et al., 2019) particularly if one considers the vast differences in experimental conditions where the lack of several factors are not taken into consideration in the present work. However we propose that, given the presented results, it is very likely that other factors may play a role in the *in vivo* degradation of oxidized RNA, particularly that containing 8-oxoG. Thus, the aim of the present study focused on establishing a plausible reason for Xrn-1 stalling upon encountering 8-oxoG within RNA and provided data that will be of value when considering how oxidized RNA is handled intracellularly. The crystal structure of Xrn-1, from *Drosophila* containing a nucleic acid substrate (PDB entry 2Y35 (Jinek et al., 2011), contains a DNA TTT trinucleotide within the binding site and provided this work with a guideline to probe various factors. Specifically, this structure displays the potential importance of 1) π-π stacking interactions between the thymidine nucleobases within the binding pocket, and 2) the presence of Magnesium.

Overall, 8-BrG and m^6,6^A showed evidence of stalling albeit at much lower yields (app. 5%) than those observed with 8-oxoG (app. 6–30%). This suggests that spatial contributions may be relevant, but most likely not the main reason for exoribonuclease stalling at 8-oxoG. Furthermore, the presence of m^1^G at one or two consecutive positions did not have an impact on the processing by Xrn-1 which suggests that altered H-bonding, or spatial constraints, imposed by the presence of an N1-methyl group, is not relevant and that more drastic structural changes may be necessary to affect the efficiency of the exonuclease. It will be interesting to probe, in the future, on the impact that two consecutive m^6,6^A modifications has on the exonuclease activity. Varying [Mg^2+^] does not have an impact on the relative ratios of stalling and suggests that the processing differences are not due to interactions of 8-oxoG with the divalent cation. We did not probe variations in π-π stacking interactions, where the addition of a heteroatom (in this case Oxygen at the C8-position) has been reported to alter π-interactions within B-form duplexes, (Prat et al., 1998) also assigned to localized electronic changes (Crenshaw et al., 2011). These types of interactions may be different within the binding pocket of the enzyme, thus it is unclear at present about the stacking changes induced by the presence of 8-oxoG in this enzymatic context. It will be of interest, in the future, to obtain X-ray data of this and other ribonucleases containing oxidized RNA to aid in elucidating how 8-oxoG impacts reactivity and selectivity of these bio-systems.

Another important aspect that was considered, regards the presence of the stalled fragment in varying yields, dependent on the sequence of up to app. 30%. There are two possibilities that were considered, to explain the continuous presence of this fragment: 1) that the binding strength of the enzyme-RNA complex is high, and does not fully allow for the fragment to leave this state; or 2) that a 5′-phosphorylated RNA containing 8-oxoG (**4** in this case) is not a suitable substrate for Xrn-1 and inhibits its hydrolysis. We carried out mobility shift assay experiments using oligonucleotides **1**″-**3**″ as in Figure 1 under native conditions, however no slower moving band, that would indicate formation of a protein-RNA complex, was detected (Supplementary Figure S88). This indicates that the enzyme does not bind to the fragment where it is stalling, although more in-depth experiments are needed to corroborate this observation. On a different experiment, fragments **4**″ and **5**″ were treated with Xrn-1 to display degradation of the corresponding oligonucleotides, albeit not at the same efficiency than that observed with oligonucleotide **1**″. Although more experiments are needed in this regard, the results suggest that the 5′-phosphorylated-8-oxoG end may not be as efficient of a substrate as its canonical analogue.

## Conclusion

The efficiency of the exonuclease Xrn-1 was probed towards 20-mers and 30-mers of RNA containing one of the most relevant oxidative lesions, 8-oxoG. Surprisingly the exoribonuclease stalls upon encountering 8-oxoG to yield a 5′-phosphorylated fragment containing the lesion at this end. The reduced nuclease efficiency was found to be, in part, dependent on structural/conformational changes around the nucleoside. Other factors such as affected π-π stacking cannot be ruled out at the moment. These results were found to be independent of [Mg^2+^], or secondary structure (in this short oligonucleotides). The described results suggest that it is likely for other factors, besides Xrn-1, to be involved in the intracellular handling of oxidatively damaged RNA. This highlights the need to characterize, study, and better understand the exact mechanisms that control the processing of these RNA species. It is clear that 8-oxoG possesses distinct characteristics that pose a challenge on enzymatic processes, which makes such structural motif of potential interest. Efforts, from our group, are underway to assess the potential of analogous nucleotides as ribonuclease inhibitors.

## Data Availability

The original contributions presented in the study are included in the article/Supplementary Material, further inquiries can be directed to the corresponding author.
