# Supplementary material for: Processing of RNA Containing 8-Oxo-7,8-Dihydroguanosine (8-oxoG) by the Exoribonuclease Xrn-1
Source: Front Mol Biosci. 2021 Nov 15;8:780315. doi: 10.3389/fmolb.2021.780315 (PMC8634602; doi:10.3389/fmolb.2021.780315)
Supplement: Supplementary file 1 [file DataSheet1.PDF]

Processing of RNA containing 8-oxo-7,8-dihydroguanosine (8-oxoG) by the exoribonuclease Xrn-1

Cheyenne N. Phillips[ǂ§], Shawn Schowe[ǂ§], Conner J. Langeberg,[¥] Namoos Siddique[ǂ], Erich G. Chapman[¥], Marino J. E. Resendiz[ǂ]*

[ǂ]Department of Chemistry, University of Colorado Denver, Science Building 1151 Arapahoe St, Denver, CO

80204, USA

[¥]Department of Chemistry, University of Denver, 2190 E. Iliff Ave, F.W Olin Hall, Room 202, Denver, CO

80210, USA

[§]These authors contributed equally to this work.

* To whom correspondence should be addressed. Tel: 303-315-7658 ; Email: marino.resendiz@ucdenver.edu,

Supporting Information Index:

Page: **Contents:**

S3-S11………..………Experimental procedures for the synthesis of the m[1]G phosphoramidite: Figure S1-S18 include ^{1}H / ^{13}C / ^{31}P NMR, and IR spectra of all intermediates.

S12-17………………...Experimental protocol for the synthesis of the m_2^6A phosphoramidite: Figure S19-S29 include ^{1}H / ^{13}C / ^{31}P NMR, and IR spectra of all intermediates.

S18-26………………...Experimental protocol for the synthesis of the 3'-methylphosphate **20**: Figure S30-S46 include ^{1}H / ^{13}C / ^{31}P NMR, and IR spectra of all intermediates.

S27…………………....Sequence table corresponding to ONs **1-19**

S28-S33……………….MALDI TOF MS of ONs **1-16**; Figure S47-S63

S33…………………....Full MALDI-TOF corresponding to Figure 1-D (in manuscript); Figure S64

S34…………………....MALDI-TOF MS displaying the reaction between ON 3 and Xrn-1; Figure S65.

S35-S36……………….CD spectra of sequences **1-3** and **6-8** along with thermal denaturation transition measurements of applicable sequences; Figure S66-S71

S37……………….……CD spectra overlay of sequences **1-3** and **6-8**; Figure S72

S38……………….……CD spectra of sequences **9-14**; Figure S73-S75

S39…………………....CD spectra of sequences **17-19**; Figures S76-S78.

S40…………………....PAGE experiments for the 3'-labeling of various ONs; Figure S79

S41…………………....Time dependent experiment using ONs **1"-3"**, Figure S80
[Xrn-1] dependent experiment using ONs **1"-3"**, Figure S81

S42……………………Experiment as a function of [Mg^{2+}], using ONs **2"**& **3"**; Figure S82.

S43……………………Predicted Structures of ONs **1”-3”**, Figure S83.

S44…………………… Time dependent experiment using ONs **9”-16”**, Figure S84 & S85.

S45.………………….... [Xrn-1] dependent experiment using ONs **17”-19”**, Figure S86 & S87.

S46……………………Experiments using native conditions and ONs **1”-3”**; Figure S88.

S47……………………Treatment of ONs **1**”-**8”** with Xrn-1 using dPAGE; Figure S89.

S48………………..…..References

General Information. All Reagents were purchased and used without further purification. DMF, DCM, DIPEA, pyridine were distilled over calcium hydride and THF was distilled over benzophenone and sodium. All NMR spectra were taken on either the Bruker AV-III 400 MHz NMR or the Bruker AV-III 300 MHz NMR. All FTIR spectra were gained using the Nicolet iS5 FTIR spectrometer. High resolution mass spec were obtained using the Agilent 6500 series Q-TOF LC/MS system.

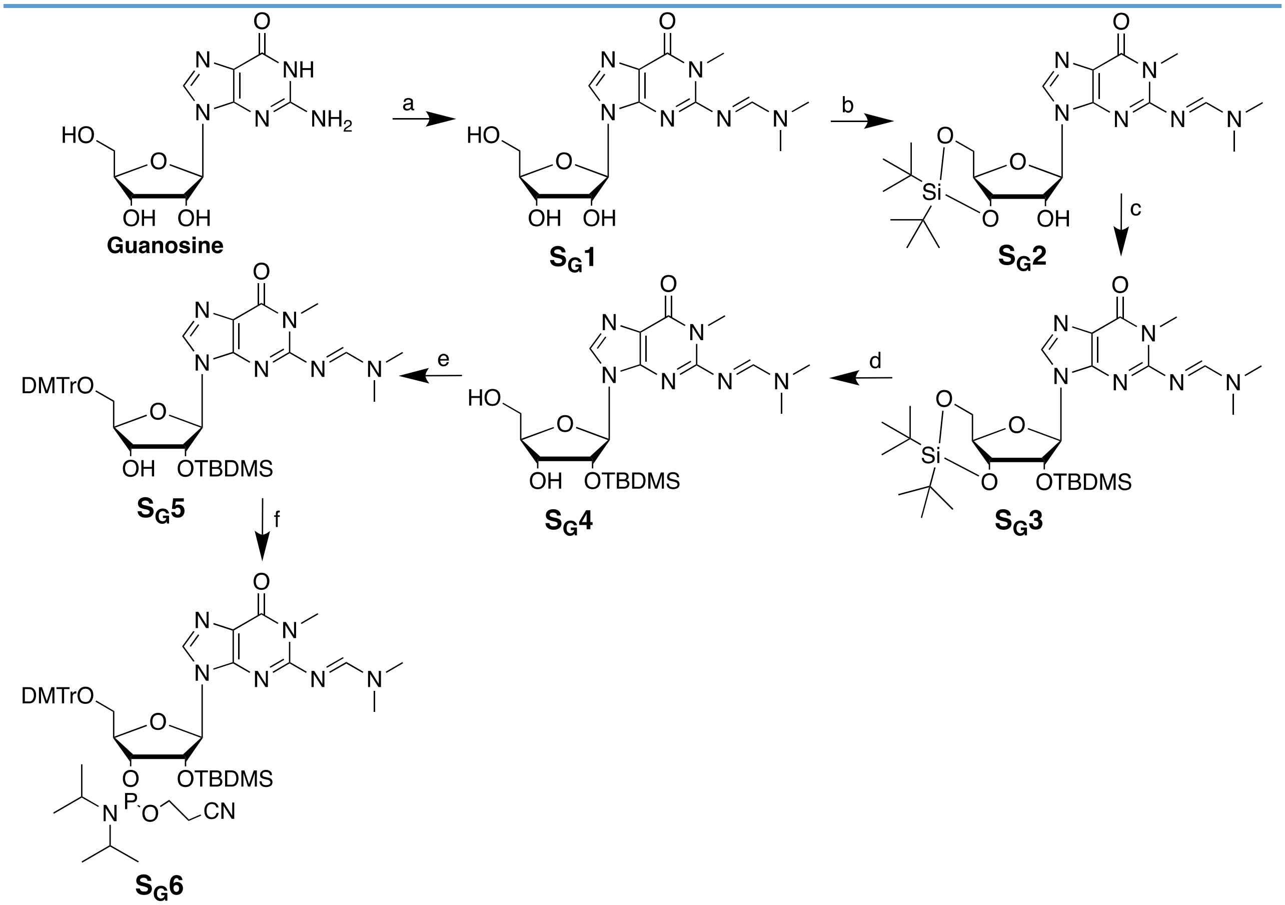

Scheme S1. *Synthesis of the m*[1]*G phosphoramidite* ***SG6***; (a) *N,N*-dimethylformamide dimethyl acetal, DMF, 90 °C, 16 h, **SG1**: 91%; (b) di-tert-butylsilyl bis(trifluoro methanesulfonate), imidazole, pyridine, 0 °C, 0.5h, **SG2**: 87 %; (c) tert-butyldimethylsilyl triflouromethanesulfonate, imidazole, DMF, 0 °C → rt, 12h, **SG3**: 90 %; (d) Triethylamine trihydrofluoride, THF, pyridine, 0 °C, 20 min, **SG4**: 58%; (e) 4,4'- dimethoxy triphenylmethyl chloride, pyridine, 0 °C → rt, 7h, **SG5**: 45.5%; (f) 2- cyanoethyl-*N,N*-diisopropylchlorophosphoramidite, DIPEA, DCM, 1.25h, **SG6**: 72%.

2-*N*-(dimethyl formamide)- *1-N*-methylguanosine (SG1)

Guanosine (4g, 14.1 mmol), was dissolved in DMF (90mL) and *N,N*-dimethylformamide dimethyl acetal (20 mL, 150.6 mmol) with stirring at 90 °C for 4 h. An additional 10 mL of *N,N*-dimethylformamide dimethyl acetal (10 mL, 75.3 mmol) was added with stirring for 12 h. The Organic residue was concentrated under reduced pressure and purified using flash column chromatography (100 % DCM, slow gradient to 30 % MeOH in DCM). **SG1** was observed as a white foam (4.5 g, 91 %, 12.8 mmol). IR (cm^{-1}): 3298, 3108, 2926, 2360, 1672, 1625, 1577; ^1H NMR (400 MHz, DMSO-d6) δ 8.54 (s, 1H), 8.06 (s, 1H), 5.80 (d, *J*=6, 1H), 5.41 (d, *J*=6, 1H), 5.18 (d, *J*=5, 1H), 5.01 (t, *J*=11, 1H), 4.51-4.47 (m, 1H), 4.14-4.11 (m, 1H), 3.92-3.89 (m, 1H), 3.66-3.61 (m,

1H), 3.57-3.52 (m, 1H), 3.49 (s, 3H), 3.20 (s, 3H), 3.09 (s, 3H); ^{13}C NMR (100 MHz, DMSO) δ 157.6, 157.4, 156.8, 148.0, 137.2, 118.8, 86.6, 85.4, 73.8, 70.4, 61.5, 40.8, 34.8, 29.4 ppm. HRMS m/z calculated for $C_{14}H_{20}N_6O_5$ (M^++H), 353.1568, observed m/z = 353.1563.

2-*N*-(dimethyl formamide)- 3',5'-O-bis-(t-butylsilyl)-1-*N*-methylguanosine (SG2)

Nucleoside **SG1** (2.0483 g, 5.8 mmol) was azeotropically dried over pyridine (10 mL, x2) and redissolved in DMF (15 mL). The solution was cooled to 0 °C followed by the addition of di-tert-butylsilyl bis(trifluoromethanesulfonate) (2 mL, 6.4 mmol) with stirring for 30 min. The reaction was allowed to warm to rt with stirring for an additional 30 min. Imidazole (5 g, 73.4 mmol) was added at 0 °C and stirred for 5 min. The organic residue was concentrated under reduced pressure. The yellow solid was dissolved in ethyl acetate and partitioned with H_2O and ethyl acetate (10 mL, x3). Organics were washed then dried over brine and sodium sulfate respectively. Purification using flash column chromatography (100 % DCM, then slow gradient to 5% MeOH in DCM). **SG2** was isolated as a white foam (2.5 g, 5.07 mmol, 87 %). IR (cm^{-1}): 3100, 2931, 2858, 2603, 2497, 2360, 1679, 1625, 1574, 1532; ^{1}H NMR (400 MHz, DMSO-d6) δ 8.48 (s, 1H), 8.01 (s, 1H), 5.84 (s, 1H), 5.79 (d, *J*=4, 1H), 4.57-4.54 (m, 1H), 4.37-4.32 (m, 2H), 4.03-3.92 (m, 2H), 3.47 (s, 3H), 3.18 (s, 3H), 3.07 (s, 3H), 1.04 (s, 9H), 1.01 (s, 9H); ^{13}C NMR (100 MHz, DMSO) δ 157.4, 157.3, 156.9, 147.4, 137.5, 118.8, 89.8, 76.3, 73.7, 72.7, 66.9, 40.8, 34.8, 29.4, 27.2, 27.0, 22.2, 20.1 ppm. HRMS m/z calculated for $C_{22}H_{36}N_6O_5Si$ (M^++ H), 493.2589, observed m/z = 493.2595

2-*N*-(dimethyl formamide)-2'-O-(t-butyldimethylsilyl)-3',5'-O-bis-(t-butylsilyl)-1-*N*- methylguanosine (SG3)

A round bottom flask (RBF) was charged with nucleoside **SG2** (1.81 g, 3.67 mmol) and imidazole (5 g, 73.4 mmol) and azeotropically dried over pyridine (15 mL, x2). The solid was redissolved in DMF (40 mL) and cooled to 0 °C followed by dropwise addition of tert-butyldimethylsilyl triflouromethanesulfonate (8.45 mL, 36.8 mmol) over 4 min and stirred for 1 h, after this, the solution was warmed to rt over 10 h. The solution was concentrated under reduced pressure. The residue was partitioned in H_2O and ethyl acetate (10 mL, x3). The organics were washed then dried over brine and sodium sulfate respectively. Purification by flash column chromatography (slow gradient to 90% EtOAc in hexanes, then 100% EtOAc) to afford **SG3** as a white foam (2.013 g, 90 %, 3.32 mmol). IR (cm^{-1}): 2932, 2858, 2362, 1698, 1677, 1626, 1575, 1533; ^{1}H NMR (400 MHz, $CDCl_3$) δ 8.54 (s, 1H), 8.26 (s, 1H), 5.97 (s, 1H), 4.52-4.46 (m, 2H), 4.25-4.08 (m, 3H), 3.65 (s, 3H), 3.22 (s, 3H), 3.19 (s, 3H), 1.06 (s, 9H), 1.04 (s, 9H), 0.15 (s, 6H); ^{13}C NMR (100 MHz, $CDCl_3$) δ 158.5, 157.9, 156.9, 146.9, 136.0, 91.5, 77.4, 76.2, 75.9, 74.8, 67.8, 41.6, 35.6, 30.3, 27.6, 27.2, 26.1, 22.8, 20.5, 18.5, -4.1, -4.8 ppm. HRMS m/z calculated for $C_{28}H_{50}N_6O_5Si_2$ (M^++H), 607.3454, observed m/z = 607.3460.

2-*N*-(dimethyl formamide)-2'-O-(t-butyldimethylsilyl)-1-*N*-methylguanosine (SG4)

THF (25 mL) was added to a flask containing nucleoside **SG3** (2.01 g, 3.3 mmol) and cooled to 0 °C. A solution of triethylamine trihydroflouride (0.3 mL, 1.8 mmol) and pyridine (2 mL) and added dropwise over a period of 20 min with stirring at 0 °C. The reaction mixture was neutralized with slow addition of 15 % $NaHCO_3$ (10 mL) at 0 °C. The solution was partitioned in H_2O (10 mL) and ethyl acetate (10 mL x3). Organics were washed then dried over brine and sodium sulfate respectively. Purification using flash column chromatography (slow gradient to 90% DCM in hexanes, then 100% DCM, followed by slow gradient to 5 % MeOH in DCM) to isolate **SG4** as a white foam (0.9 g, 58 %, 2.19 mmol). IR (cm^{-1}): 3351, 3214, 3118, 2934, 2896, 2853, 1672, 1624, 1574; ^{1}H NMR (400 MHz, DMSO-d6) δ 8.51 (s, 1H), 8.12 (s, 1H), 5.83 (d, *J*=5, 1H), 5.10 (t, *J*=11, 1H), 5.07 (d, *J*=5, 1H), 4.54-4.51 (m, 1H), 4.15-4.12 (m, 1H), 3.95-3.93 (m, 1H), 3.73-3.68 (m, 1H), 3.62-3.57 (m, 1H), 3.49 (s, 3H), 3.18 (s, 3H), 3.09 (s, 3H), 0.78 (s, 9H), -0.045 (s, 3H), -0.12 (s, 3H); ^{13}C NMR (100 MHz,

DMSO-d6) δ 157.4, 156.8, 147.7, 137.0, 118.8, 87.1, 85.1, 75.9, 69.9, 60.9, 54.9, 40.8, 34.8, 29.4, 25.6, 17.9, -4.90, -5.23 ppm. HRMS m/z calculated for $C_{20}H_{34}N_6O_5Si$ (M^++H), 467.2433, observed m/z = 467.2437.

2-*N*-(dimethyl formamide)-2'-O-(t-butyldimethylsilyl)- 5'-O-(4,4'-dimethoxytrityl)-1- *N*-methylguanosine (S_G5)

Nucleoside **S_G4** (0.754 g, 1.6 mmol) was azeotropically dried over pyridine (4 mL) the solid was redissolved in pyridine (20 mL) and the solution was cooled to 0 $^{\circ}$C. 4,4'- dimethoxytriphenylmethyl chloride (0.59 g, 1.7 mmol) was added at once and allowed to stir for 1 h. The solution was warmed to rt slowly and left for 5 h. The solution was partitioned with 15% $NaHCO_3$ and ethyl acetate (3 mL, x3). The organics were washed then dried with brine and sodium sulfate respectively. Flash column chromatography (slow gradient to 50% EtOAc in hexanes, then 100 % EtOAc, followed by slow gradient to 0.4 % MeOH in EtOAc), yielded **S_G5** as a white foam (0.56 g, 0.73 mmol, 45.5 %). IR (cm^{-1}): 3350, 2928, 2854, 2360, 2340, 1673, 1626, 1574, 1532; ^{1}H NMR (400 MHz, $CDCl_3$) δ 8.50 (s, 1H), 7.81 (s, 1H), 7.45-7.17 (m, 9H), 6.84-6.81 (m, 4H), 5.99 (d, *J*=6, 1H), 4.73-4.71 (m, 1H), 4.34-4.32 (m, 1H), 4.23-4.21 (m, 1H), 3.79 (s, 6H), 3.67 (s, 3H), 3.50-3.46 (m, 1H), 3.40-3.37 (m, 1H), 3.12 (s, 3H), 3.09 (s, 3H), 2.82 (s, 1H), 0.87 (s, 9H), 0.037 (s, 3H), -0.11 (s, 3H); ^{13}C NMR (100 MHz, $CDCl_3$) δ 158.5, 157.4, 157.2, 148.2, 144.6, 135.6, 130.2, 130.1, 129.2, 128.2, 128.0, 127.1, 113.3, 113.2, 86.7, 83.7, 76.5, 71.8, 63.7, 55.3, 41.1, 35.2, 30.0, 25.7, 18.0, -4.8, -5.1 ppm. HRMS m/z calculated for $C_{41}H_{52}N_6O_7Si$ (M^++ H), 769.3740, observed m/z = 769.3744.

2-*N*-(dimethyl formamide)-2'-O-(t-butyldimethylsilyl)-3'-O-(2-ethylcyano-*N,N*-diisopropylphosphoramidite)-5'-O-(4,4'-dimethoxytrityl)- *1-N*-methylguanosine (S_G6)

An RBF was charged with nucleoside **S_G5** (0.33 g, 0.43 mmol), to which DCM (1.2 mL), DIPEA (0.44 mL, 2.5 mmol), and 2- cyanoethyl-*N*,*N*-diisopropylchloro phosphoramidite (0.14 mL, 0.63 mmol) were added and stirred at rt for 1.25 h. 2- cyanoethyl-*N*,*N*-diisopropylchloro phosphoramidite (0.05 mL, 2.3 mmol) was added followed by 10 min of stirring. The reaction was partitioned 50% $NaHCO_3$ and DCM (2 mL, x3). The organics were washed then dried with brine and sodium sulfate respectively. Purification by flash column chromatography (100% DCM 2x, then slow gradient to 30 % acetone in DCM) **S_G6** was isolated as a white foam (0.3 g, 0.31 mmol, 72 %). IR (cm^{-1}): 2963, 2929, 2858, 2360, 2340, 1688, 1626, 1574, 1530; ^{1}H NMR (300 MHz, $CDCl_3$) δ 8.52 (s, 1H), 8.47 (s, 1H), 7.86 (s, 1H), 7.82 (s, 1H), 7.46-7.15 (m, 18H), 6.83-6.79 (m, 8H), 6.02-5.89 (m, 2H), 4.38-2.58 (m, 48H), 1.47 (d, *J*=7, 6H), 1.29-1.15 (m, 16H), 1.01 (d, *J*=7, 6H), 0.81-0.79 (m, 18H), 0.11-0.32 (m, 12 H); ^{31}P NMR (122 MHz, $CDCl_3$) δ 150.7, 149.6 ppm. HRMS m/z calculated for $C_{50}H_{69}N_8O_8PSi$ (M^++H), 969.4818, observed m/z = 969.4844.

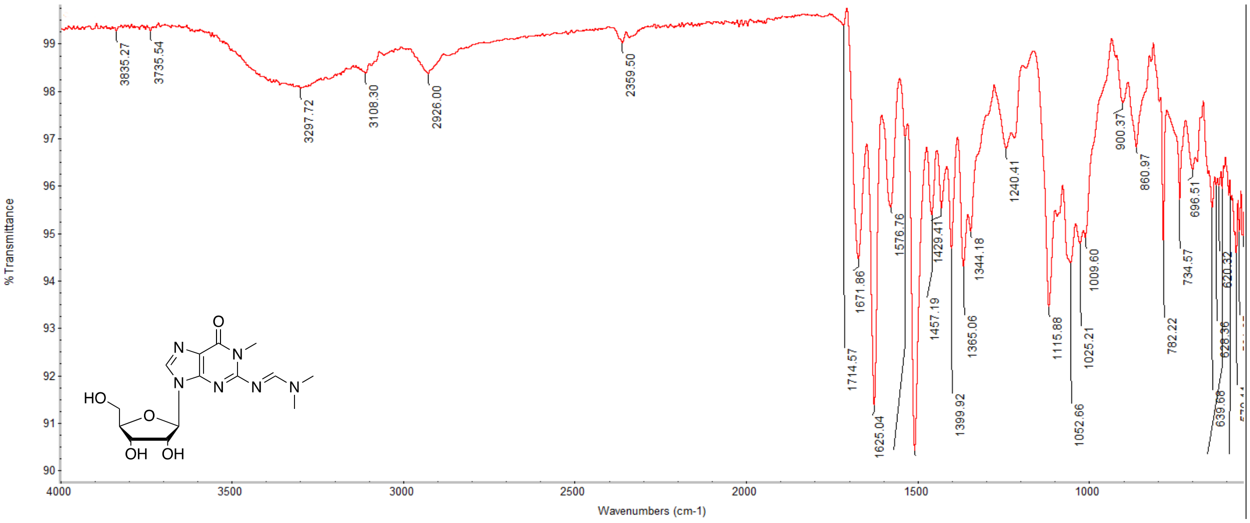

Figure S1. IR spectrum of **SG1**

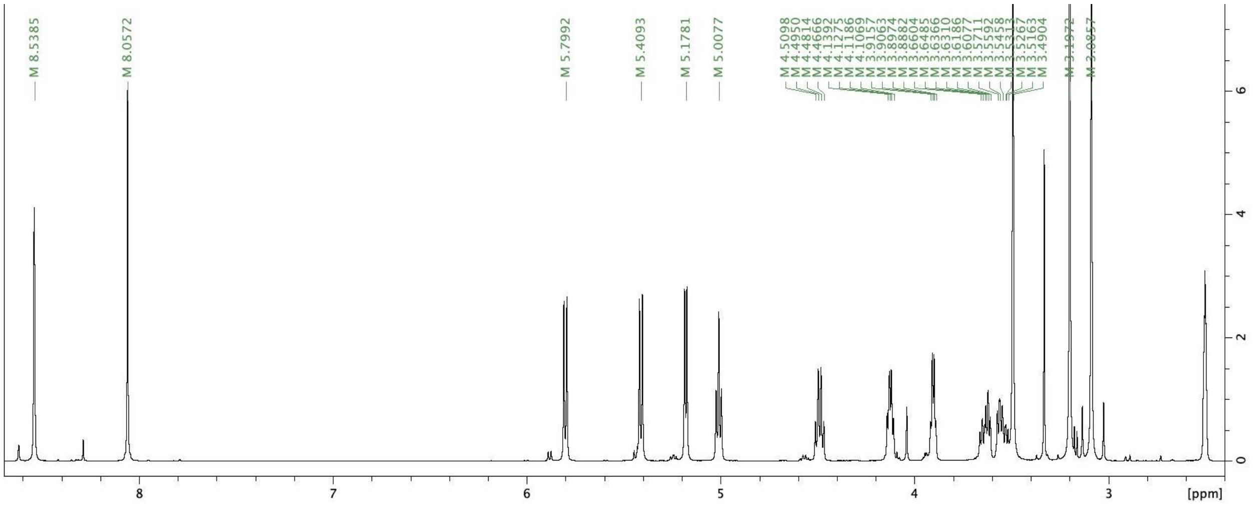

Figure S2. ^{1}H NMR of compound **SG1**

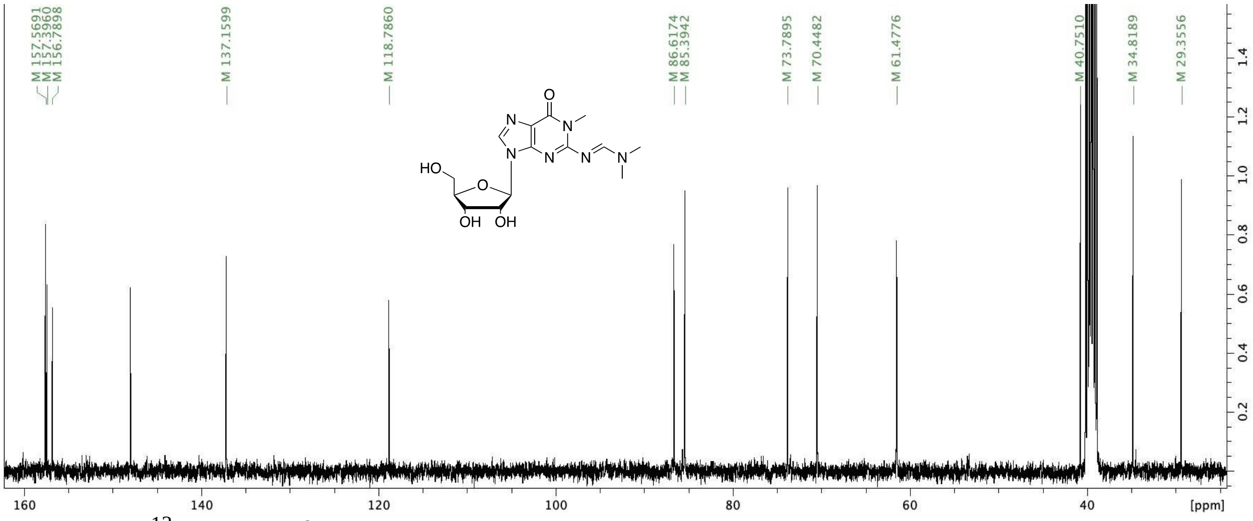

Figure S3. ^{13}C NMR of compound **SG1**

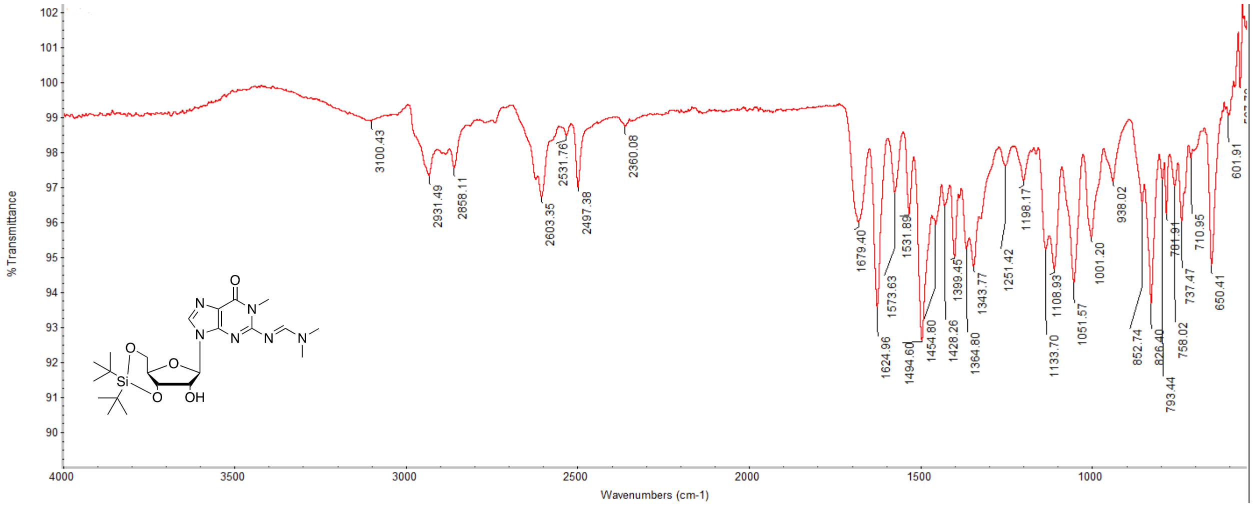

Figure S4. IR spectrum of **SG2**

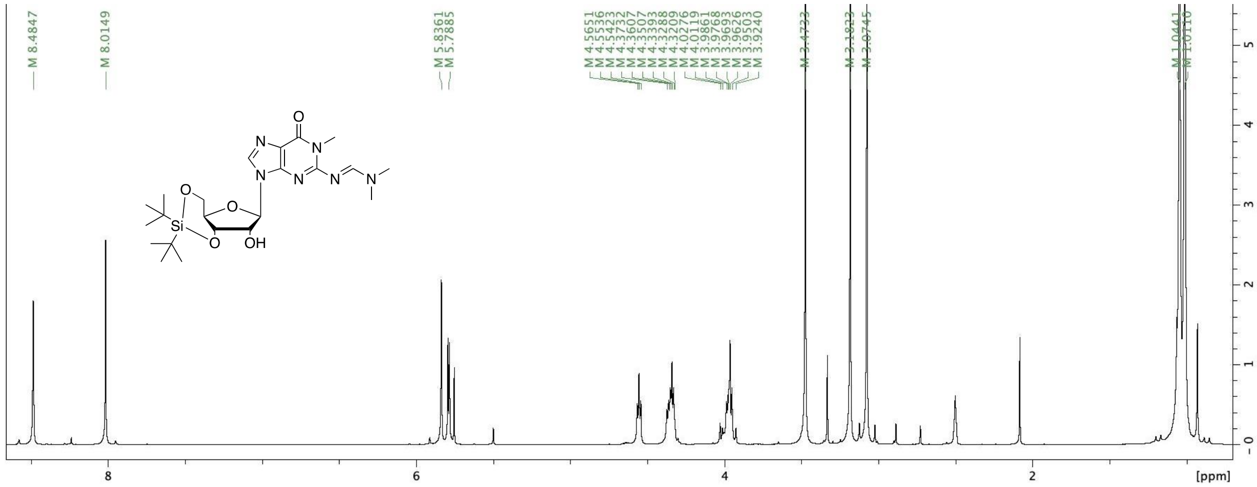

Figure S5. ^{1}H NMR of compound **SG2**

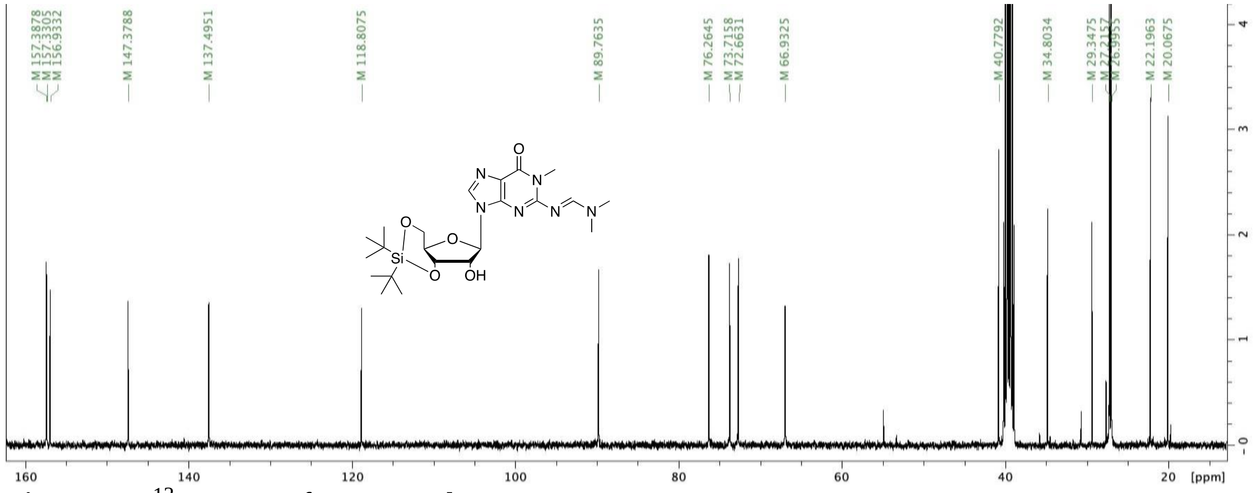

Figure S6. ^{13}C NMR of compound **SG2**

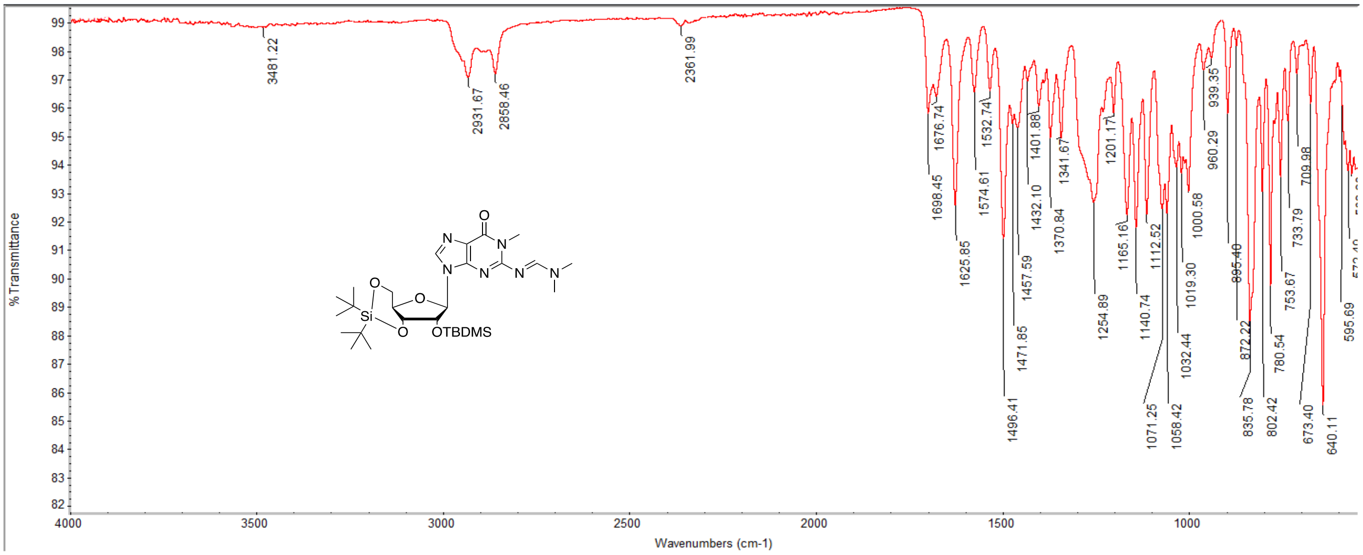

Figure S7. IR spectrum of **SG3**

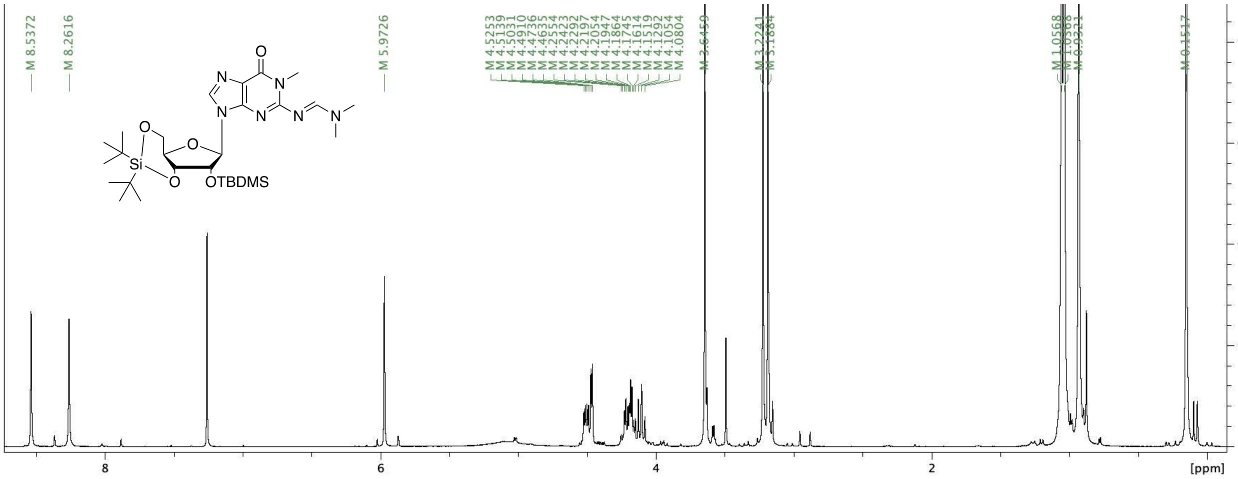

Figure S8. ^{1}H NMR of compound **SG3**

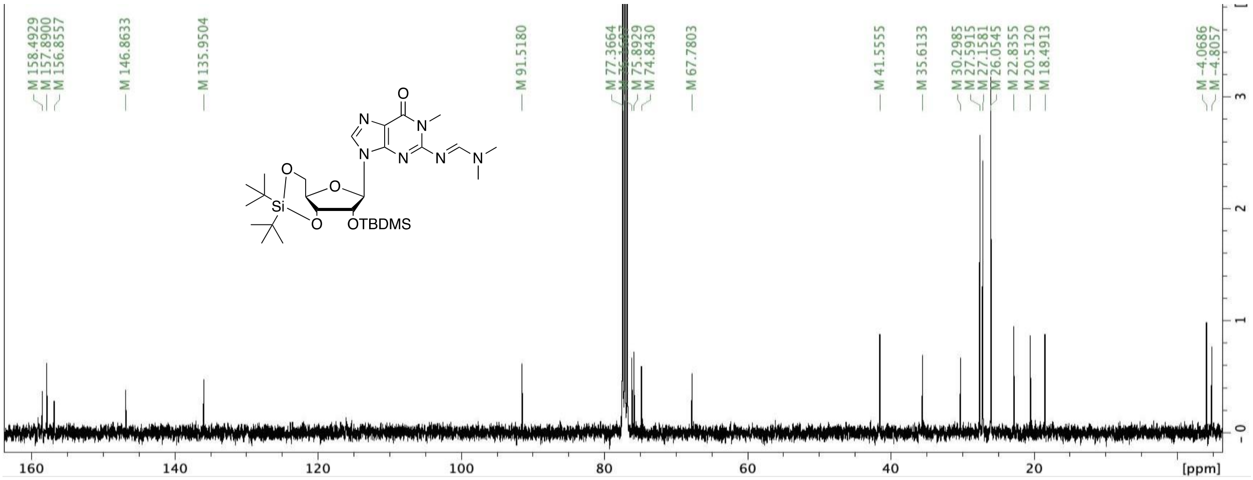

Figure S9. ^{13}C NMR of compound **SG3**

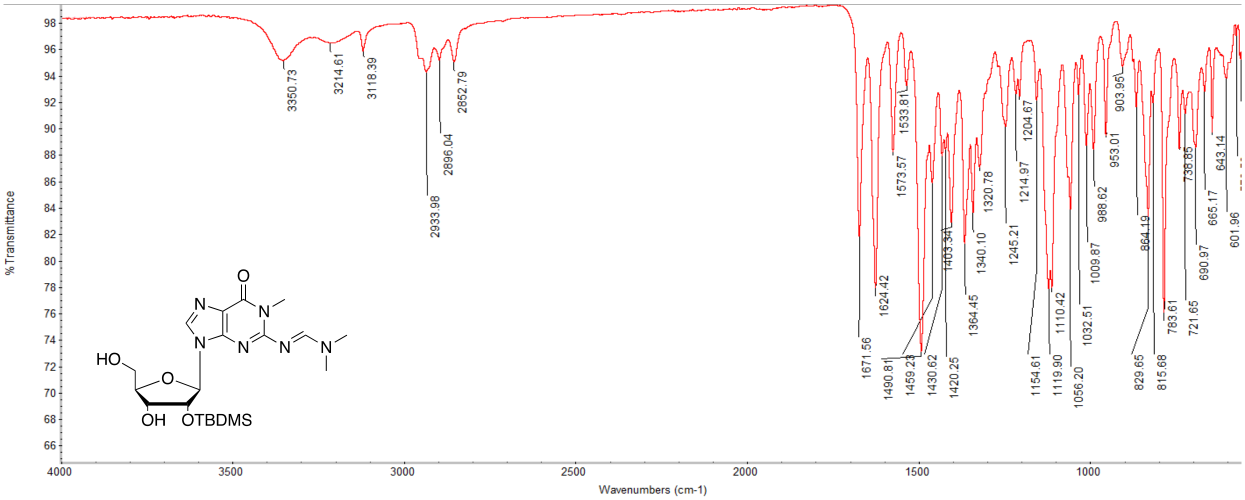

Figure S10. IR spectrum of **SG4**

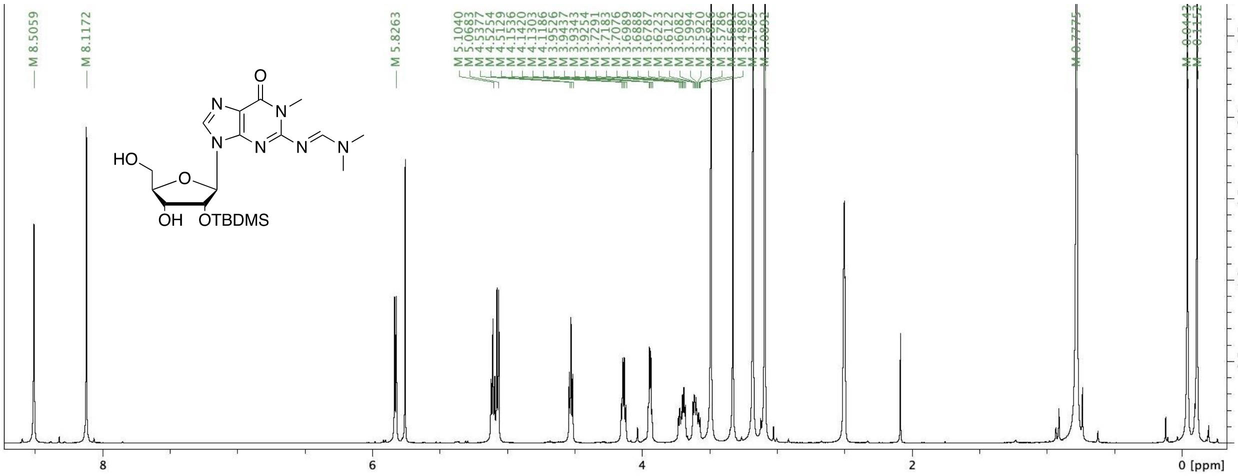

Figure S11.^{1}H NMR of compound **SG4**

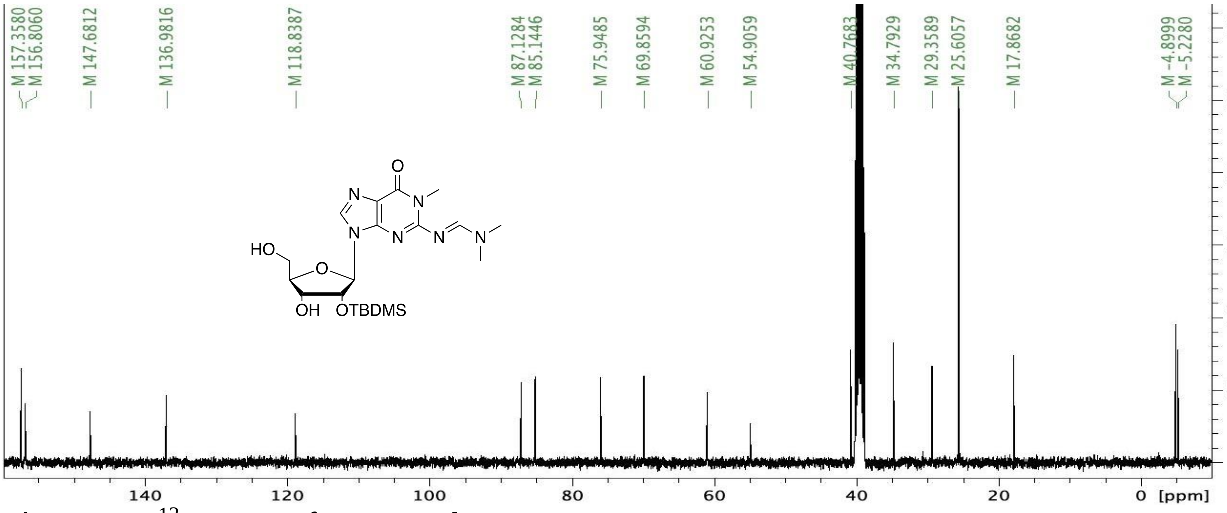

Figure S12.^{13}C NMR of compound **SG4**

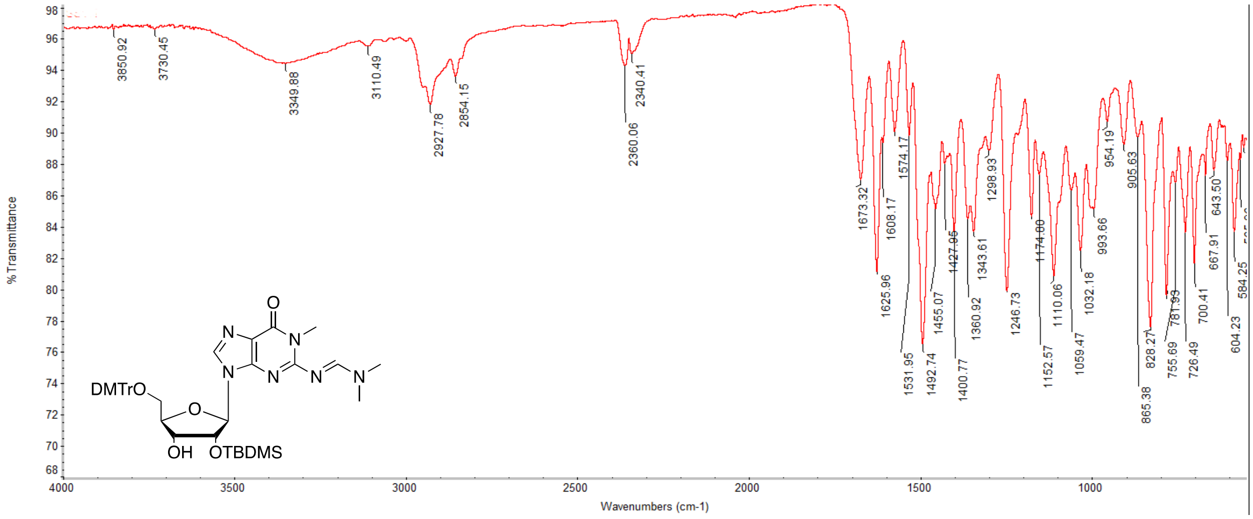

Figure S13. IR spectrum of **S$_G$5**

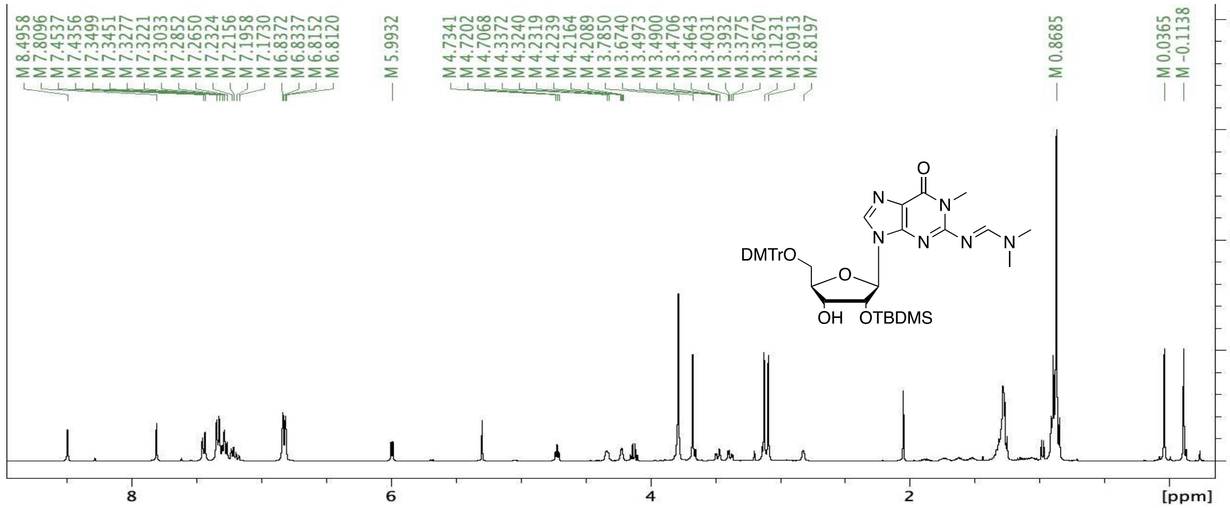

Figure S14.¹H NMR of compound **S$_G$5**

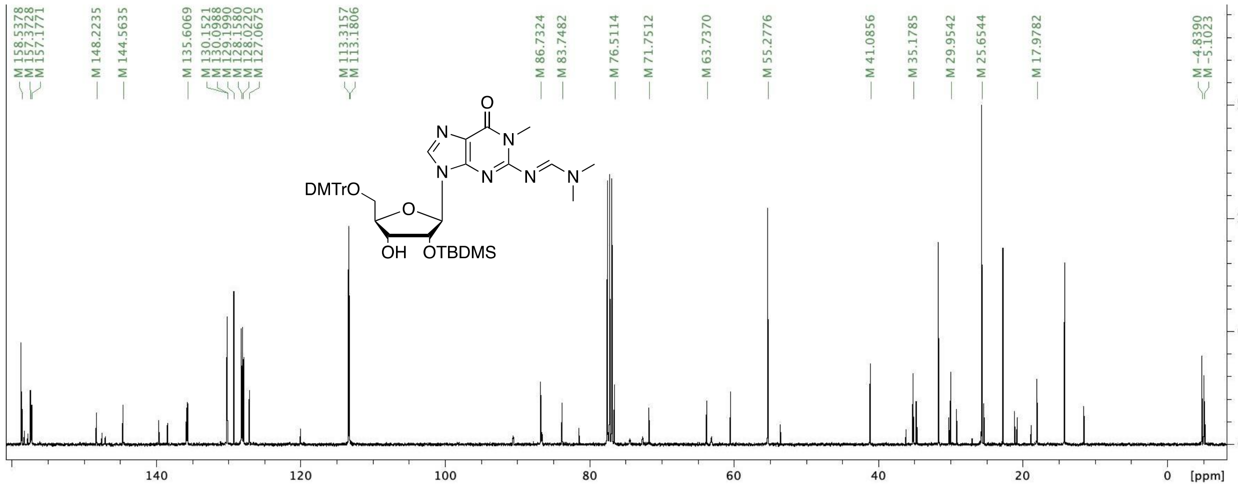

Figure S15.¹³C NMR of compound **S$_G$5**

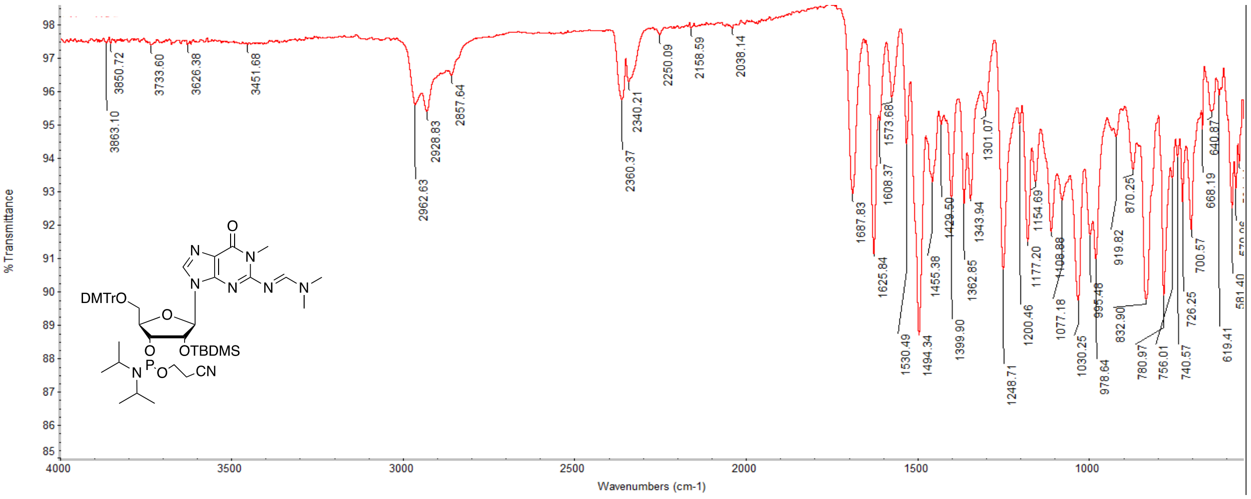

Figure S16. IR spectrum of **SG6**

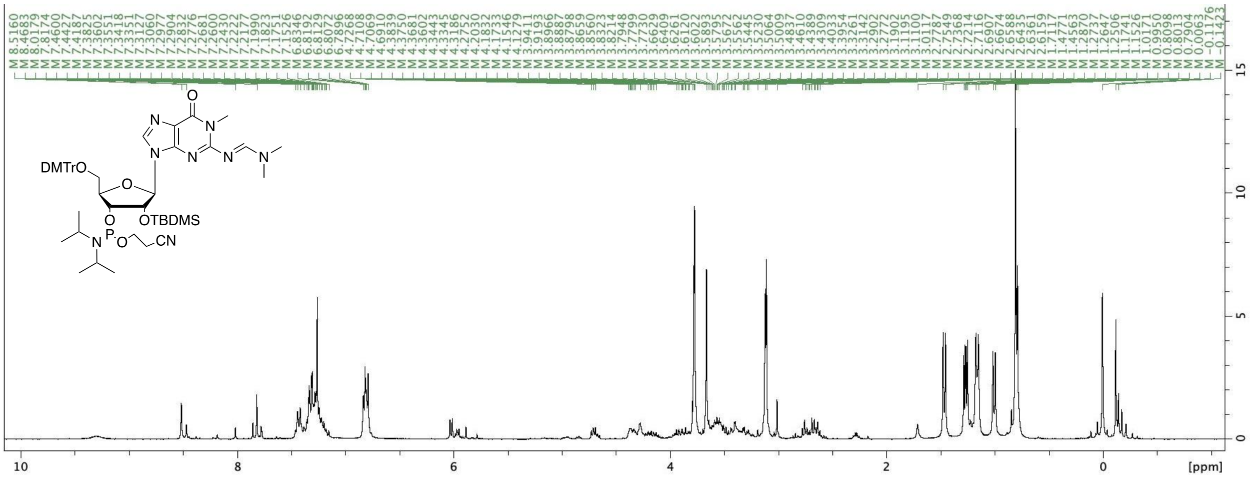

Figure S17. ^{1}H NMR of compound **SG6**

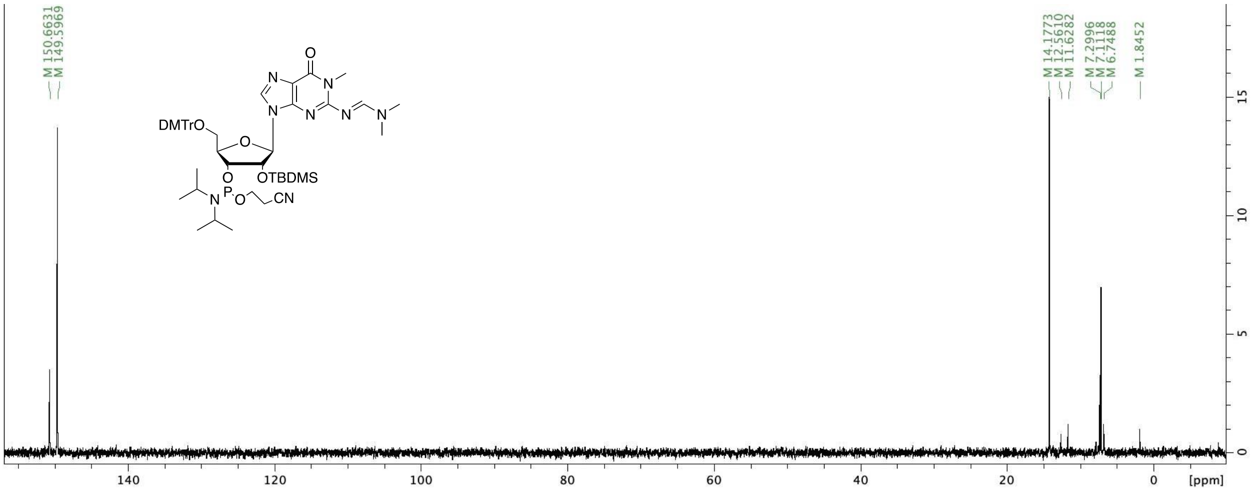

Figure S18. ^{31}P NMR of compound **SG6**

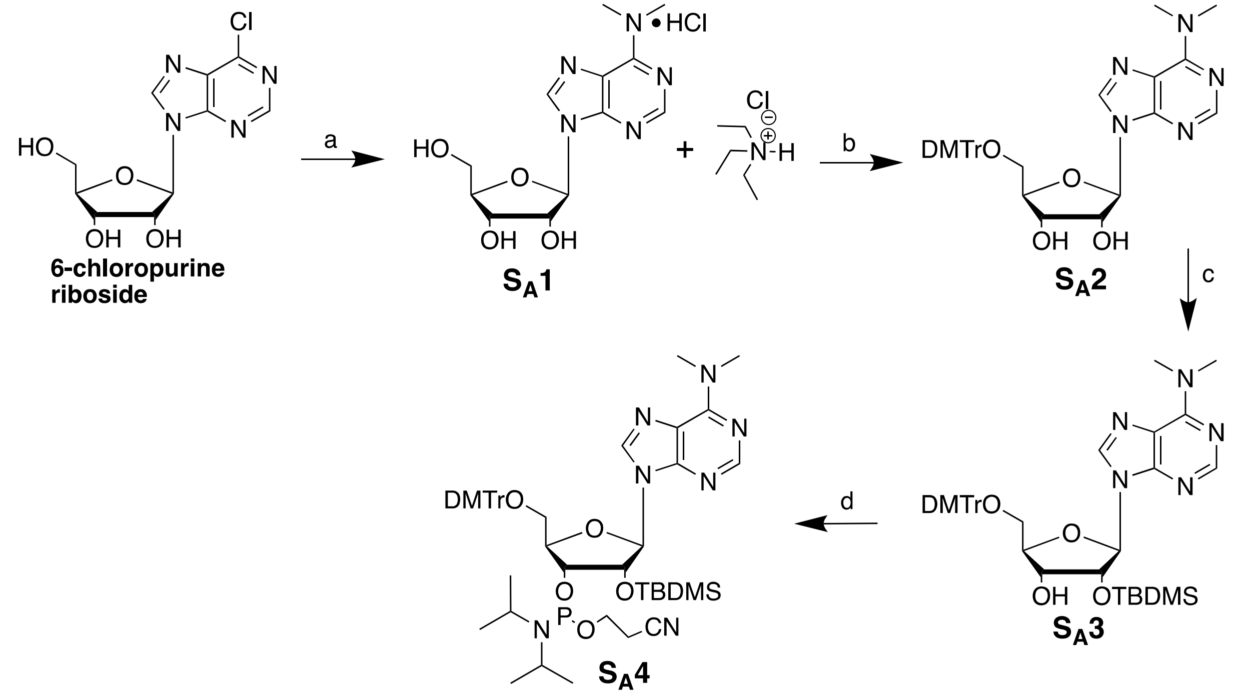

Scheme S2. *Synthesis of $m^{2,6}$A phosphoramidite **S_A4***; (a) $(CH_3)_2NH$•HCl, TEA, ethanol, reflux for 18h, **S_A1**: 80%; (b) 4,4'- dimethoxytriphenylmethyl chloride, pyridine, 0 °C → rt, 7h, **S_A2:** 55%; (c) tert-butyldimethylsilyl chloride, $AgNO_3$, pyridine, THF, rt, 18.5h, **S_A3:** 40%; (d) 2-cyanoethyl-*N,N*-diisopropylchlorophosphoramidite, DIPEA, DCM, 1.25h, **S_A4**: 78%.

6-*N,N*-dimethyladenosine (S_A1)[1]

An RBF was charged with 6-chloropurine riboside (2 g, 6.98 mmol) and $(CH_3)_2NH$•HCl (0.63 g, 7.7 mmol) was dissolved in ethanol (90 mL) and triethylamine (1.1 mL, 7.8 mmol) with stirring at 95 °C under reflux for 30 h. Organic residues were concentrated under reduced pressure and purified via flash column chromatography (100 % DCM, then slow gradient to 20 % MeOH in DCM) to afford **S_A1** as a white foam (2.6 g, 80%, 5.6 mmol). IR (cm^{-1}): 3396, 3296, 3075, 2927, 2601, 2495, 2360, 1607; ^{1}H NMR (400 MHz, DMSO-d6) δ 9.93 (s, Br, 1H), 8.37 (s, 1H), 8.21 (s, 1H), 5.91 (d, *J*=6, 1H), 5.43 (d, *J*=6, 1H), 5.34 (t , *J*=11, 1H), 5.17 (d, *J*=5, 1H), 4.59-4.55 (m, 1H), 4.16-4.13 (m, 1H), 3.97-3.95 (m, 1H), 3.70-3.65 (m, 1H), 3.58-3.52 (m, 1H), 3.46 (br s, 6H), 3.10-3.03 (m, 4H), 1.19 (t, *J*=14, 6H) ; ^{13}C NMR (100 MHz, $CDCl_3$) δ 154.3, 151.7, 149.9, 138.6, 119.8, 87.8, 85.7, 73.5, 70.5, 61.5, 45.3, 38.0, 8.40 ppm. HRMS m/z calculated for $C_{12}H_{17}N_5O_4$ (M^++H), 296.1353, observed m/z = 296.1353.

5'-O-(4,4'-dimethoxytrityl)- 6-*N,N*-dimethyladenosine (S_A2)[1]

Nucleoside **S_A1** (2.5 g, 7.5 mmol) was azeotropically dried over pyridine (15 mL) and redissolved in pyridine (50 mL). 4,4'-dimethoxytriphenylmethyl chloride (2 g, 5.9 mmol) was added at once at 0 °C and the solution was warmed to rt over 12h. The solution was partitioned in aqueous $NaHCO_3$ (20 %) and ethyl acetate (10 mL, 3x) followed by washing and drying of the organics with brine and sodium sulfate. Purification by flash column chromatography (100 % DCM, then slow gradient to 4 % MeOH in DCM), **S_A2** was isolated as a white foam (2.5 g, 55 %, 4.2 mmol). Experimental spectra were shown to match literature. ^{1}H NMR (400 MHz, $CDCl_3$) δ 8.26 (s, 1H), 8.04 (s, 1H), 7.29-7.16 (m, 9H), 6.76-6.73 (m, 4H), 5.95 (d, *J*=6, 1H), 4.70-4.68 (m, 1H), 4.45-4.44 (m, 1H), 4.36-4.35 (m, 1H), 3.77, 3.76 (2S, 6H), 3.55 (br s, 6H), 3.46-3.43 (m, 1H), 3.24-3.21 (m, 1H) ppm.

2'-O-(t-butyldimethylsilyl)-5'-O-(4,4'-dimethoxytrityl)-6-*N,N*-dimethyladenosine (S_A3)

An RBF charged with nucleoside **S_A2** (1.6 g, 2.67 mmol) and $AgNO_3$ (0.9 g, 5.3 mmol) was dissolved in THF (45 mL) and pyridine (2.6 mL, 32.2 mmol). Tert-butyldimethylsilyl chloride (0.44 g, 2.9 mmol) was added at once after 30 min and allowed to stir for 18 h. Ethyl acetate (10 mL) was added with stirring for 3 min followed by filtration of the precipitate. The filtrate was partitioned over aqueous $NaHCO_3$ and ethyl acetate (10 mL, x1) followed washing and drying of the organics with brine and sodium sulfate respectively. Purification using flash column chromatography (100 % hexanes, then slow gradient to 60 % EtOAc in hexanes) yielded **S_A3** as a white foam (0.75 g, 40 %,1.05 mmol). IR (cm^{-1}): 3234, 2950, 2927, 2855, 2360, 2340, 1594, 1508; ^1H NMR (400 MHz, $CDCl_3$) δ 8.28 (s, 1H), 7.95 (s, 1H), 7.48-7.46 (m, 2H), 7.40-7.20 (m, 7H), 6.84-6.80 (m, 4H), 6.04 (d, *J*=5, 1H), 4.98-4.96 (m, 1H), 4.33-4.30 (m, 1H), 4.26-4.23 (m, 1H), 3.79 (s, 6H), 3.55-3.34 (m br, 8H), 2.72 (d, *J*=4, 1H), 0.85 (s, 9H), -0.0019 (s, 3H), -0.11 (s, 3H); ^{13}C NMR (100 MHz, $CDCl_3$) δ 158.7, 144.8, 135.93, 135.87, 130.2, 129.3, 128.3, 128.02, 128.00, 127.9, 127.0, 113.3, 88.1, 86.6, 84.1, 75.6, 71.6, 63.6, 55.4, 38.8, 25.7, 18.06, -4.79, -5.10 ppm. HRMS m/z calculated for $C_{39}H_{49}N_5O_6Si$ (M^++H), 712.3525, observed m/z = 712.3514.

2'-O-(t-butyldimethylsilyl)-3'-O-(2-ethylcyano-*N,N*-diisopropylphosphoramidite)-5'-O-(4,4'-dimethoxytrityl)-6-*N,N*-dimethyladenosine (S_A4)

Nucleoside **S_A3** was dissolved in DCM (1.2 mL) and DIPEA (0.44 mL, 2.5 mmol), followed by the addition of 2- cyanoethyl-*N,N*-diisopropylchloro phosphoramidite (0.14 mL, 0.63 mmol) and stirred at rt for 1.25 h. 2-cyanoethyl-*N,N*-diisopropylchloro phosphoramidite (0.05 mL, 2.3 mmol) was added with stirring for 10 min. The reaction was partitioned over 50% $NaHCO_3$ and DCM (2 mL, x3) followed by washing then drying of the organics with brine and sodium sulfate respectively. Purification by flash column chromatography (slow gradient to 33 % EtOAc in Hexanes) **S_A4** was isolated as a white foam (0.3 g, 0.33 mmol, 78 %). IR (cm^{-1}): 2962, 2928, 2856, 2359, 2340, 1593, 1508; ^1H NMR (300 MHz, $CDCl_3$) δ 8.24 (s, 1H), 8.21 (s, 1H), 7.95 (s, 1H), 7.91 (s, 1H), 7.48-7.44 (m, 4H), 7.37-7.17 (m, 14H), 6.82-6.78 (m, 8H), 6.03-5.97 (m, 2H), 5.09-5.03 (m, 2H), 4.40-4.30 (m, 4H), 4.02-3.23 (m, 38H), 1.32-0.96 (m, 28H), 0.78 (s, 9H), 0.77 (s, 9H), -0.022 (s, 3H), -0.061 (s, 3H), -0.18 (s, 3H), -0.20 (s, 3H); ^{31}P NMR (122 MHz, $CDCl_3$) δ 150.8, 148.9 ppm. HRMS m/z calculated for $C_{48}H_{66}N_7O_7PSi$ (M^++H), 912.4603, observed m/z = 912.4610.

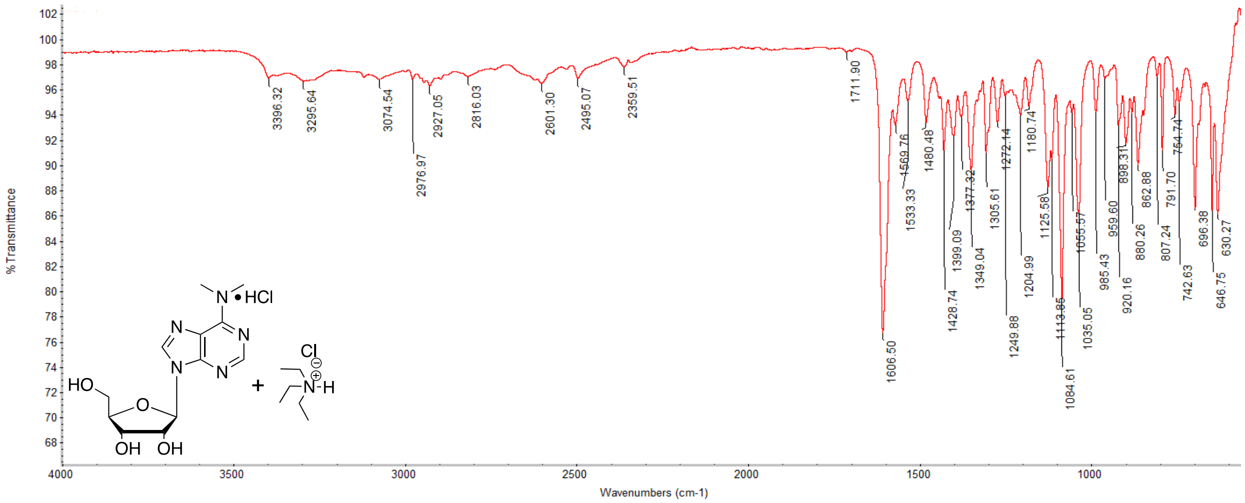

Figure S19. IR spectrum of **SA1**

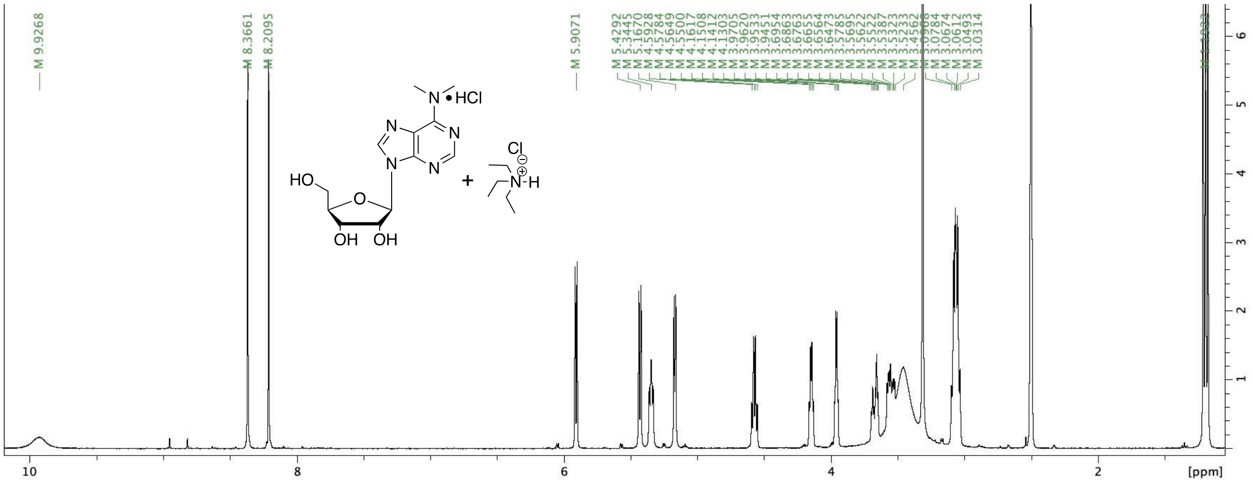

Figure S20. ^{1}H NMR of compound **SA1**

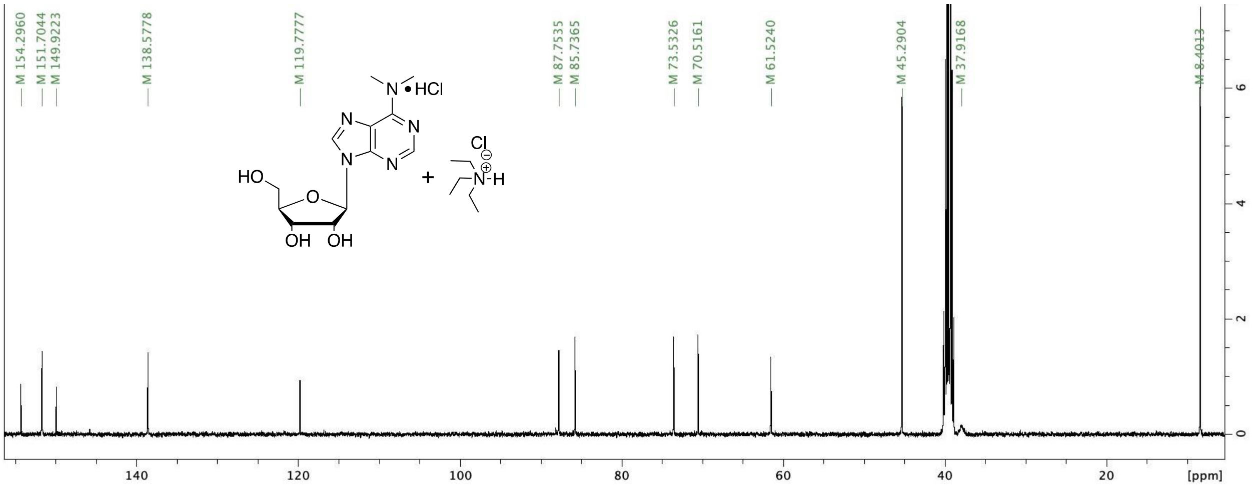

Figure S21. ^{13}C NMR of compound **SA1**

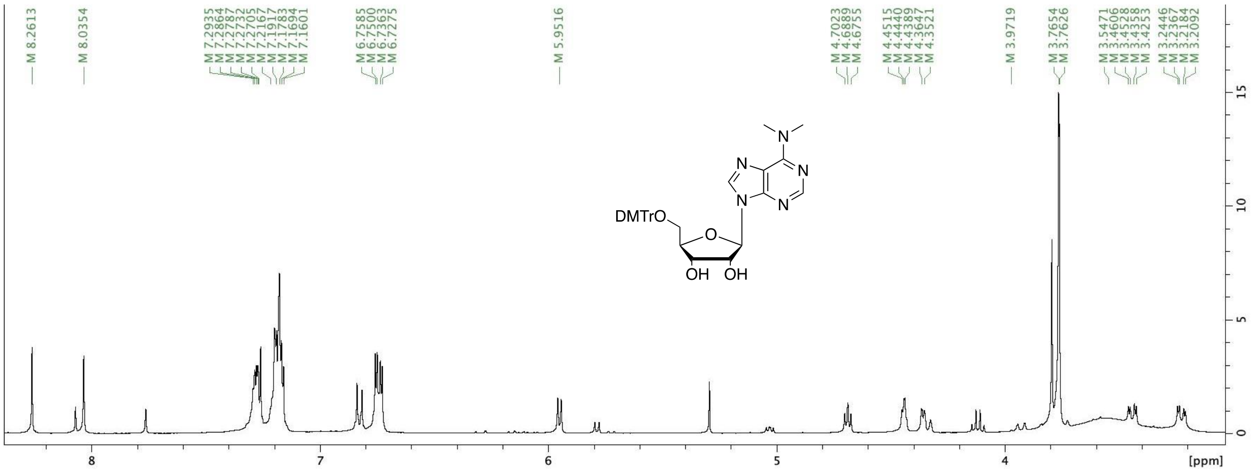

Figure S22. ^{1}H NMR of compound **SA2**

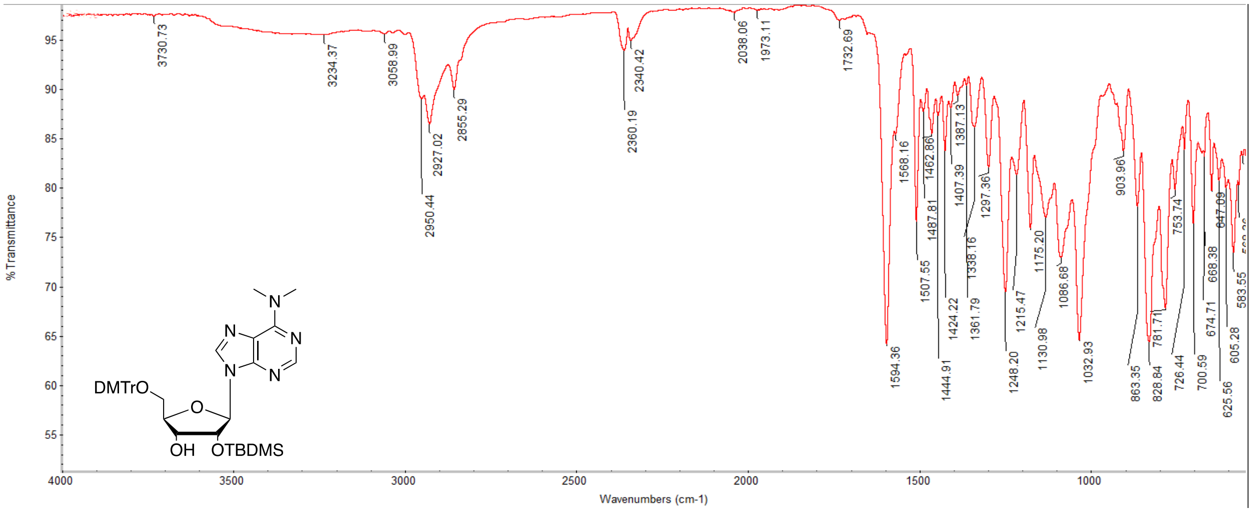

Figure S23. IR spectrum of **SA3**

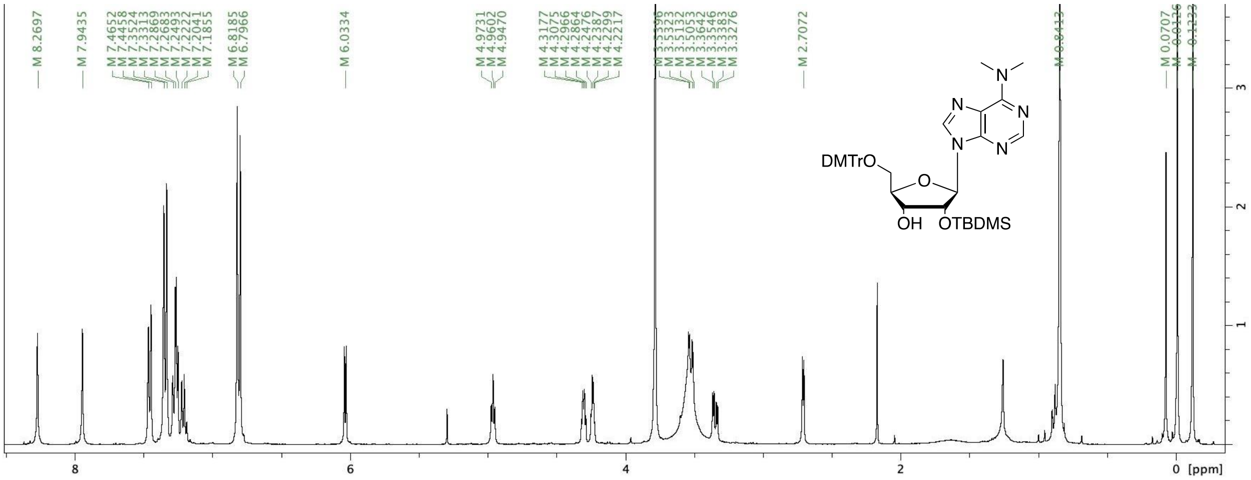

Figure S24. ^{1}H NMR of compound **SA3**

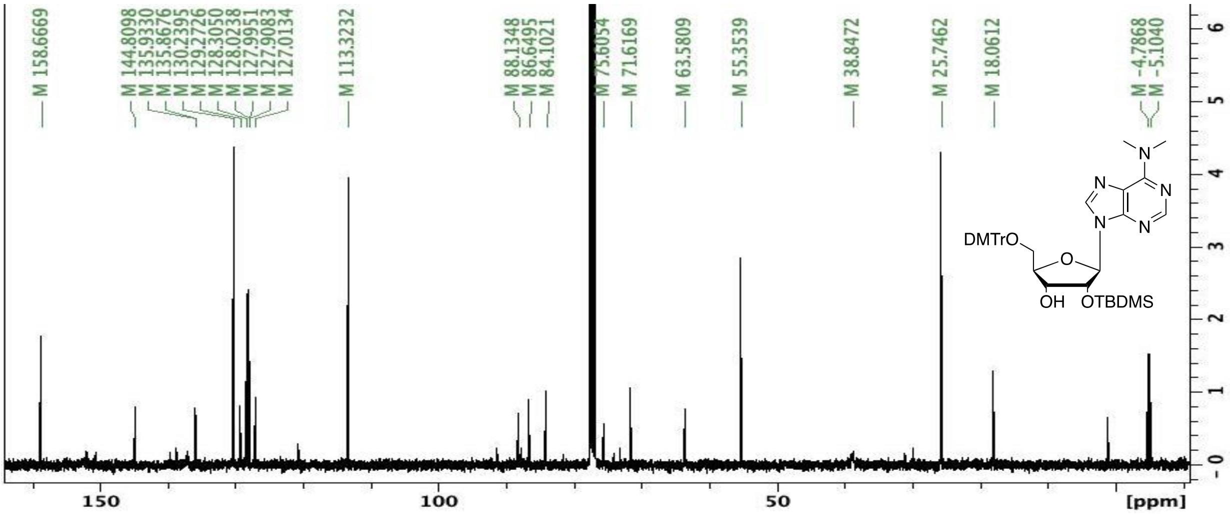

Figure S25. ^{13}C NMR of compound **SA3**

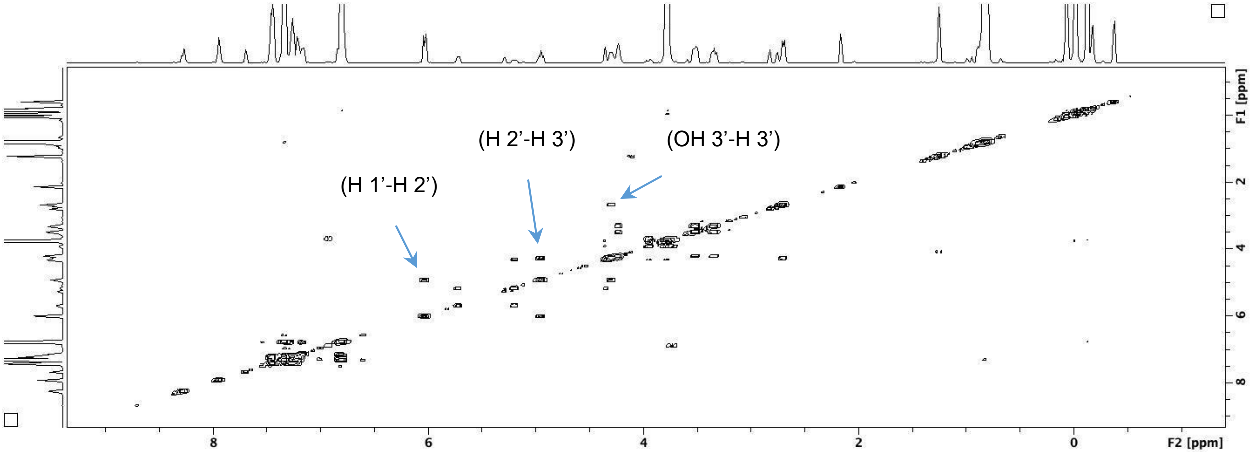

Figure S26. COSY NMR spectrum of **SA3**

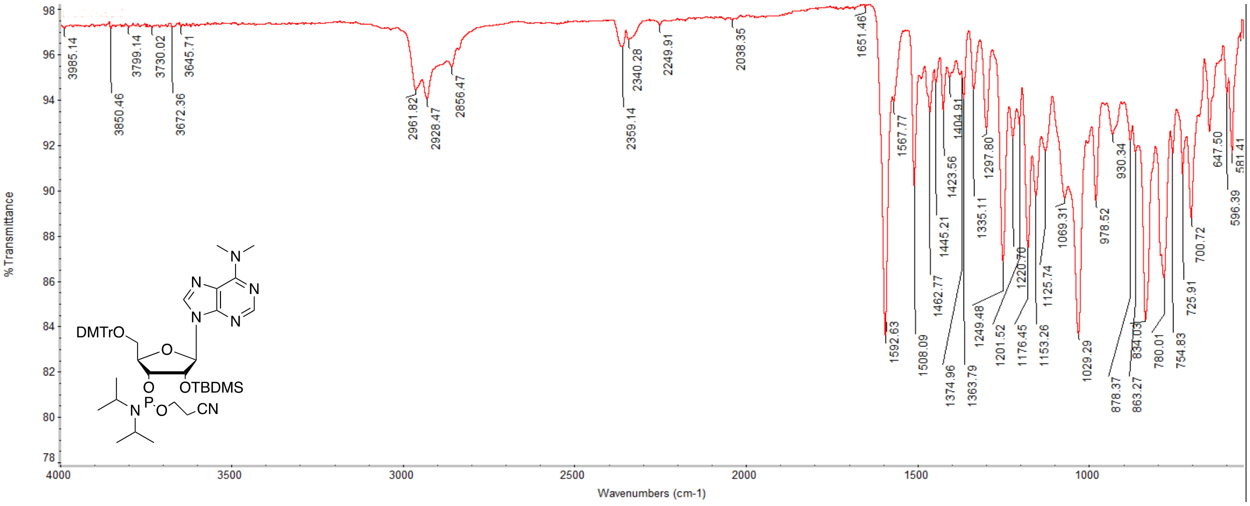

Figure S27. IR spectrum of **SA4**

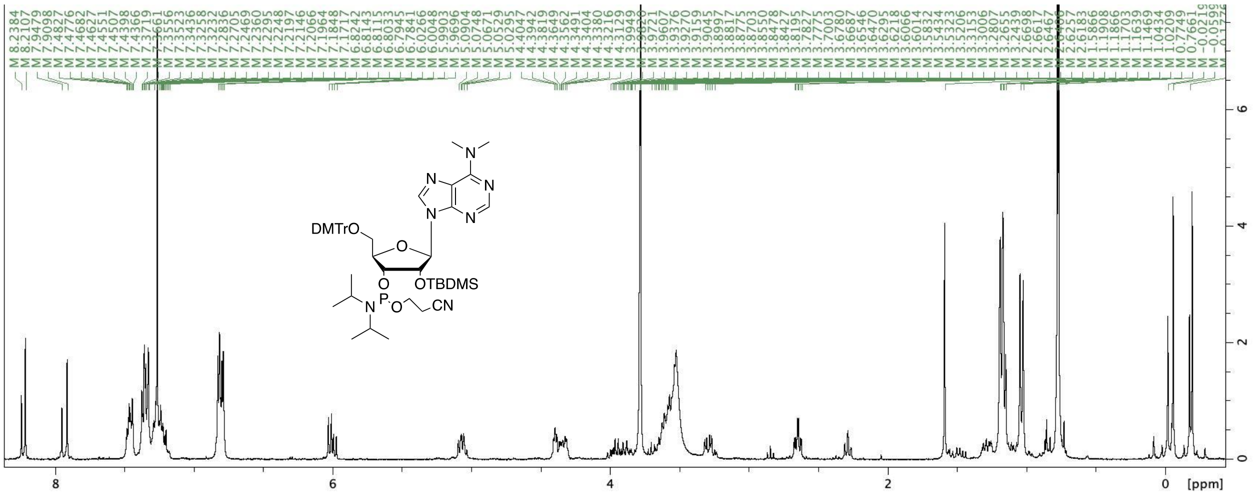

Figure S28. ^{1}H NMR of compound **S_A4**

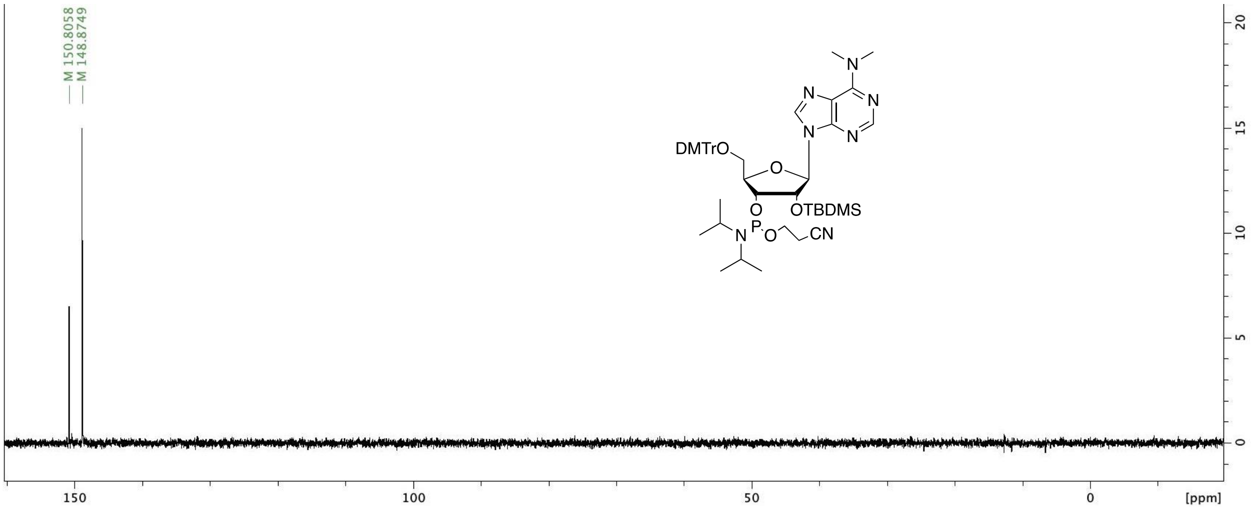

Figure S29. ^{31}P NMR of compound **S_A4**

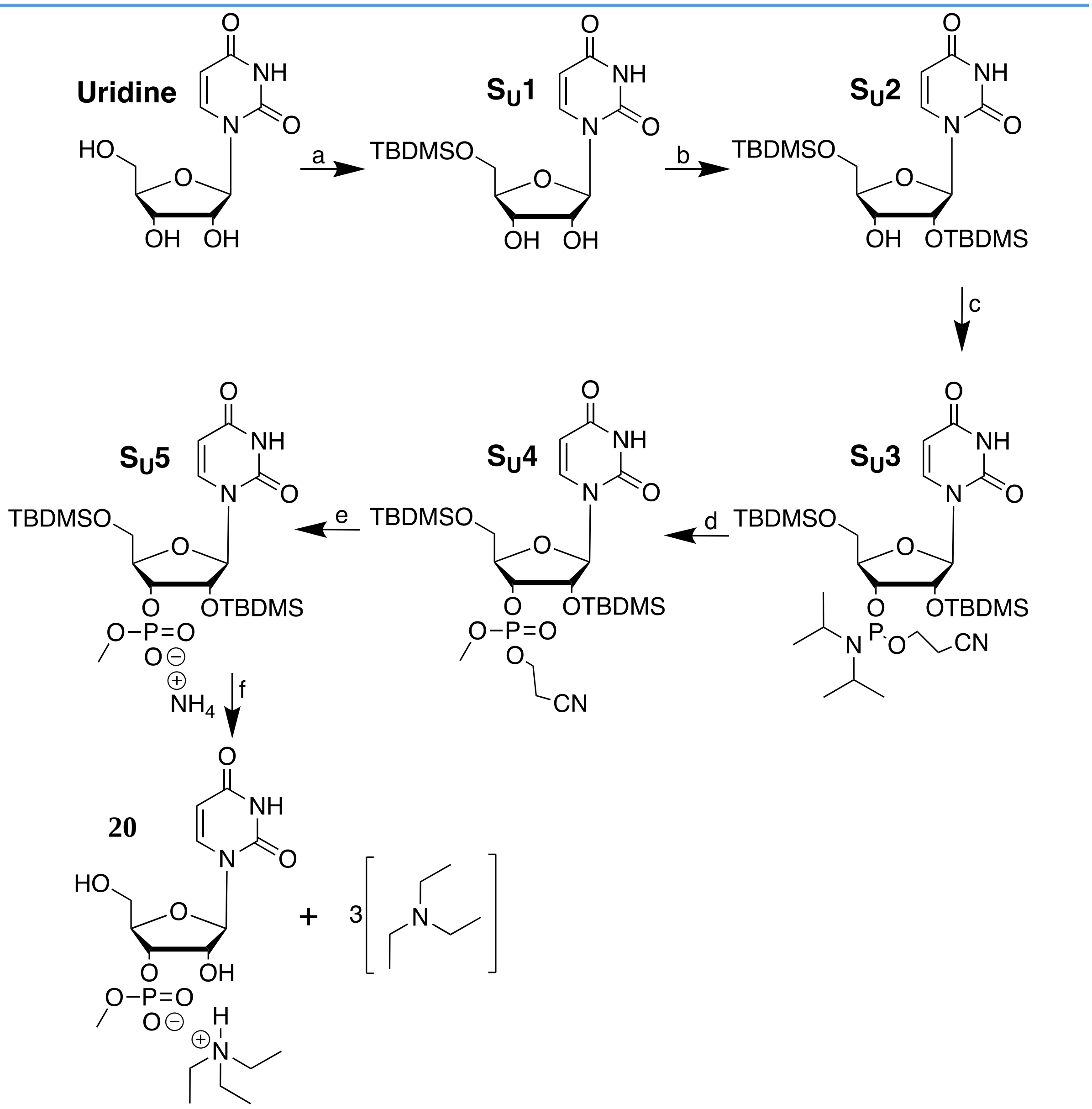

Scheme S3. *Synthesis of 3'-O-(methyl phosphate)-uridine* ***S*U*6***; (a) tert-butyldimethylsilyl chloride, pyridine, 0 °C → rt, 12h, **S**U**1**: 65 %; (b) tert-butyldimethylsilyl chloride, $AgNO_3$, pyridine, THF, rt, 13h, **S**U**2:** 57%; (c) 2-cyanoethyl-*N*,*N*-diisopropylchlorophos phoramidite, DIPEA, DCM, 1.25h, **S**U**3**: 87%; (d) 5-ethylthio)-1*H*-tetrazole, MeOH, CH_3CN, $(CH_3)_3COOH$, rt, 2h, **S**U**4**: 70%; (e) NH_4OH, MeOH, rt, 0.5 h, **S**U**5**: 87 %; (f) 3HF•TEA, THF, rt, 16h, **S**U**6**: 71 %.

5'-O-(t-butyldimethylsilyl)-uridine (SU1)[2]

Uridine (2g, 8.19 mmol) was dissolved in pyridine (45 mL) and cooled to 0 °C followed by the addition of tert-butyldimethylsilyl chloride (1.36 g, 9.0 mmol). The reaction was stirred with slow warming to rt for 12 h. The Solution was partitioned over 20 % aqueous $NaHCO_3$ and ethyl acetate (20 mL, x2). Organics washed with H_2O (10 mL, x2), followed by washing and drying over brine and sodium sulfate respectively. Purification by flash column chromatography (100 % DCM, then slow gradient to 5 % MeOH in DCM) to afford **S**U**1** as a white foam (1.9 g, 65 %, 5.3 mmol). Experimental spectra were shown to match literature. ^{1}H NMR (300 MHz, DMSO-d6)

δ 11.31 (s, 1H), 7.77 (d, *J*=8, 1H), 5.77 (d, *J*=5, 1H), 5.56 (d, *J*=8, 1H), 5.43 (d, *J*=5, 1H), 5.08 (d, *J*=5, 1H), 4.02-3.70 (m, 4H), 0.886 (s, 9H), 0.0759 (s, 6H) ppm.

5', 2'-O-bis-(t-butyldimethylsilyl)-uridine (SU2)[3]

An RBF charged with Nucleoside **SU1** (1.74 g, 4.85 mmol) and $AgNO_3$ (1.6 g, 9.5 mmol) was dissolved in THF (40 mL) and pyridine (2.3 mL, 28.5), followed by addition of tert-butyldimethylsilyl chloride (0.88 g, 5.8 mmol) with stirring for 12 h. Ethyl acetate was added and stirred for 5 min. The precipitate was filtered out and the filtrate was partitioned with 20 % $NaHCO_3$ and ethyl acetate (10 mL, x3). The organics were washed then dried with brine and sodium sulfate respectively. Purification by flash column chromatography (100 % hexanes x3, then slow gradient to 30% EtOAc in hexanes) **SU2** was isolated as a white foam (1.3 g, 57 %, 2.75 mmol). Experimental spectra were shown to match literature. ^{1}H NMR (300 MHz, DMSO-d6) δ 11.35 (s, 1H), 7.80 (d, *J*=8, 1H), 5.81 (d, *J*=5, 1H), 5.59 (d, *J*=8), 5.06 (d, *J*=5, 1H), 4.11-4.08 (m, 1H), 3.95-3.74 (m, 4H), 0.900 (s, 9H), 0.839 (s, 9H), 0.0914 (s, 6H), 0.0354 (s,3H), 0.0149 (s, 3H); ^{13}C NMR (75 MHz, DMSO-d6) δ 162.8, 150.4, 139.7, 101.6, 87.6, 84.5, 75.8, 69.6, 62.6, 25.7, 25.6, 18.0, 17.8, -4.87, -5.24, -5.63 ppm.

5', 2'-O-bis-(t-butyldimethylsilyl)-3'-O-[(2-ethylcyano-*N,N*-diisopropylphosphorami dite)-uridine (SU3)

Nucleoside **SU2** (0.89 g, 1.88 mmol) was dissolved in DCM (4 mL) and DIPEA (1.6 mL, 9.2 mmol), followed by the addition 2-cyanoethyl-*N,N*-diisopropylchloro phosphoramidite. The mixture was stirred for 1.5 h, then partitioned over 20 % $NaHCO_3$ and DCM (2 mL, 3x). Organics were washed then dried with brine and sodium sulfate respectively. Purification by flash column chromatography (100 % hexanes, then slow gradient to 40% EtOAc in hexanes) afforded **SU3** as a white foam (1.1 g, 87 %, 1.68 mmol). IR (cm^{-1}): 3165, 3058, 2958, 2929, 2885, 2857, 1682; ^{1}H NMR (300 MHz, $CDCl_3$) δ 8.50-8.33 (m, 2H), 7.99-7.90 (m, 2H), 6.02-5.96 (m, 2H), 5.70-5.67 (m, 2H), 4.32-4.01 (m, 6H), 4.01-3.56 (m, 8H), 2.67-2.57 (m, 4H), 1.63-0.66 (m, 64H), 0.15-0.035 (m, 24H); ^{31}P NMR (122 MHz, $CDCl_3$) δ 150.38, 149.33 ppm. HRMS m/z calculated for $C_{30}H_{57}N_4O_7PSi_2$ (M^++H), 673.3576, observed m/z = 673.3587.

5', 2'-O-bis-(t-butyldimethylsilyl)-uridine-3'-O-(2-ethylcyano-methylphosphate) (SU4)

Compound **SU3** (0.41 g, 0.61 mmol) was dissolved in dry acetonitrile (5 mL) and methanol (0.2 mL, 4.95 mmol) followed by the addition of a 0.25 M solution of 5-ethylthio-1*H*-tetrazole (0.12 g, 0.9 mmol) dissolved in acetonitrile. The solution was stirred for 1 h, after which tertbutyl hydro peroxide (0.3 mL, 2.93 mmol) was added with stirring for 1 h. The Organic residue was concentrated under reduced pressure and purified via flash column chromatography (slow gradient to 50 % EtOAc in hexanes, then 100 % EtOAc) to give **SU4** as a white foam (0.26 g, 70 %, 0.43 mmol). IR (cm^{-1}): 3179, 3069, 2954, 2929, 2857,1692, 1634; ^{1}H NMR (400 MHz, $CDCl_3$) δ 8.98 (m, 2H), 7.87-7.84 (m, 2H), 6.08-6.02 (m, 2H), 5.74-5.71 (m, 2H), 5.76-4.67 (m, 2H), 4.37-4.24 (m, 4H), 3.97-3.78 (m, 10H), 2.81-2.77 (m, 4H), 0.93 (m, 18H), 0.864-0.860 (m, 18H), 0.13-0.031 (m, 24H); ^{31}P NMR (122 MHz, $CDCl_3$) δ -1.32, -1.40 ppm. HRMS m/z calculated for $C_{25}H_{46}N_3O_9PSi_2$ (M^++H), 620.2583, observed m/z = 620.2593.

5', 2'-O-bis-(t-butyldimethylsilyl)-uridine-3'-O-methylphosphate ammonium salt (SU5)

Nucleoside **S$_U$4** (0.24 g, 0.496 mmol) was dissolved in methanol (3mL) followed by addition of NH_4OH (40%) with stirring for 0.5 h. Organic residues were concentrated under reduced pressure without further purification to afford **S$_U$5** as a white foam (0.2 g, 87 %, 0.34 mmol). IR (cm^{-1}): 2952, 2929, 2884, 2857, 2360, 2341, 1692, 1634; ^{1}H NMR (400 MHz, DMSO-d6) δ 7.74 (d, *J*=8, 1H), 7.33 (br, 1H), 5.87 (d, *J*=6, 1H), 5.67 (d, *J*=8, 1H), 4.27-4.24 (m, 2H), 4.12-4.09 (m, 1H), 3.84-3.81 (m, 1H), 3.74-3.71 (m, 1H), 3.35-3.32 (m, 3H), 0.91 (s, 9H), 0.80 (s, 9H), 0.099 (s, 6H), 0.027 (s, 3H), -0.014 (s, 3H); ^{13}C NMR (100 MHz, DMSO-d6) δ 162.8, 150.6, 139.3, 102.0, 86.5, 84.6, 75.1, 73.3, 63.3, 51.6, 25.8, 25.5, 18.0, 17.8, -4.85, -5.55, -5.64, -5.67; ^{31}P NMR (122 MHz, DMSO-d6) δ -0.52 ppm. HRMS m/z calculated for $C_{22}H_{42}N_2O_9PSi_2$ (M^++H), 567.2317, observed m/z = 567.2320.

Uridine 3'-O-methylphosphate triethylammonium salt (20)[3]

To a flask containing a solution of **S$_U$5** (0.2 g, 0.34 mmol) and THF (7 mL), triethylamine trihydrofluoride (0.45 mL, 2.74 mmol) was added at once with stirring for 12 h. Organic residues were concentrated under reduced pressure and purified via flash column chromatography (100 % DCM, then slow gradient to 45 % MeOH in DCM) to isolate **20** as a white foam (0.18 g, 71%, 0.24 mmol). Experimental spectra were shown to match literature. ^{1}H NMR (400 MHz, D_2O) δ 7.83 (d, *J*=8, 1H), 5.88 (d, *J*=5, 1H), 5.82 (d *J*=8, 1H), 4.48 (m, 1H), 4.36 (m, 1H), 4.22 (m, 1H), 3.83-3.72 (m, 2H), 3.56-3.53 (m, 3H), 3.12 (m, 18H), 1.20 (m, 27H); ^{13}C NMR (100 MHz, D_2O) δ 165.9, 151.6, 141.7, 102.4, 88.5, 83.8, 73.3, 72.8, 60.6, 53.0, 46.5, 8.20; ^{31}P NMR (162 MHz, D_2O) δ 0.88 ppm. HRMS m/z calculated for $C_{10}H_{14}N_2O_9P^-$ (M^++H), 339.0588, observed m/z = 339.0565.

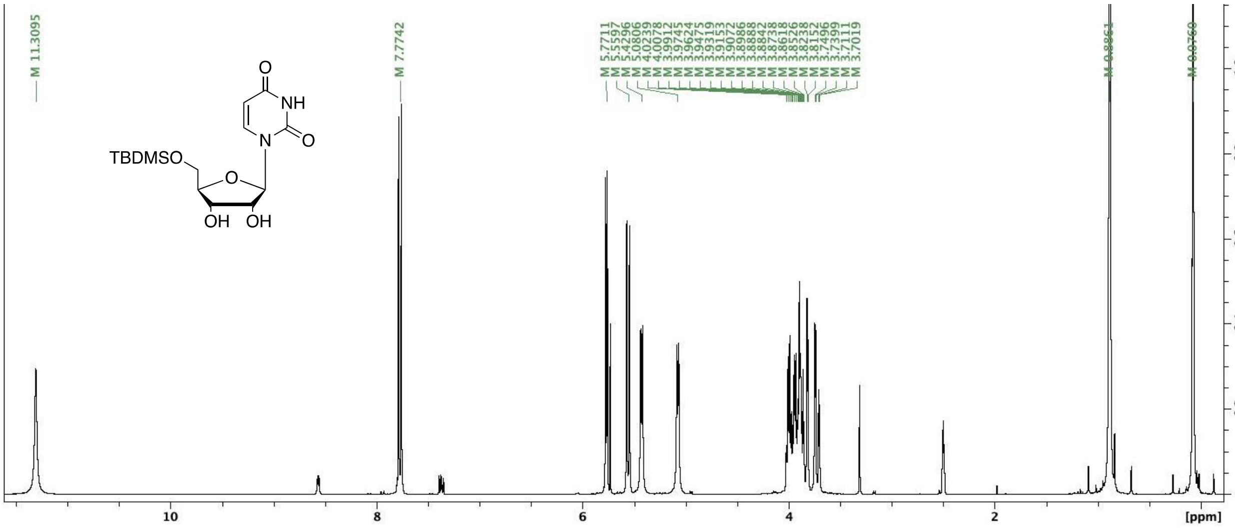

Figure S30. ^{1}H NMR of compound **S**U**1**

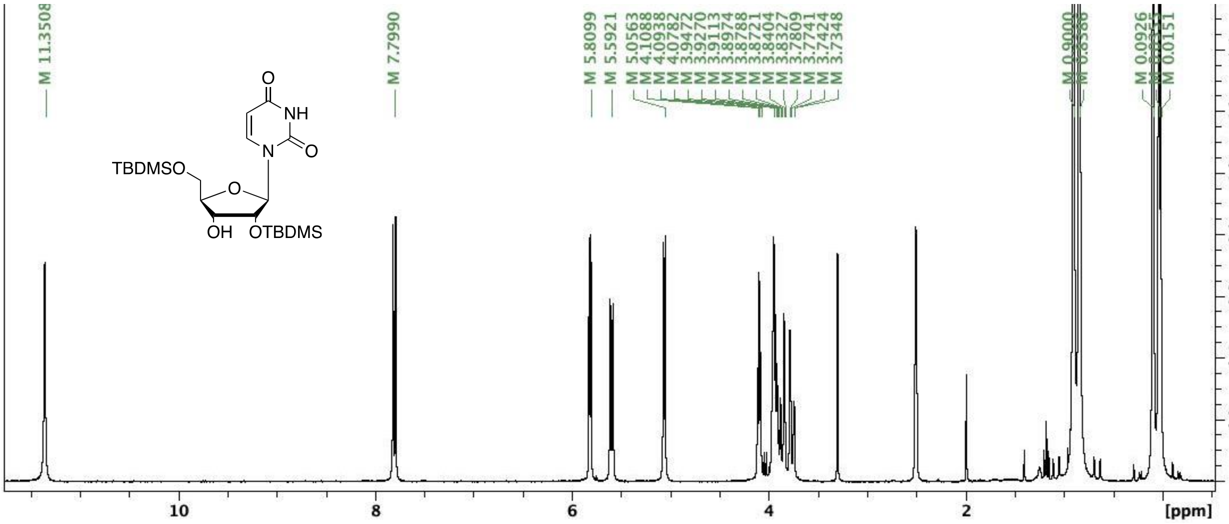

Figure S31. ^{1}H NMR of compound **S**U**2**

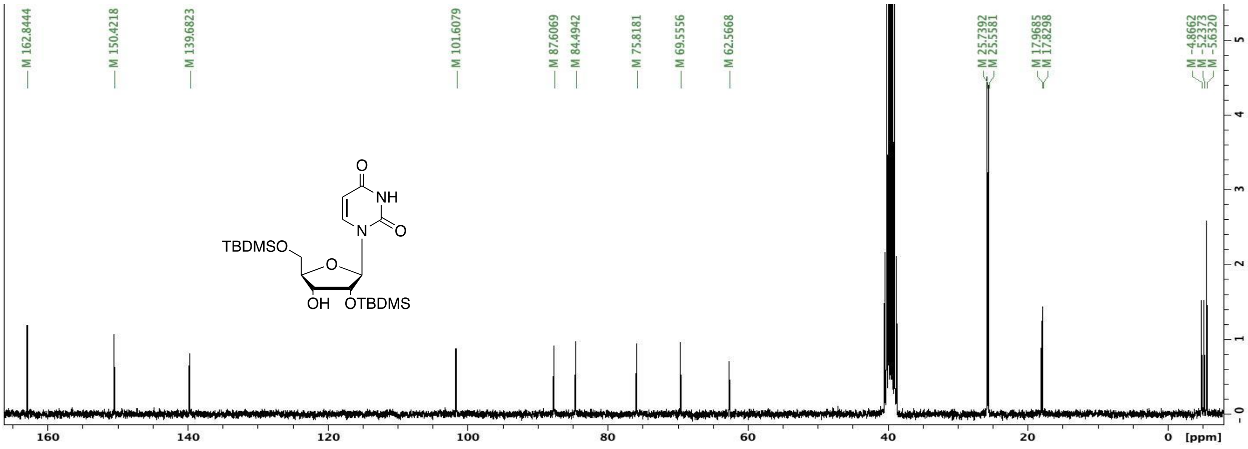

Figure S32. ^{13}C NMR of compound **S**U**2**

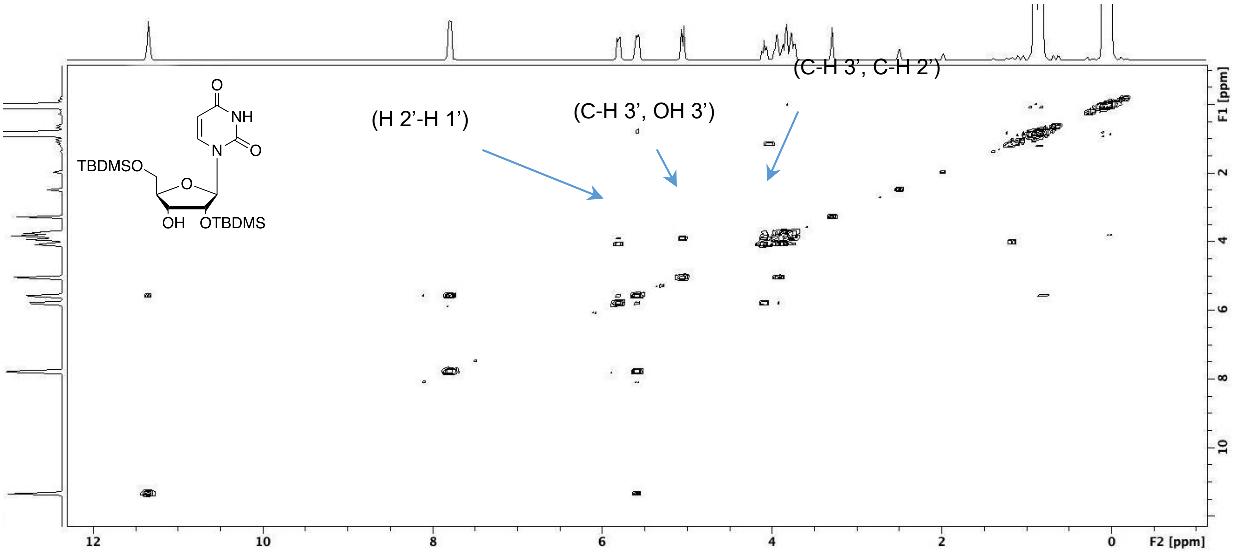

Figure S33. COSEY NMR spectrum of **S**U**2**

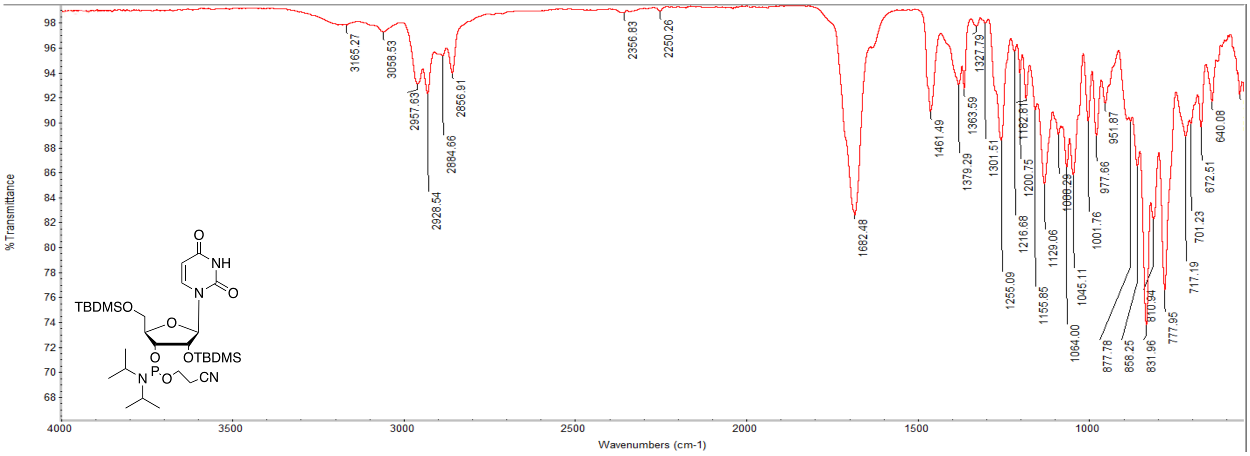

Figure S34. IR spectrum of **S**U**3**

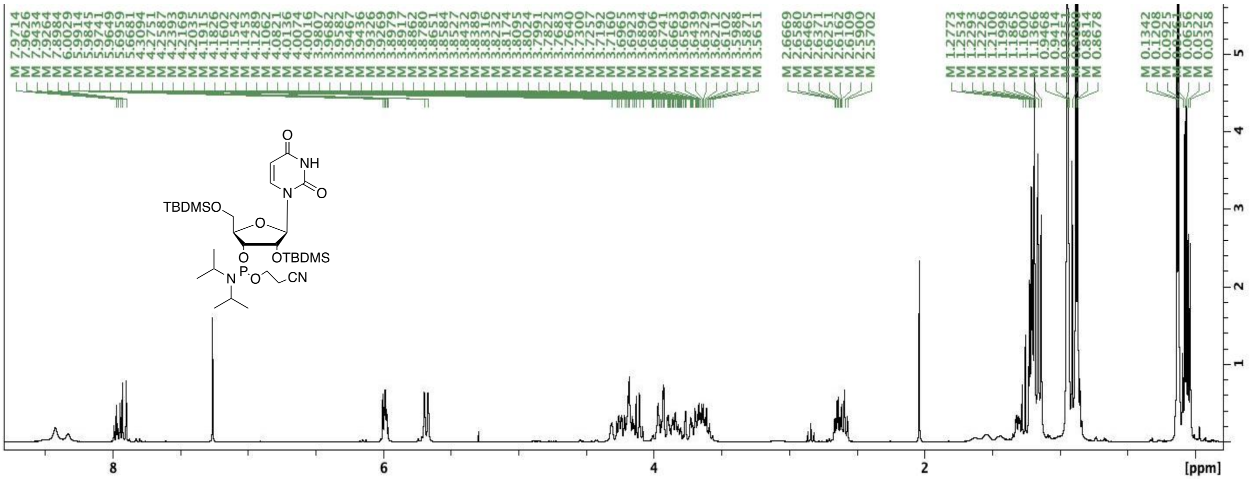

Figure S35. ^{1}H NMR of compound **S**U**3**

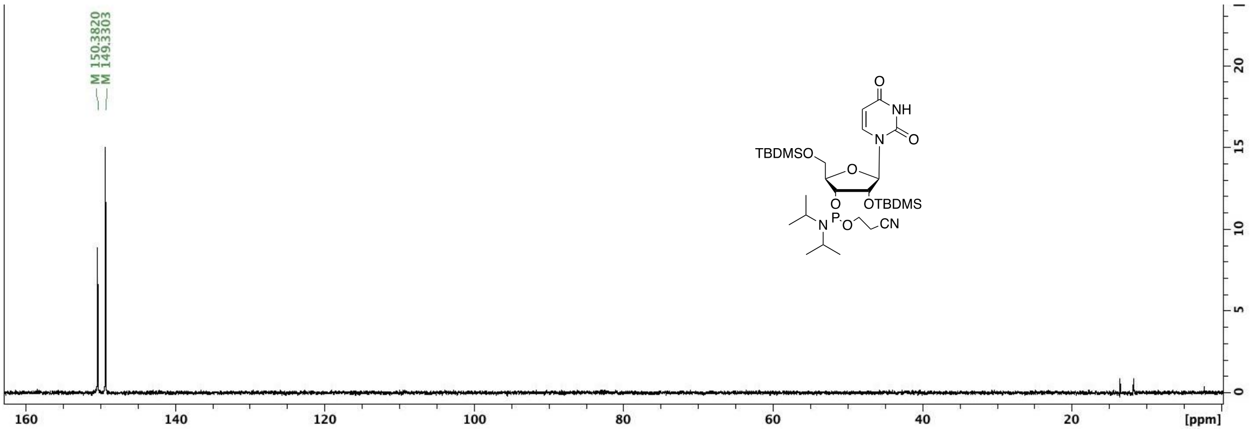

Figure S36. ^{31}P NMR of compound **SU3**

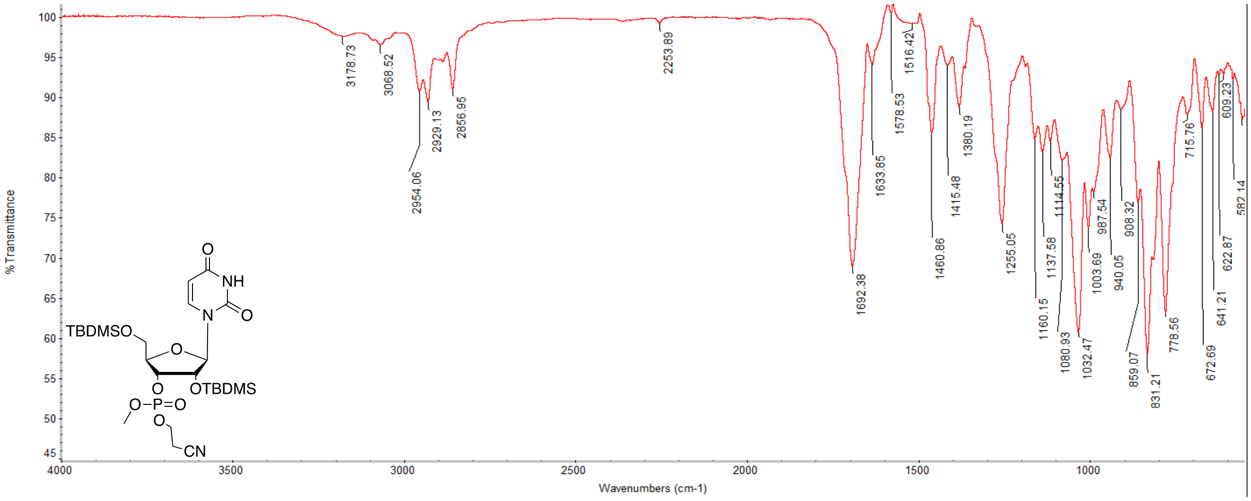

Figure S37. IR spectrum of **SU4**

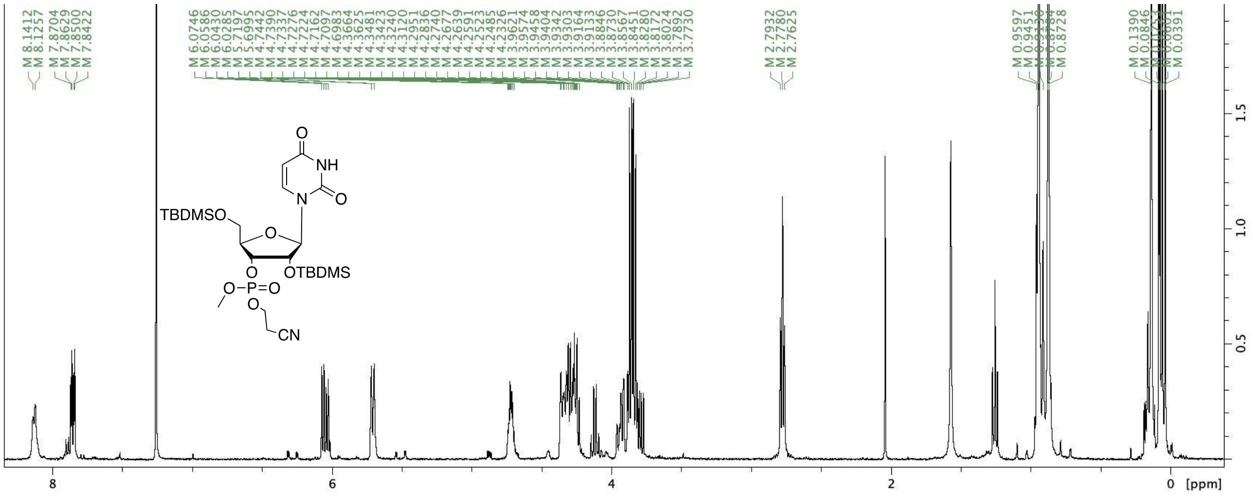

Figure S38. ^{1}H NMR of compound **SU4**

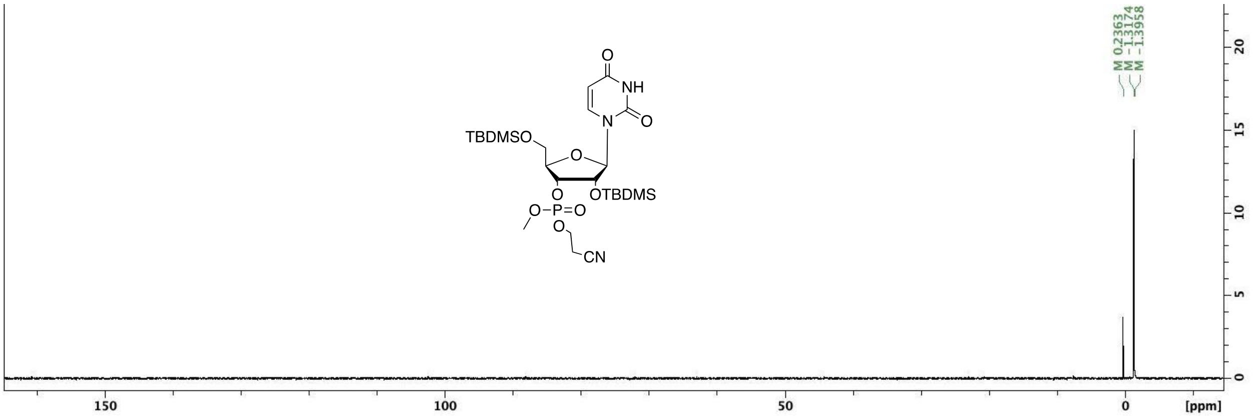

Figure S39. ^{31}P NMR of compound **SU4**

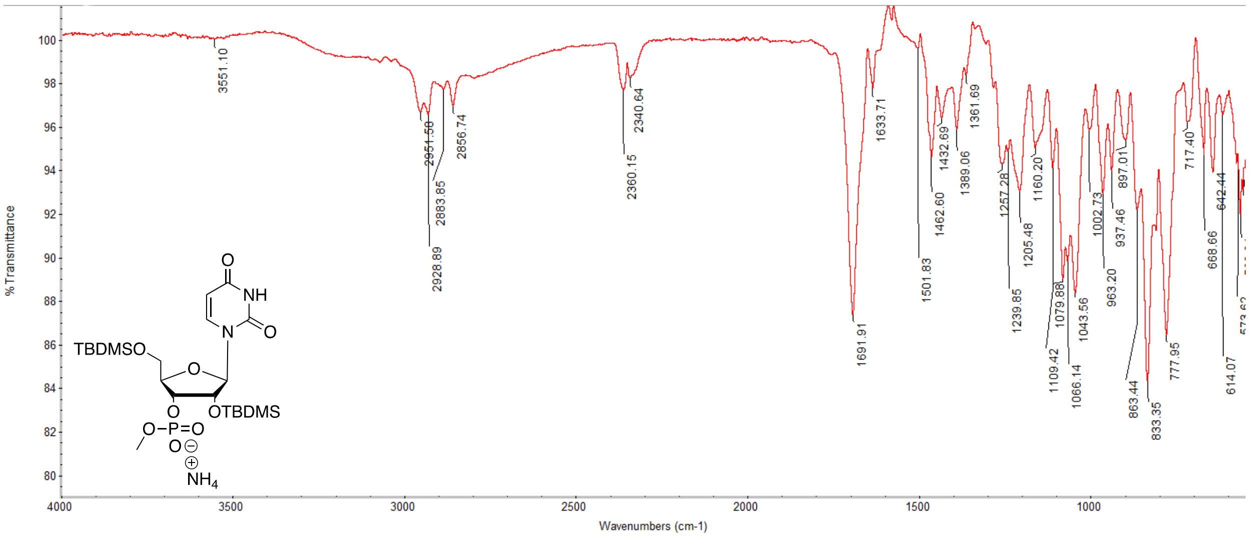

Figure S40. IR spectrum of **SU5**

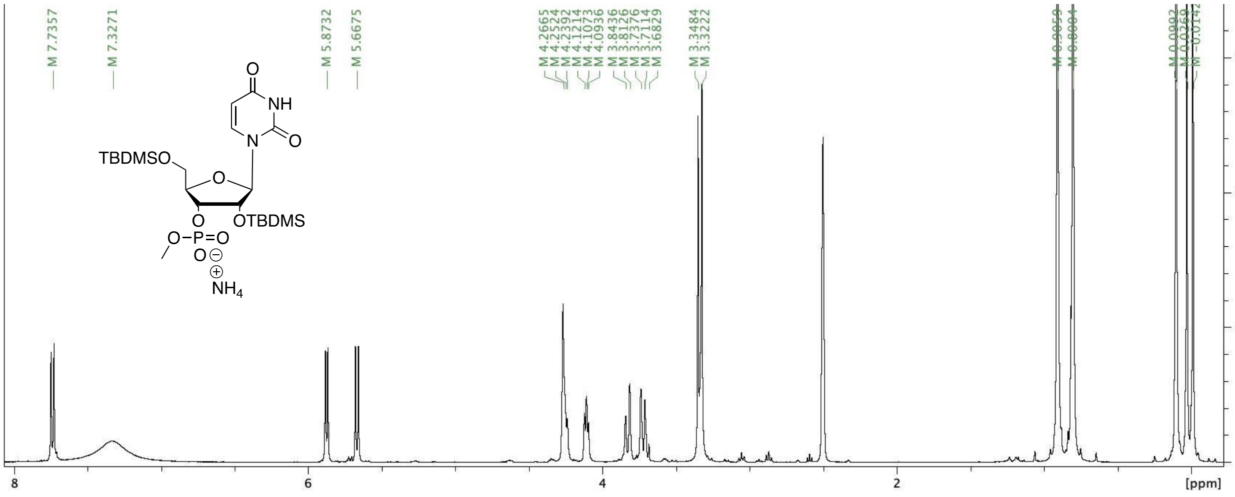

Figure S41. ^{1}H NMR of compound **SU5**

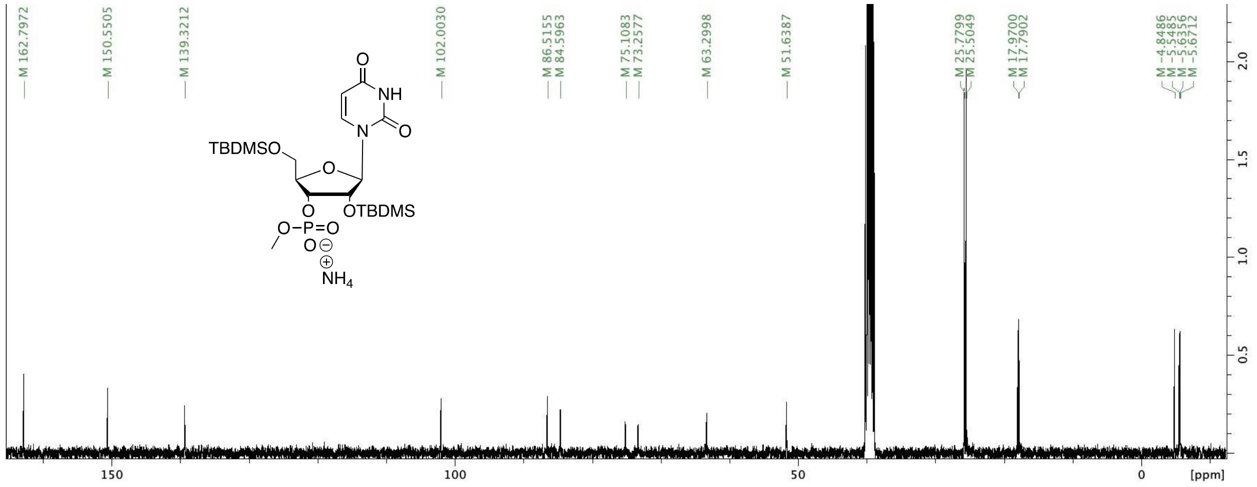

Figure S42. ^{13}C NMR of compound **S_U5**

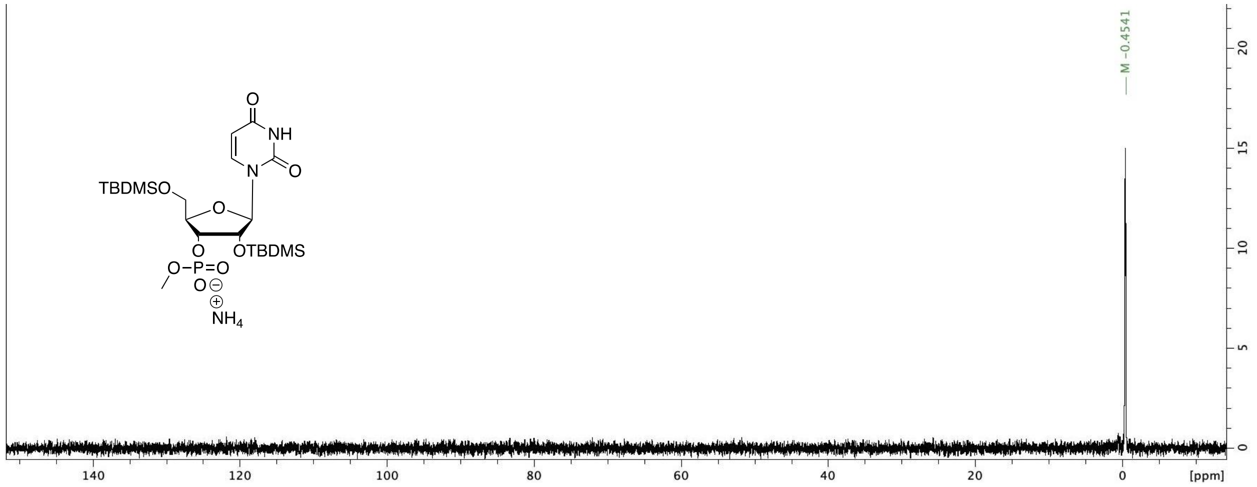

Figure S43. ^{31}P NMR of compound **SU5**

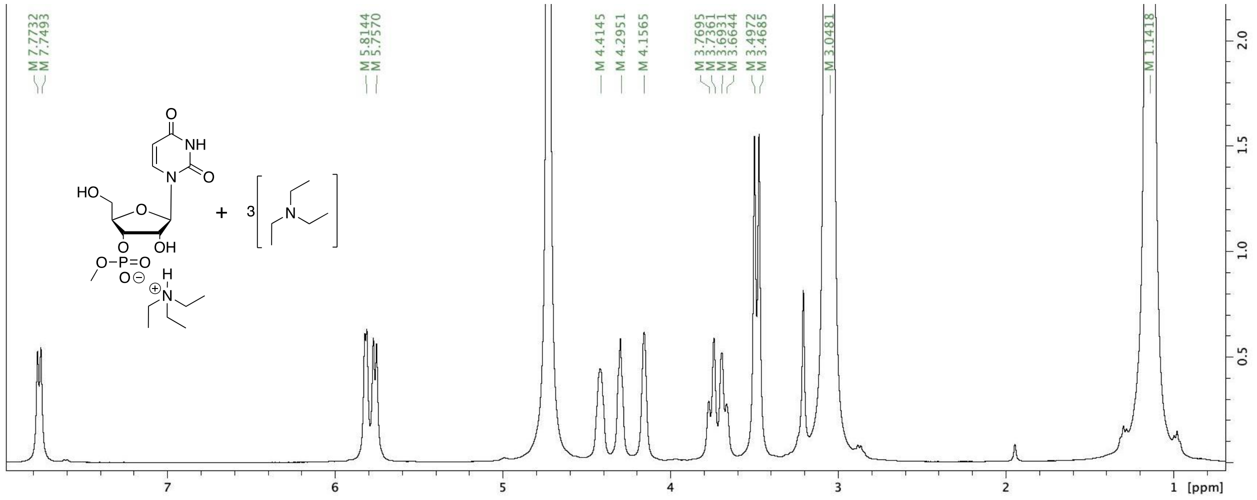

Figure S44. ^{1}H NMR of compound **SU6**

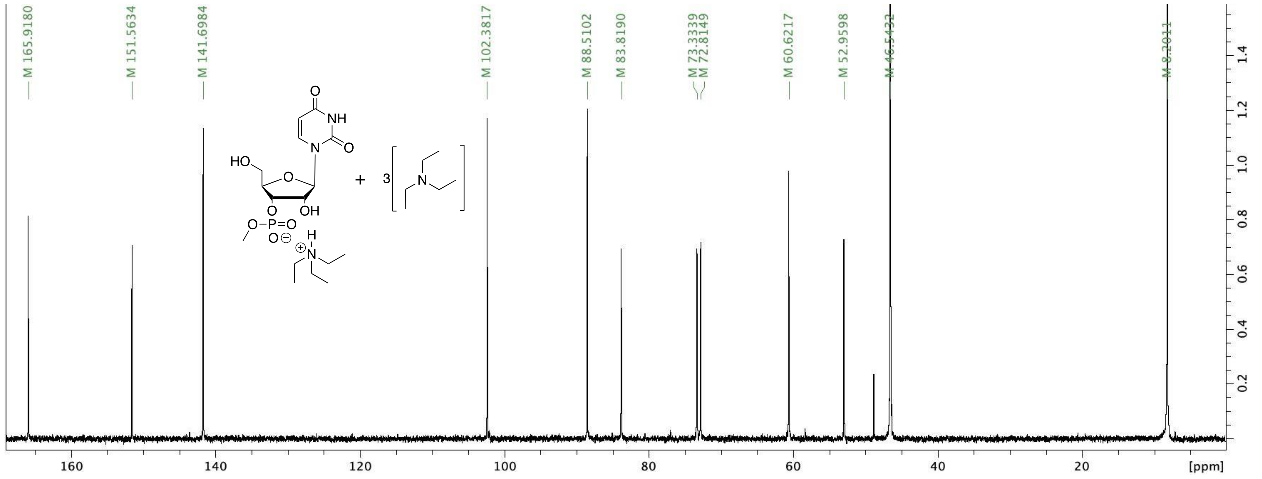

Figure S45. ^{13}C NMR of compound **S$_U$6**

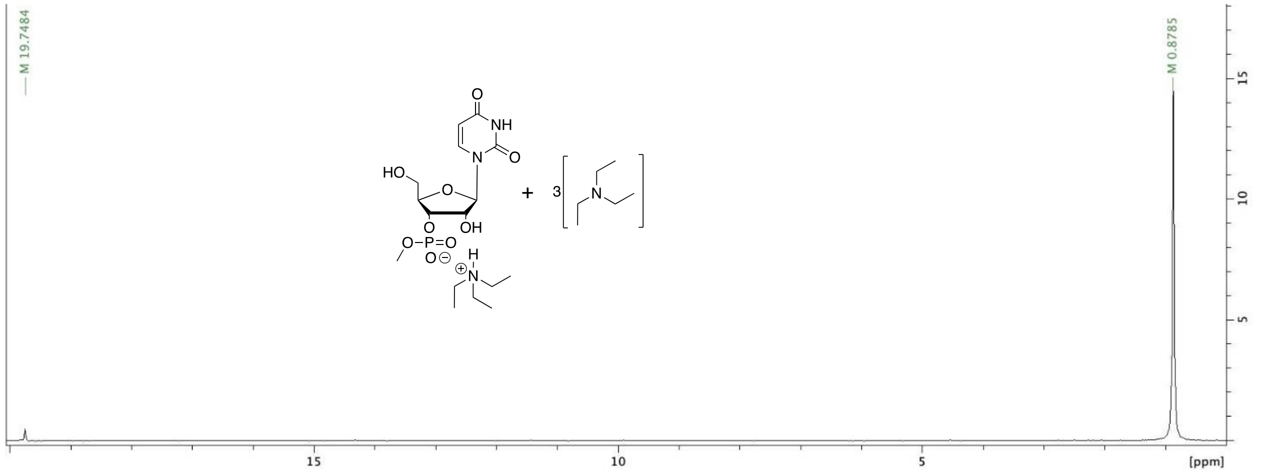

Figure S46. ^{31}P NMR of compound **S$_U$6**

Sequences used in this work

1| 5’-CAU GAA ACA AGG CUA AAA GU-3’
2| 5’-CAU GAA ACA A***G**G CUA AAA GU-3’
3| 5’-CAU GAA ACA A***G*****G** CUA AAA GU-3’
4| 5’-***G**GC UAA AAG U-3’
5| 5’-***G**CU AAA AGU-3’
6| 5’-CAU GAA ACA **m^{2,6}A**GG CUA AAA GU-3’
7| 5’-CAU GAA ACA A**m^{1}G**G CUA AAA GU-3’
8| 5’-CAU GAA ACA A^{Br}**G**G CUA AAA GU-3’
9| 5’-GAC GAA ACA GGG CUA AAG AU-3’
10| 5’-GAC GAA ACA ***G**GG CUA AAG AU-3’
11| 5’-GAC ***G**AA ACA GGG CUA AAG AU-3’
12| 5’-GAC GAA ACA ***G*****G**G CUA AAG AU-3’
13| 5’-GAC GAA ACA ***G*****G*****G** CUA AAG AU-3’
14| 5’-GAC GAA ACA **m^{1}G**GG CUA AAG AU-3’
15| 5’-GAC GAA ACA **m^{1}Gm^{1}G**G CUA AAG AU-3’
16| 5’-GAC GAA **m^{2,6}A**CA GGG CUA AAG AU-3’
17| 5'-UGU CAA CUC CAG CAC CAC CUA CAC ACC UC-3'
18| 5'-UGU CAA CUC CA***G** CAC CAC CUA CAC ACC UC-3'
19| 5'-UGU CAA CUC C***G*G** CAC CAC CUA CAC ACC UC-3'

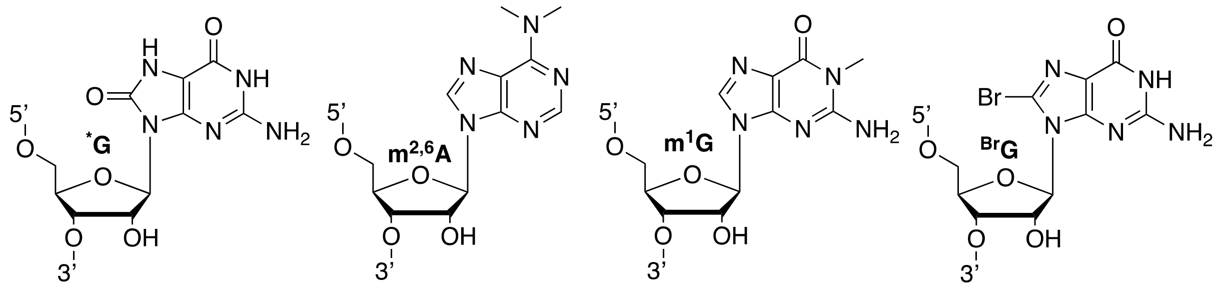

Figure S47. Oligonucleotide sequences used (top) **17-19** were synthesized and categorized as reported.[6] and chemical structures pertaining to the noncanonical nucleotides and their respective abbreviated names (bottom).

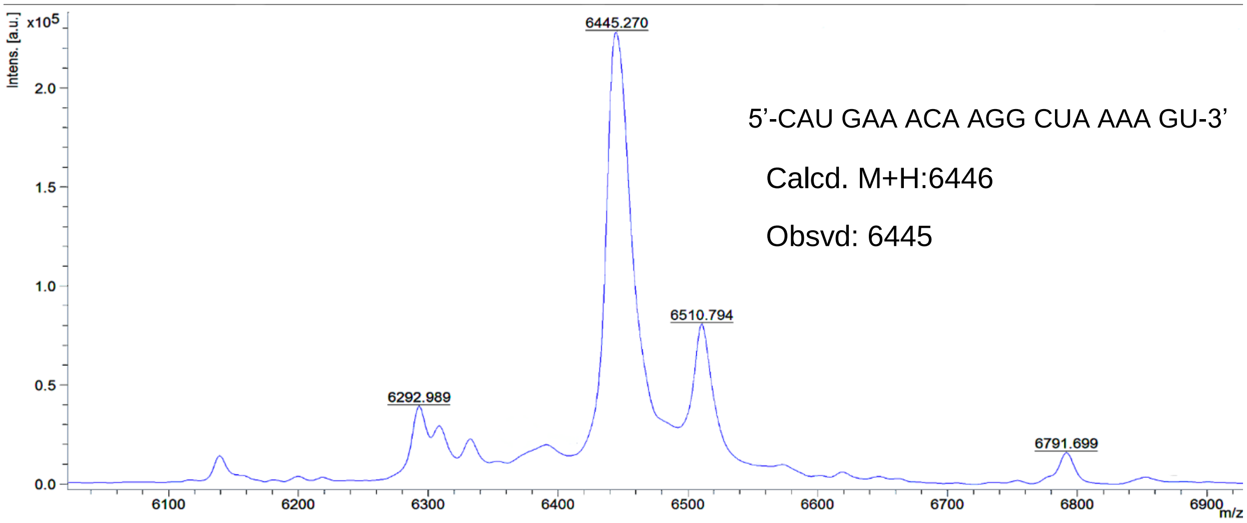

Figure S48. MALDI-TOF MS of **1**

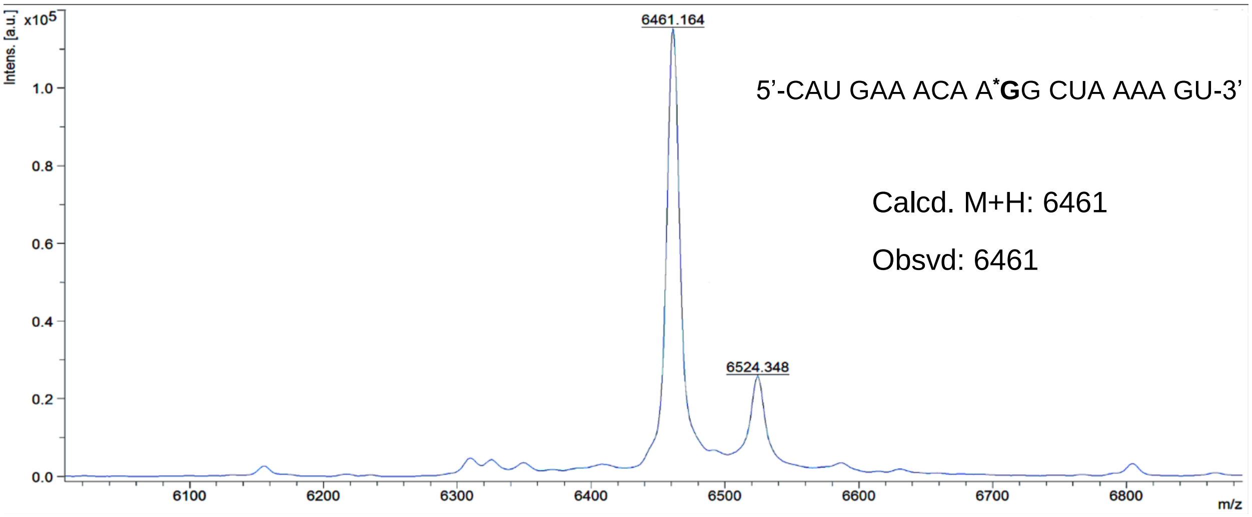

Figure S49. MALDI-TOF MS of **2**

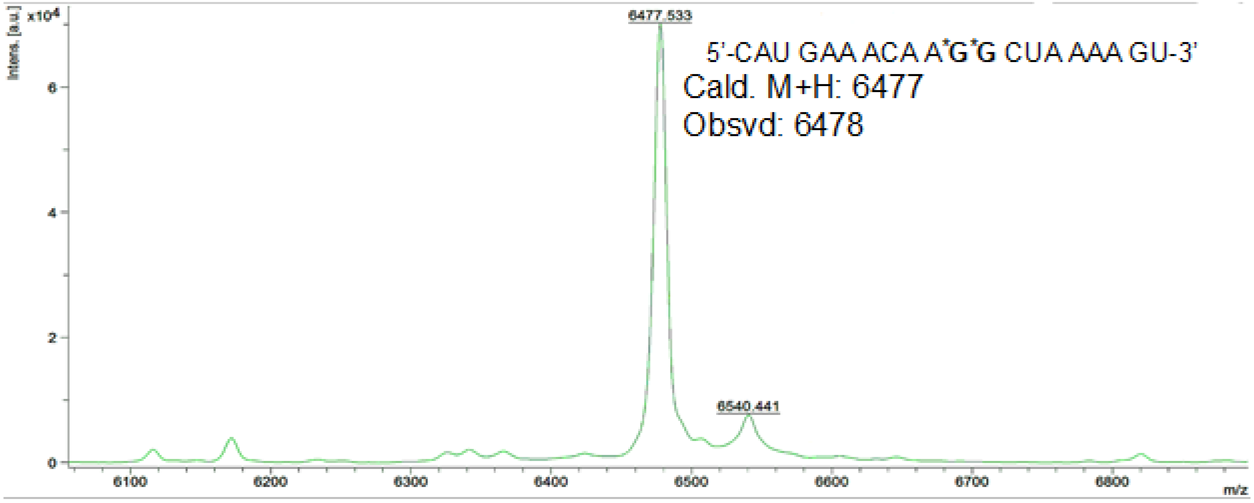

Figure S50. MALDI-TOF MS Spectrum of **3**

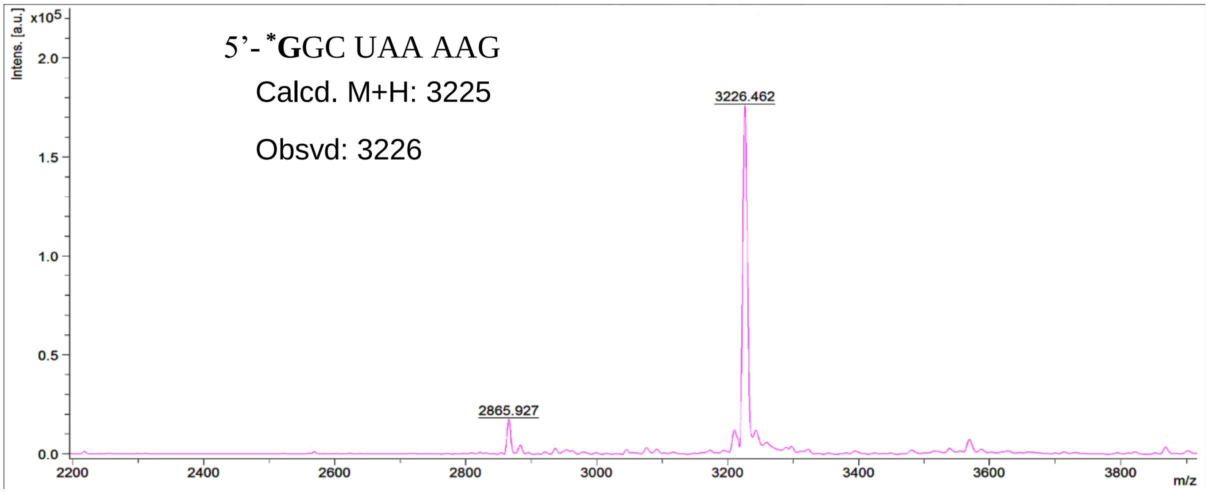

Figure S51. MALDI-TOF MS of **4**

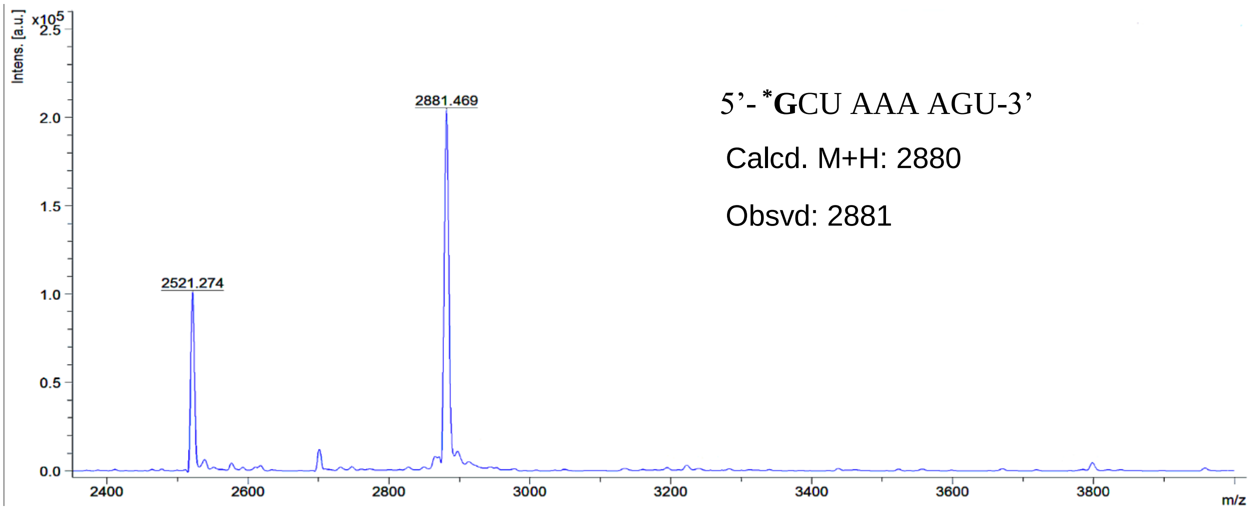

Figure S52. MALDI-TOF MS of **5**

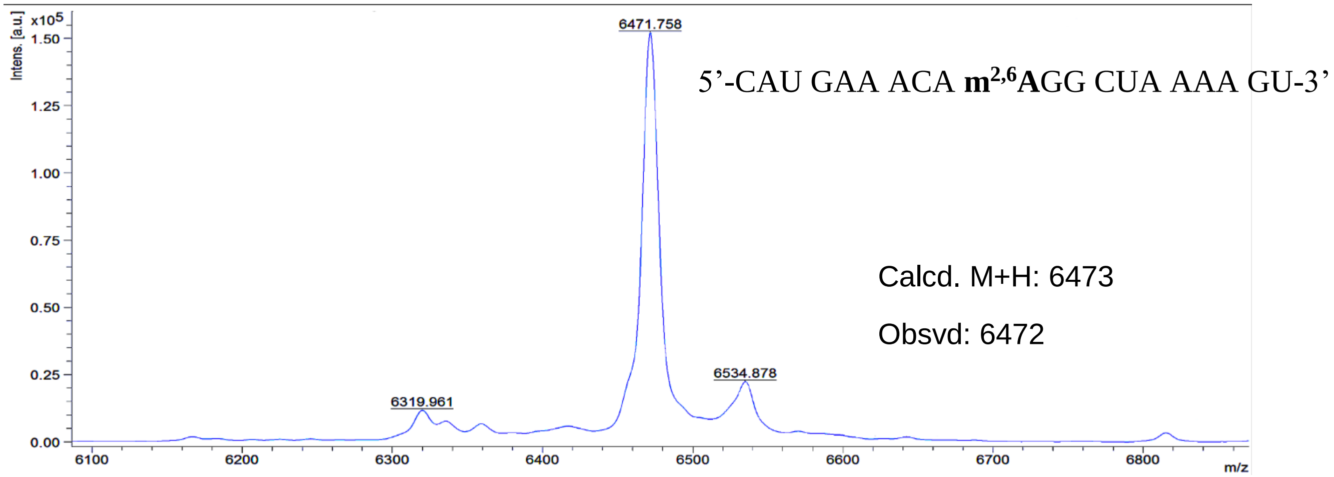

Figure S53. MALDI-TOF MS of **6**

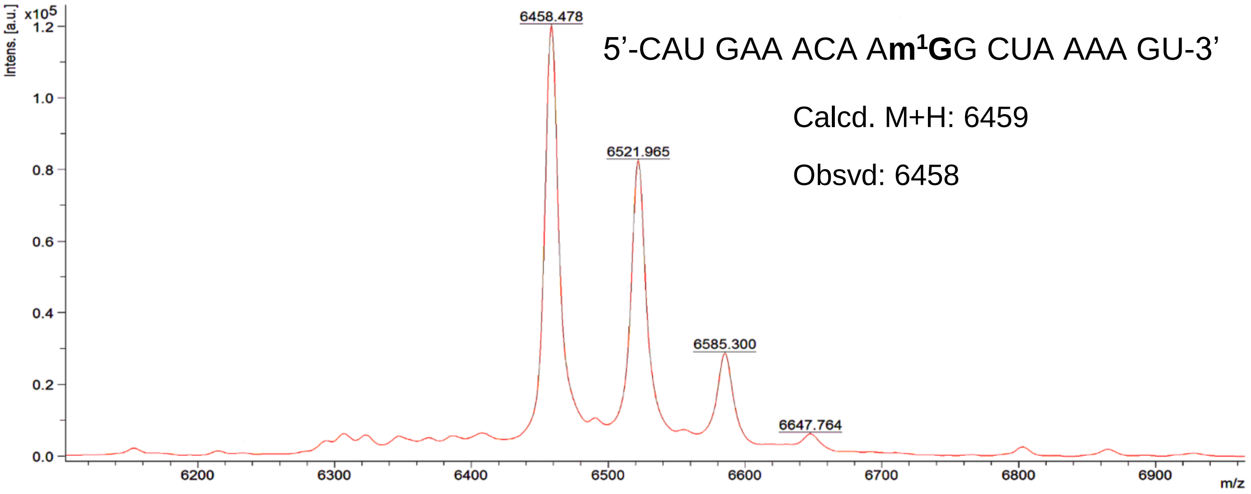

Figure S54. MALDI-TOF MS of **7**

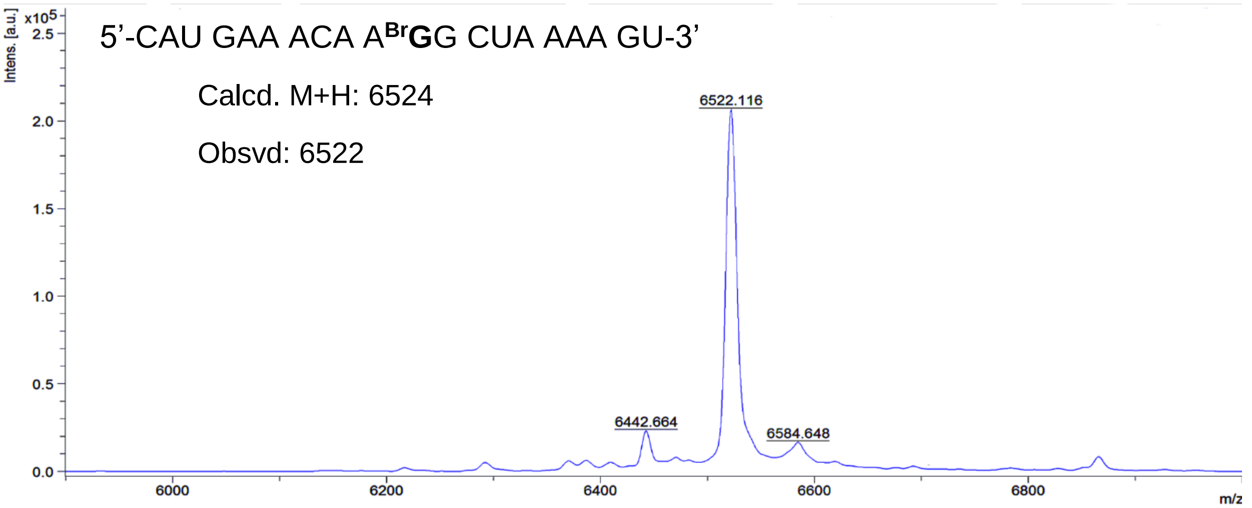

Figure S55. MALDI-TOF MS of **8**

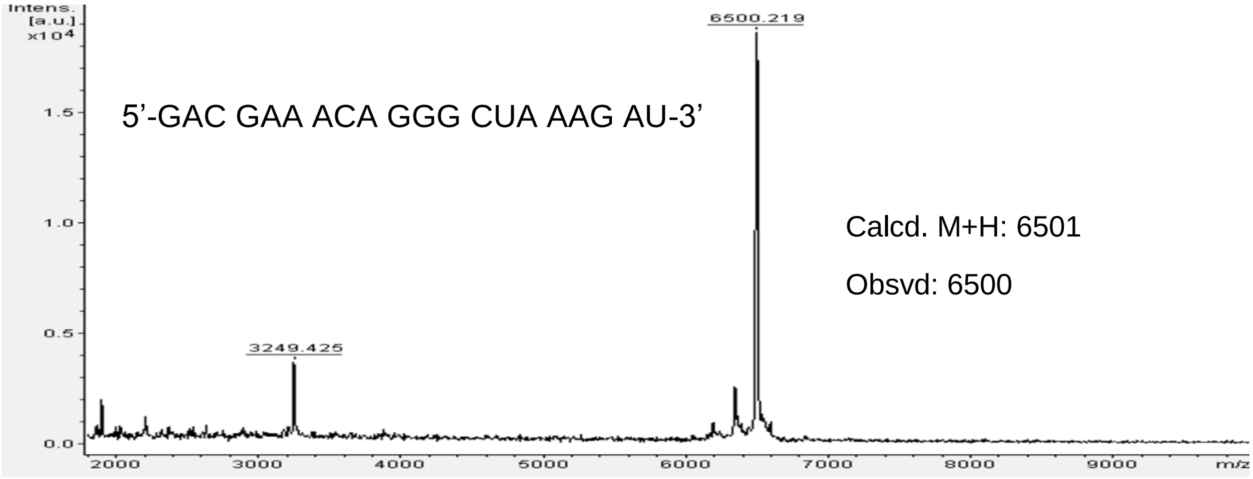

Figure S56. MALDI-TOF MS Spectrum of **9**

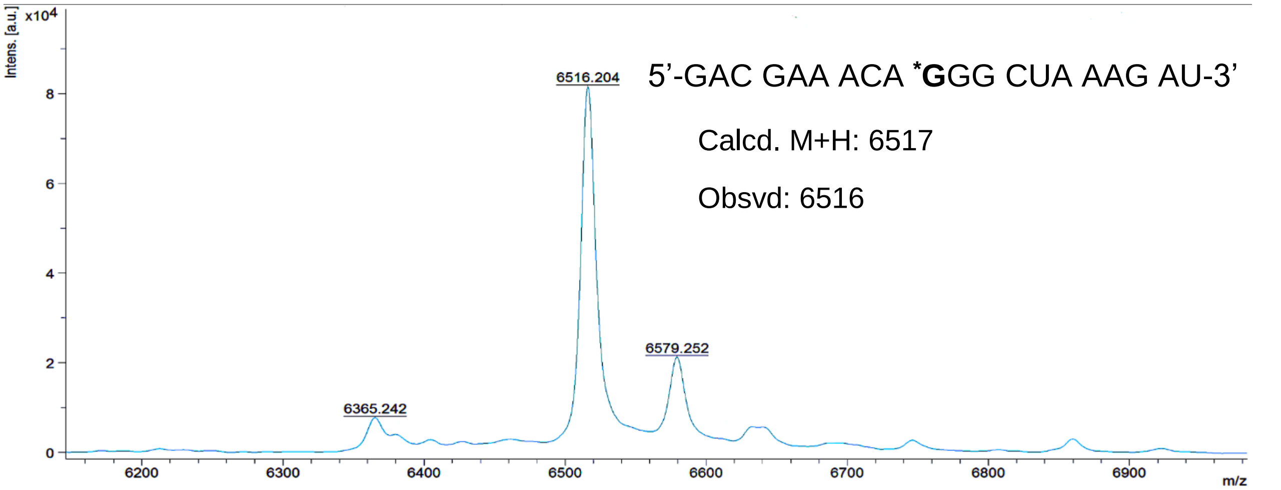

Figure S57. MALDI-TOF MS of **10**

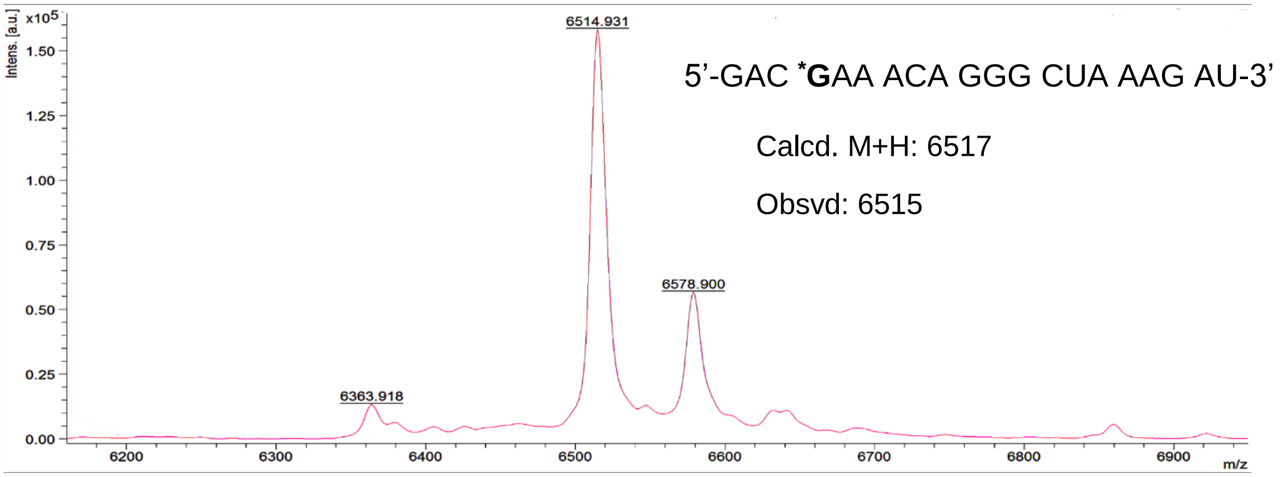

Figure S58. MALDI-TOF MS of **11**

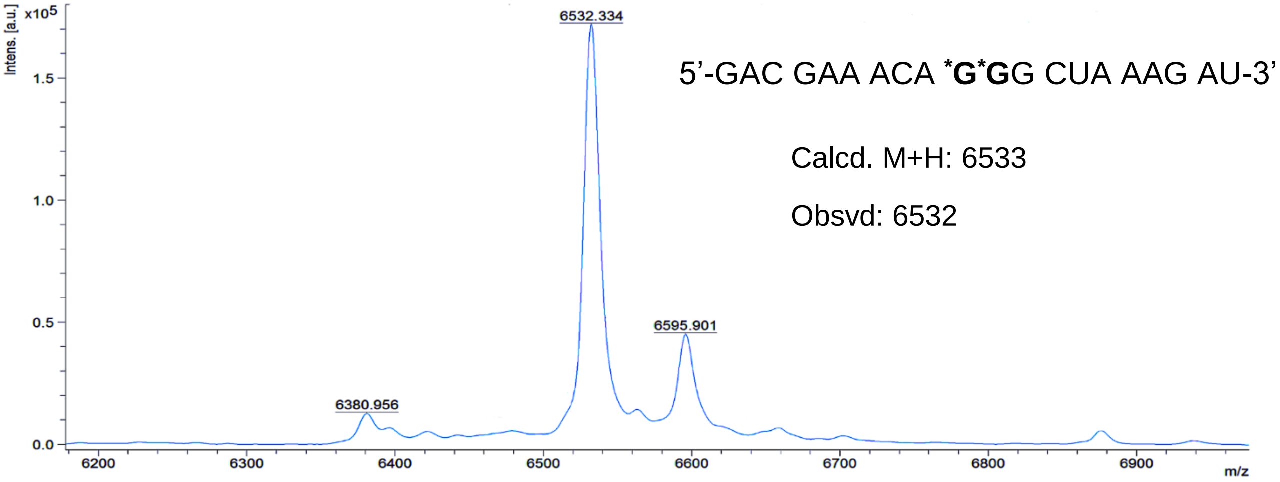

Figure S59. MALDI-TOF MS of **12**

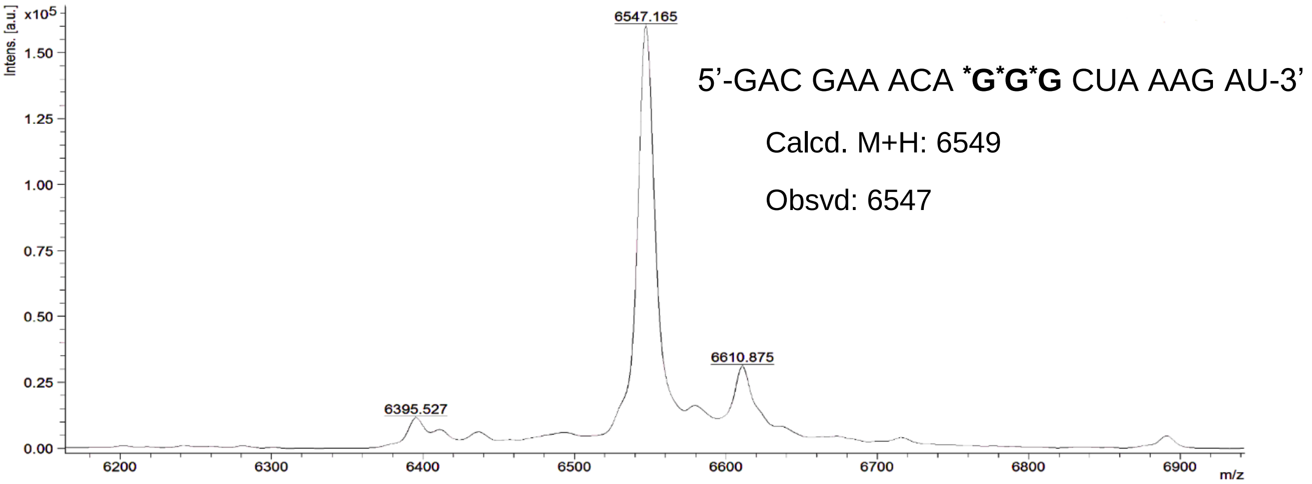

Figure S60. MALDI-TOF MS Spectrum of **13**

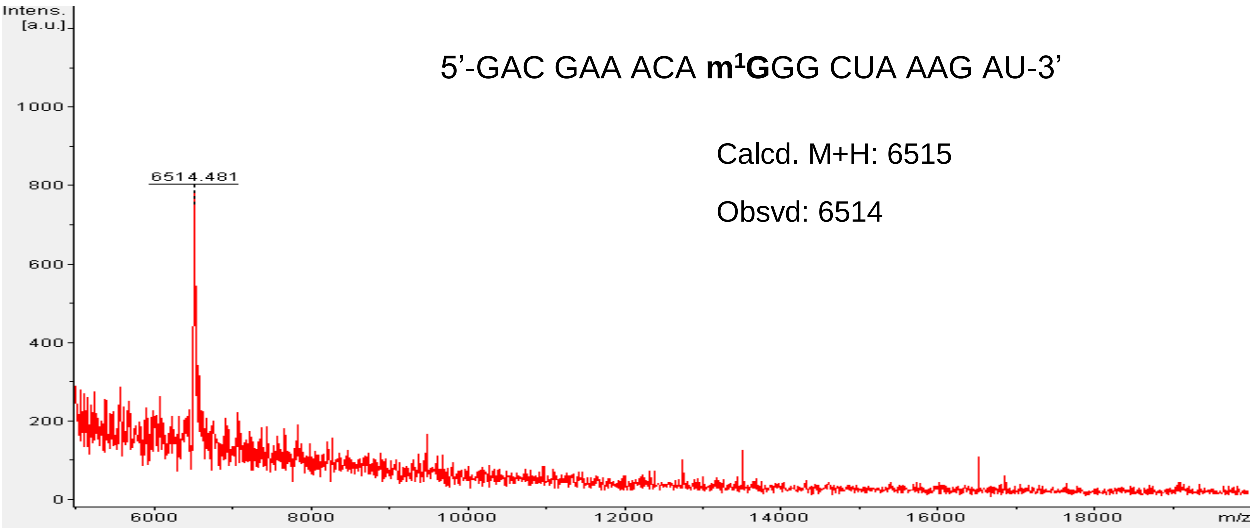

Figure S61. MALDI-TOF MS of **14**

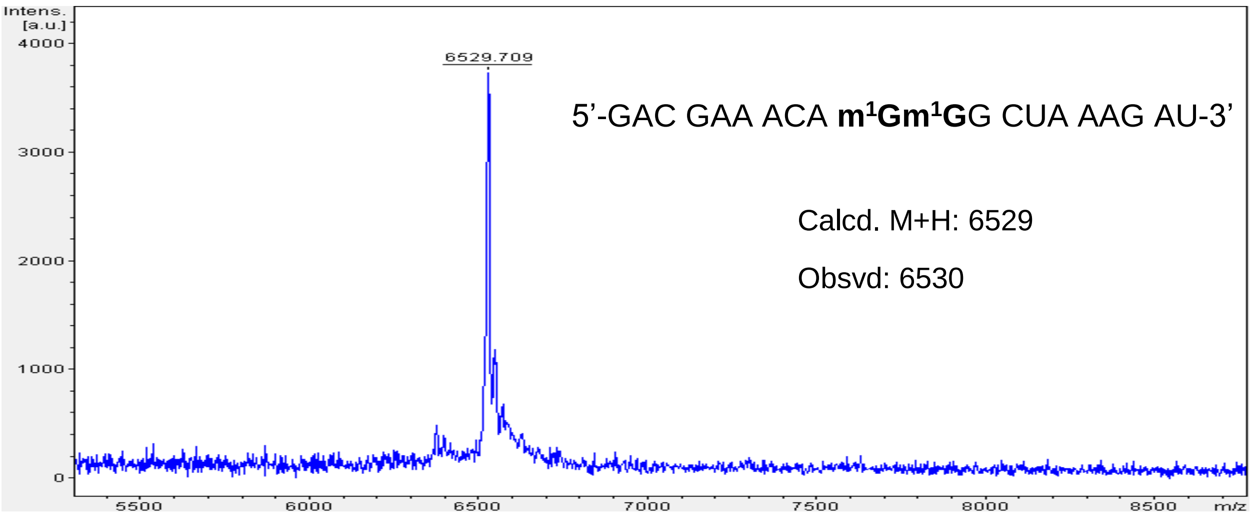

Figure S62. MALDI-TOF MS of **15**

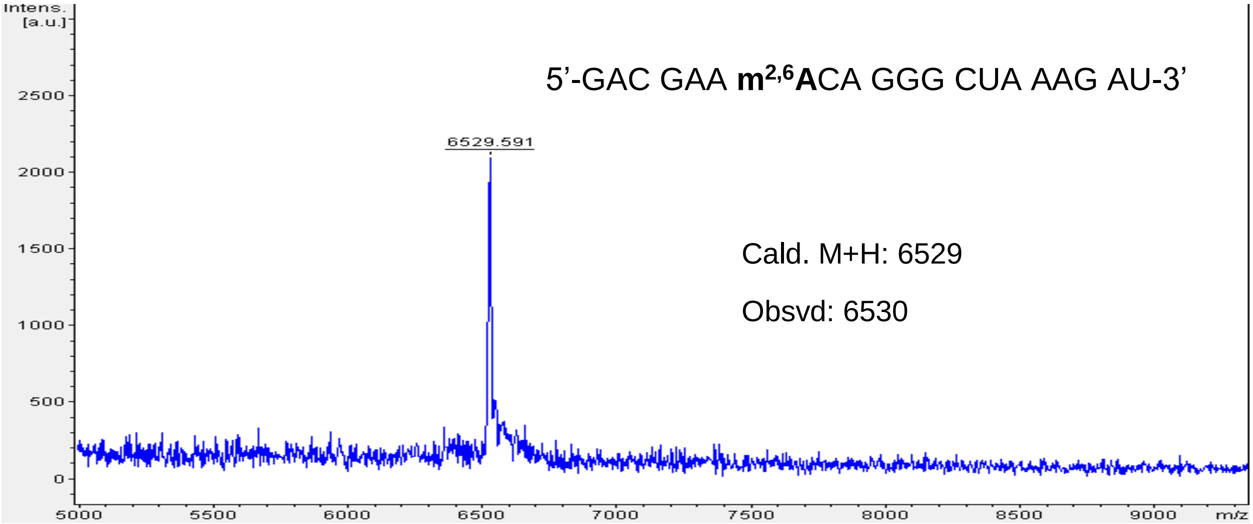

Figure S63. MALDI-TOF MS of **16**

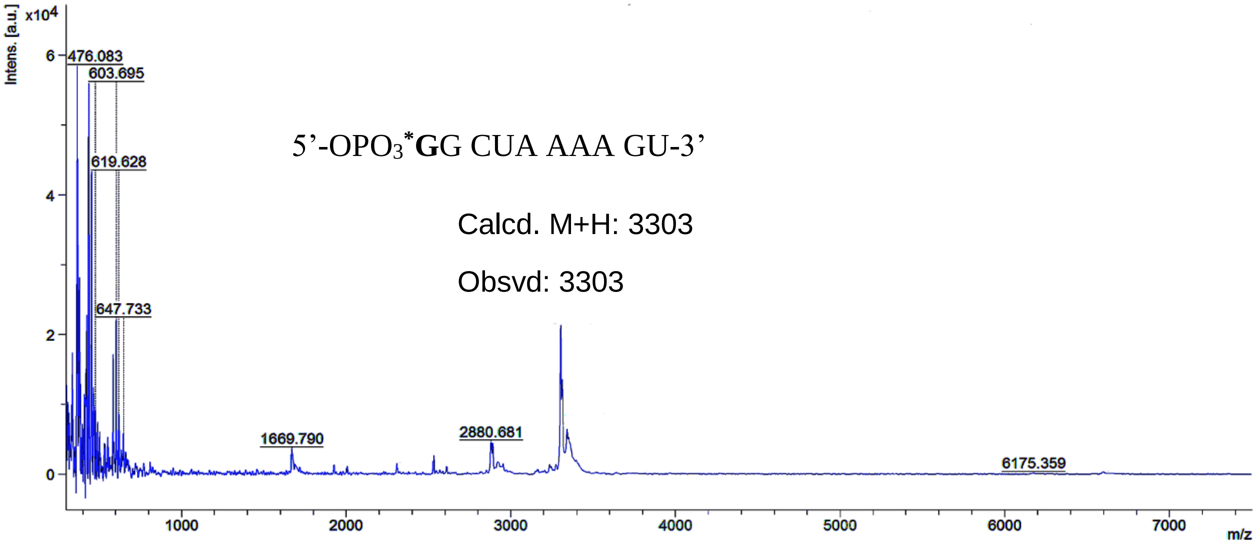

Figure S64. MALDI-TOF MS displaying the full spectra shown on Figure 1-D within the manuscript.

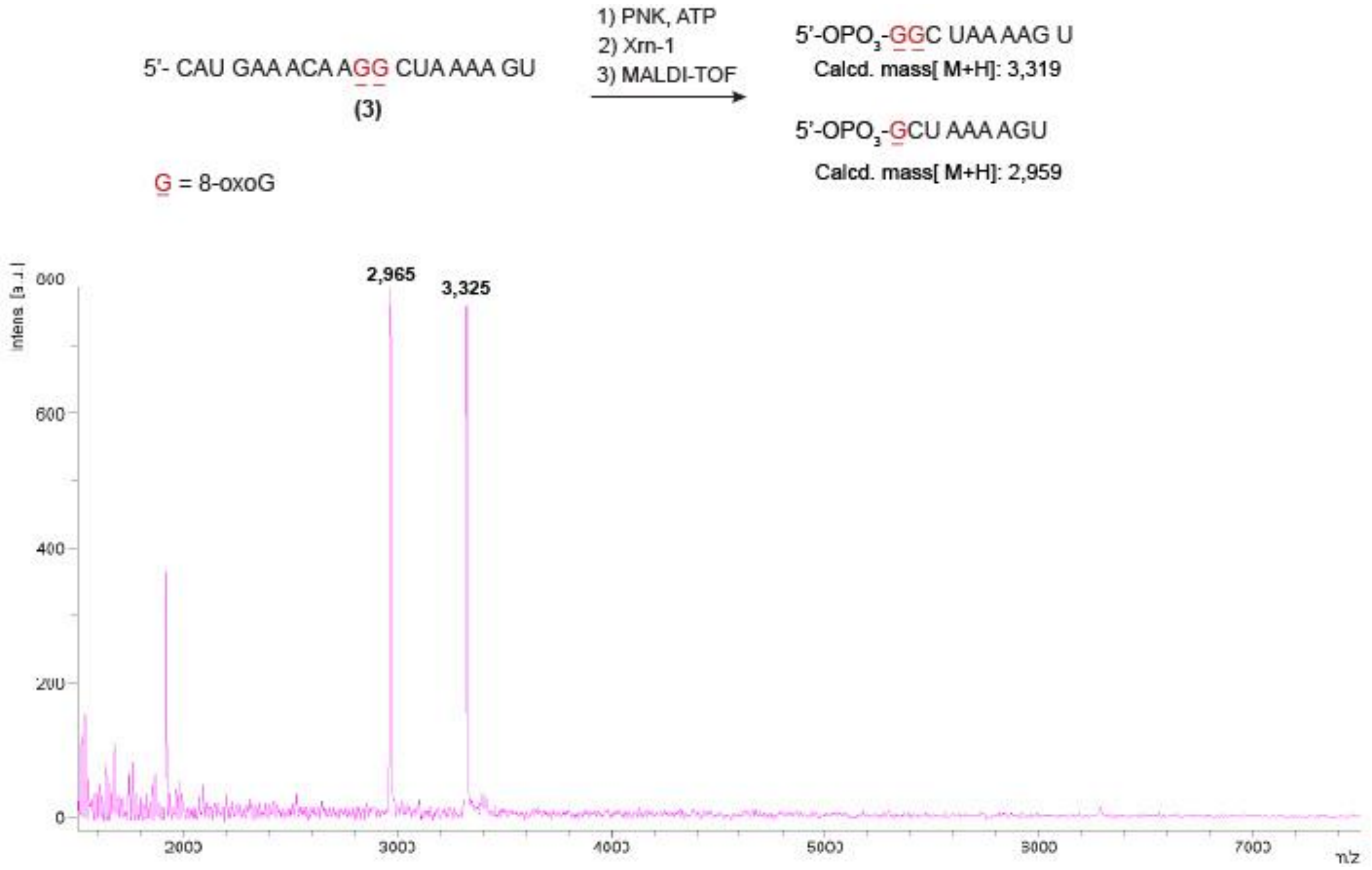

Figure S65. MALDI-TOF MS displaying the reaction between ON 3 and Xrn-1. The masses display a systematic error of M+6, which was often observed ins some experiments.

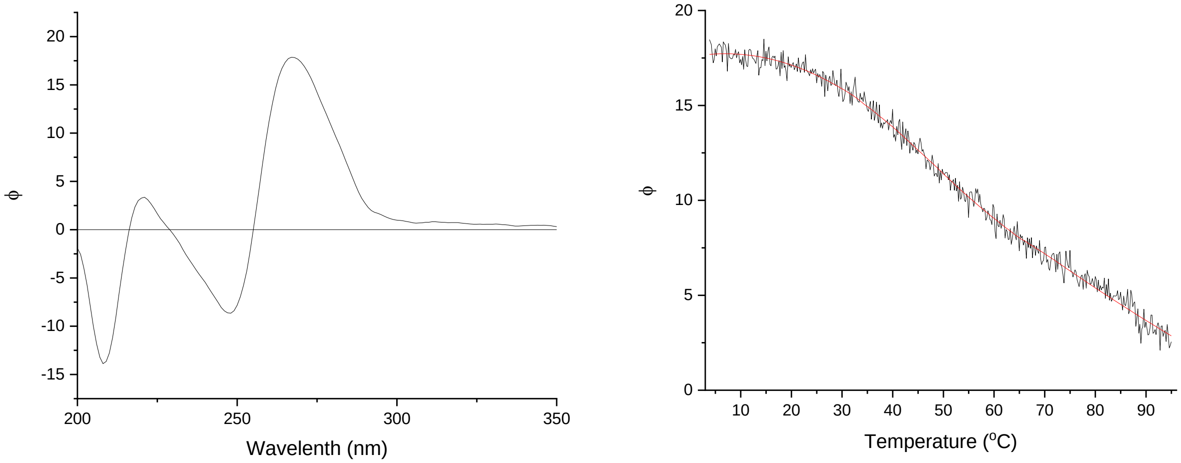

Figure S66. CD spectrum (left) and thermal denaturation measurement (T_m) (right) of ON **1**

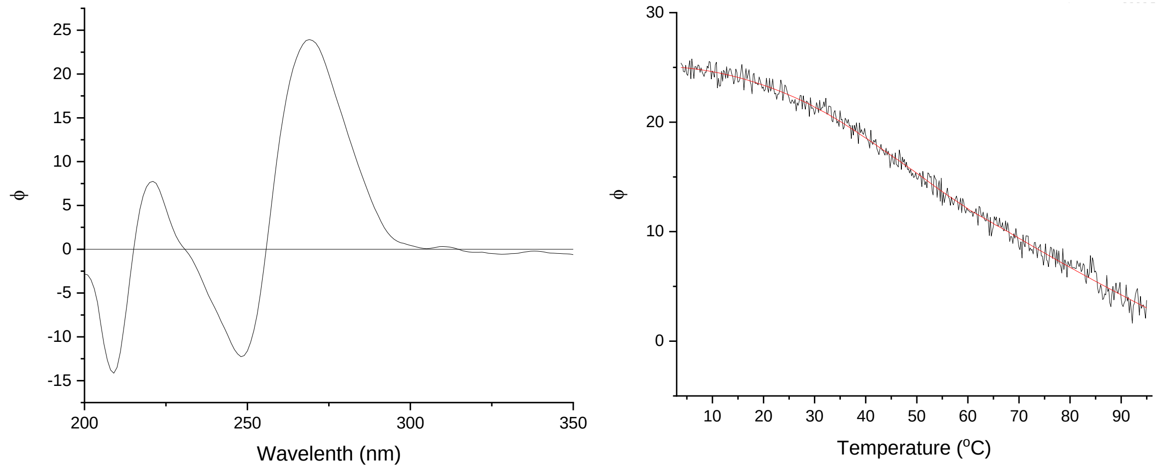

Figure S67. CD spectrum (left) and thermal denaturation measurement (T_m) (right) of ON **2**

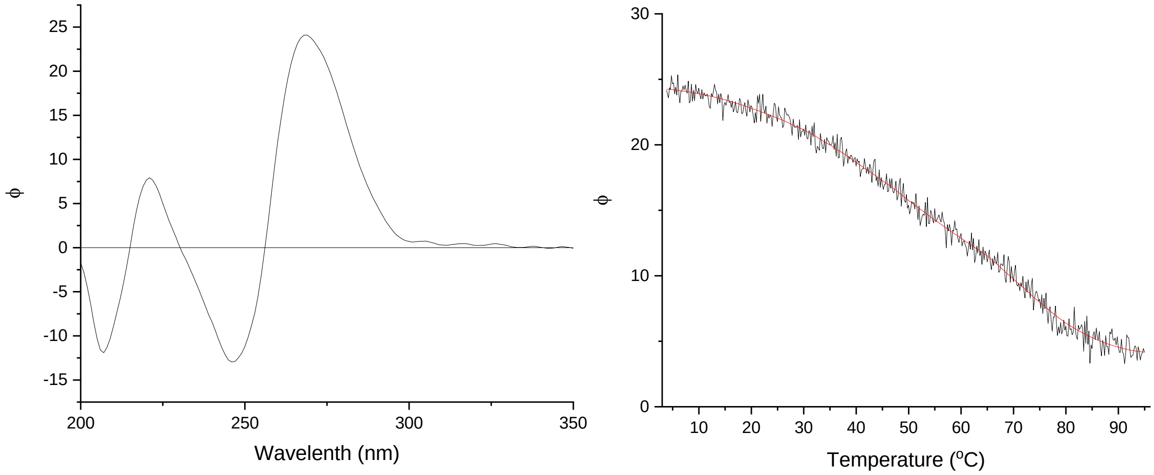

Figure S68. CD spectrum (left) and thermal denaturation measurement (T_m) (right) of ON **3**

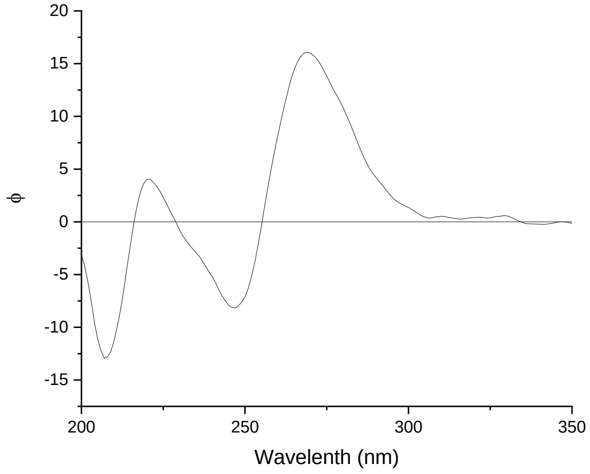

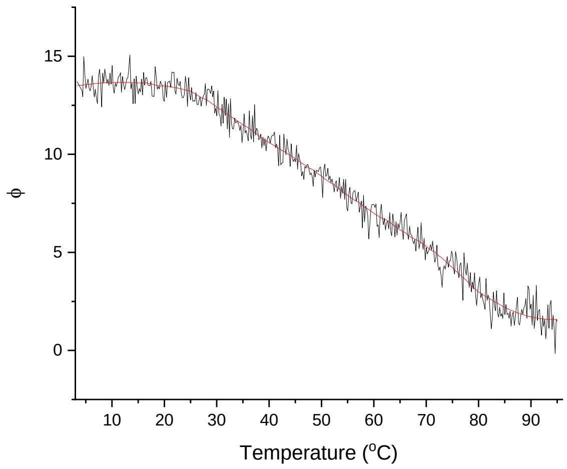

Figure S69. CD spectrum (left) and thermal denaturation measurement (T_m) (right) of ON **6**

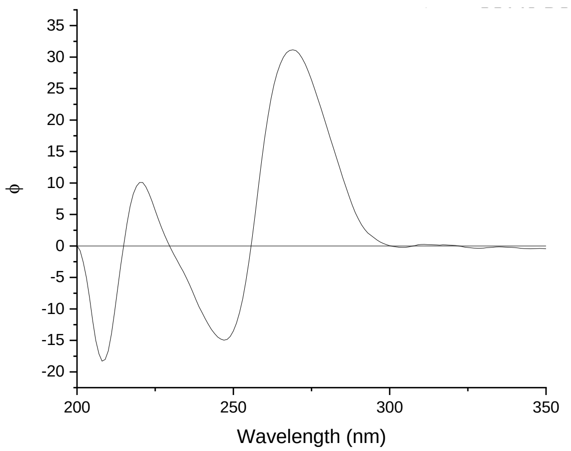

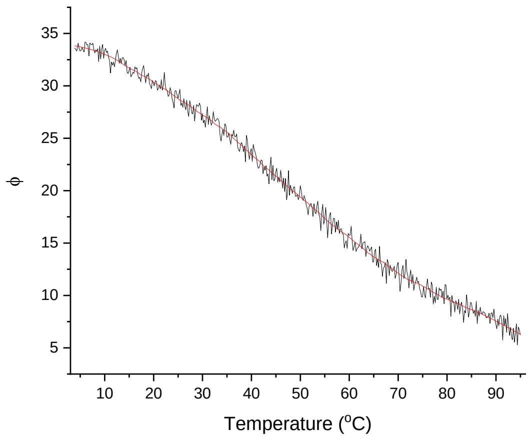

Figure S70. CD spectrum (left) and thermal denaturation measurement (T_m) (right) of ON **7**

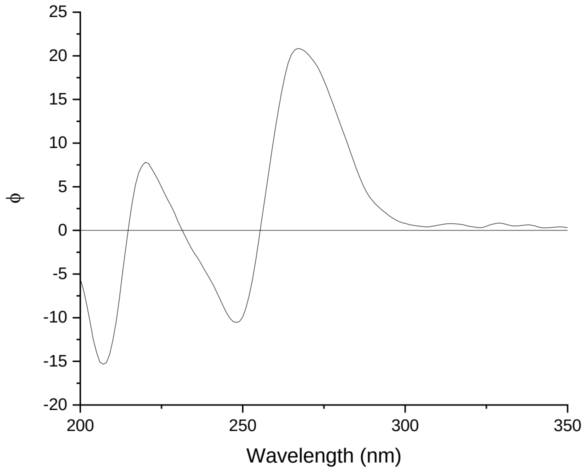

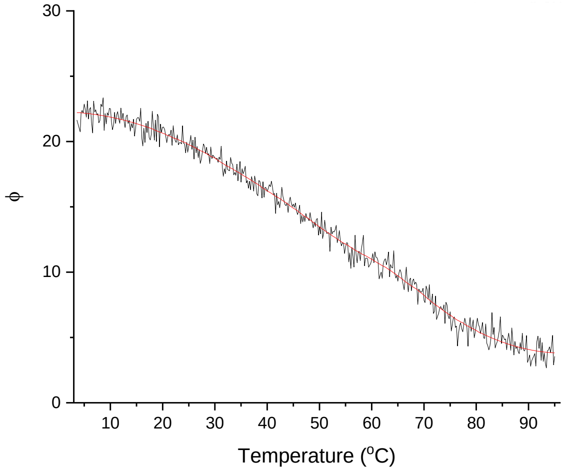

Figure S71. CD spectrum (left) and thermal denaturation measurement (T_m) (right) of sON **8**

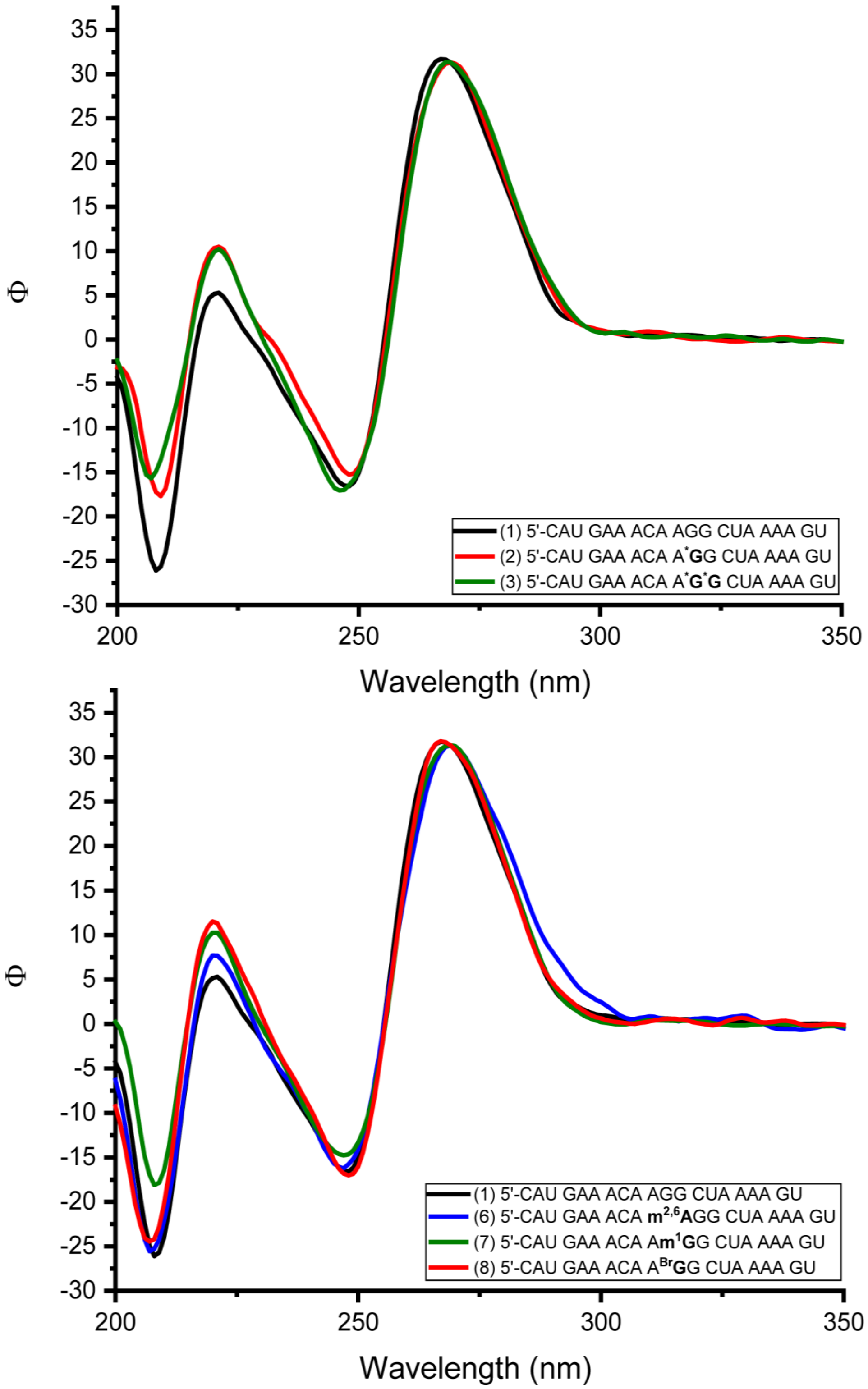

Figure S72. CD spectra overlay of ONs **1-3** (top) and **1**, **6-8**.

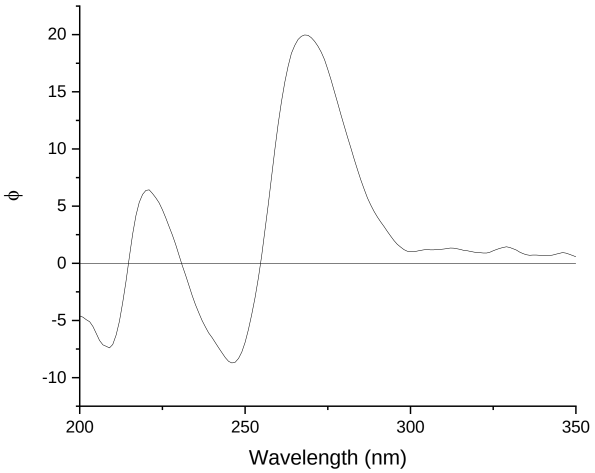

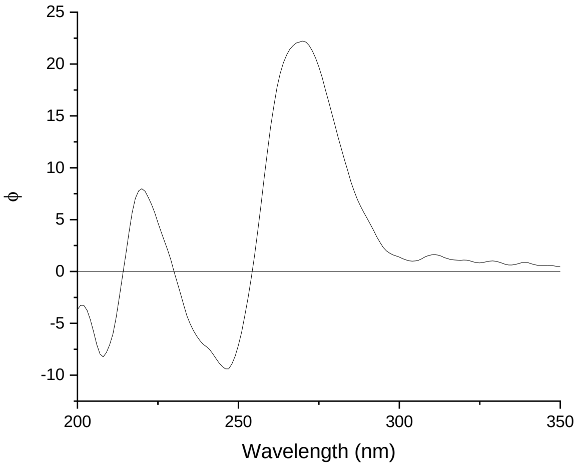

Figure S73. CD spectrum of **9** (left) and **10** (Right)

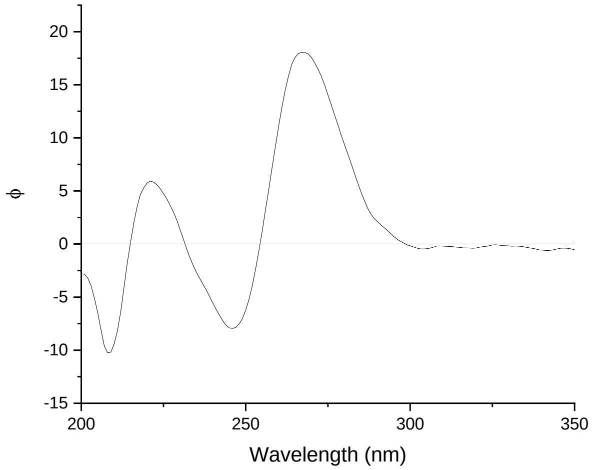

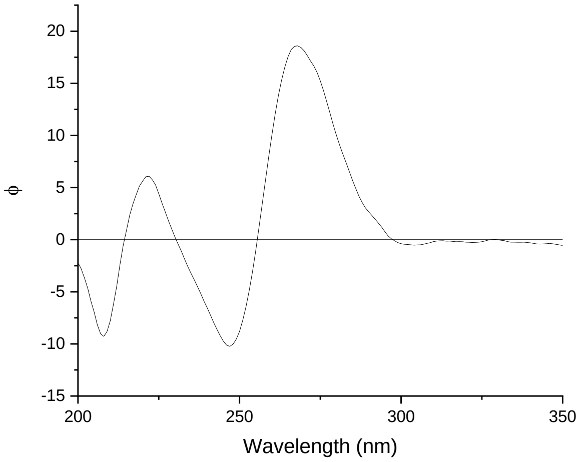

Figure S74. CD spectrum of **11** (left) and **12** (Right)

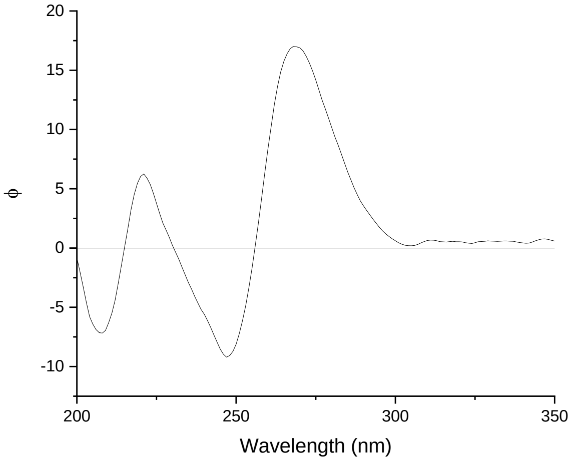

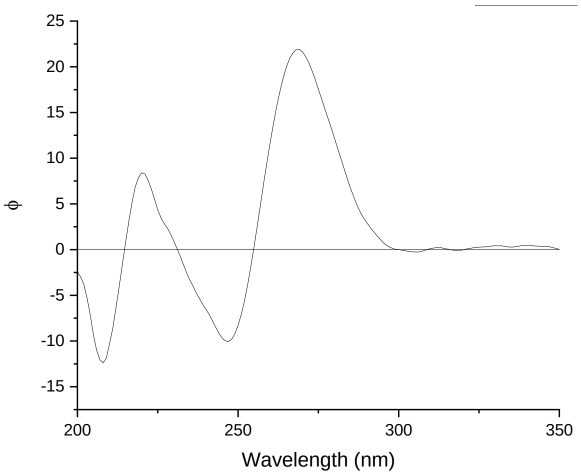

Figure S75. CD spectrum of **13** (left) and **14** (Right)

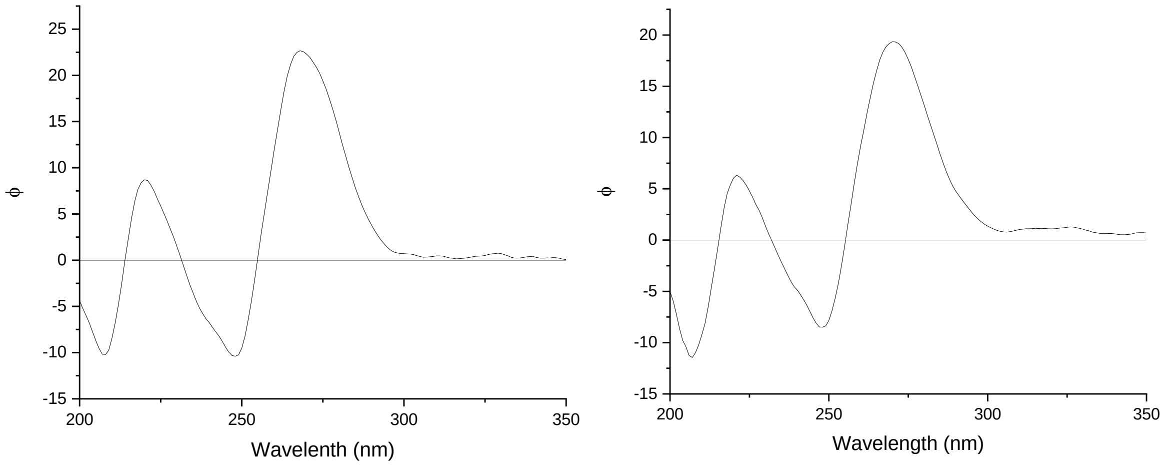

Figure S76. CD spectrum of **15** (left) and **16** (Right)

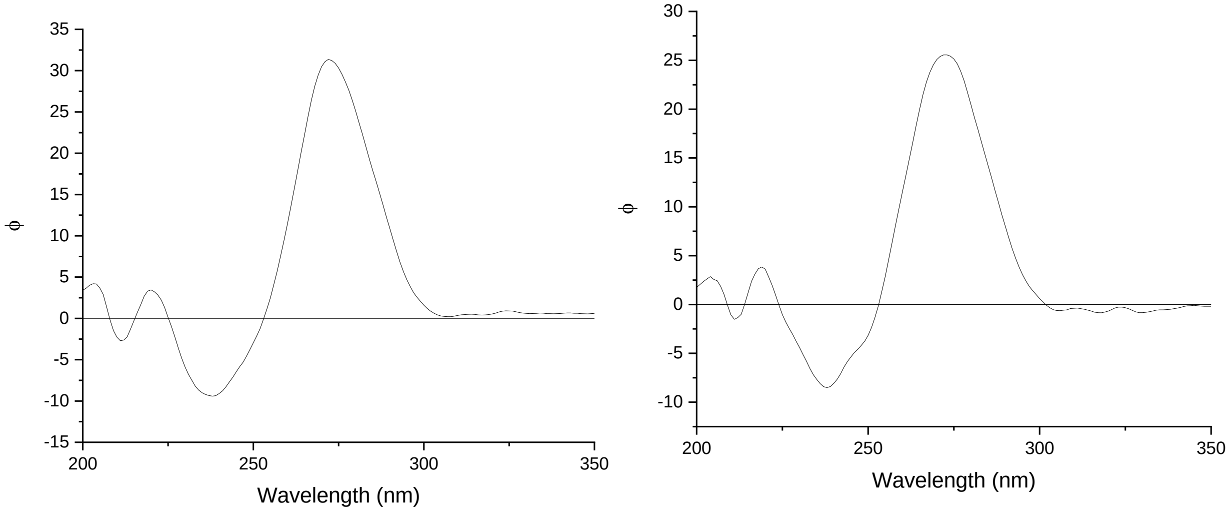

Figure S77. CD spectrum of **17** (left) and **18** (Right)

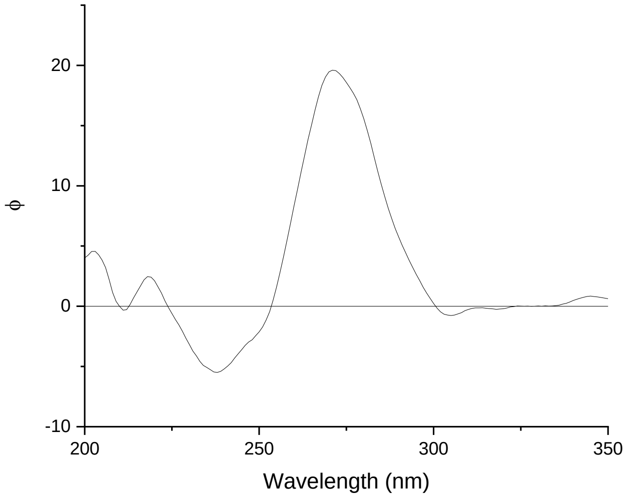

Figure S78. CD spectrum of **19**

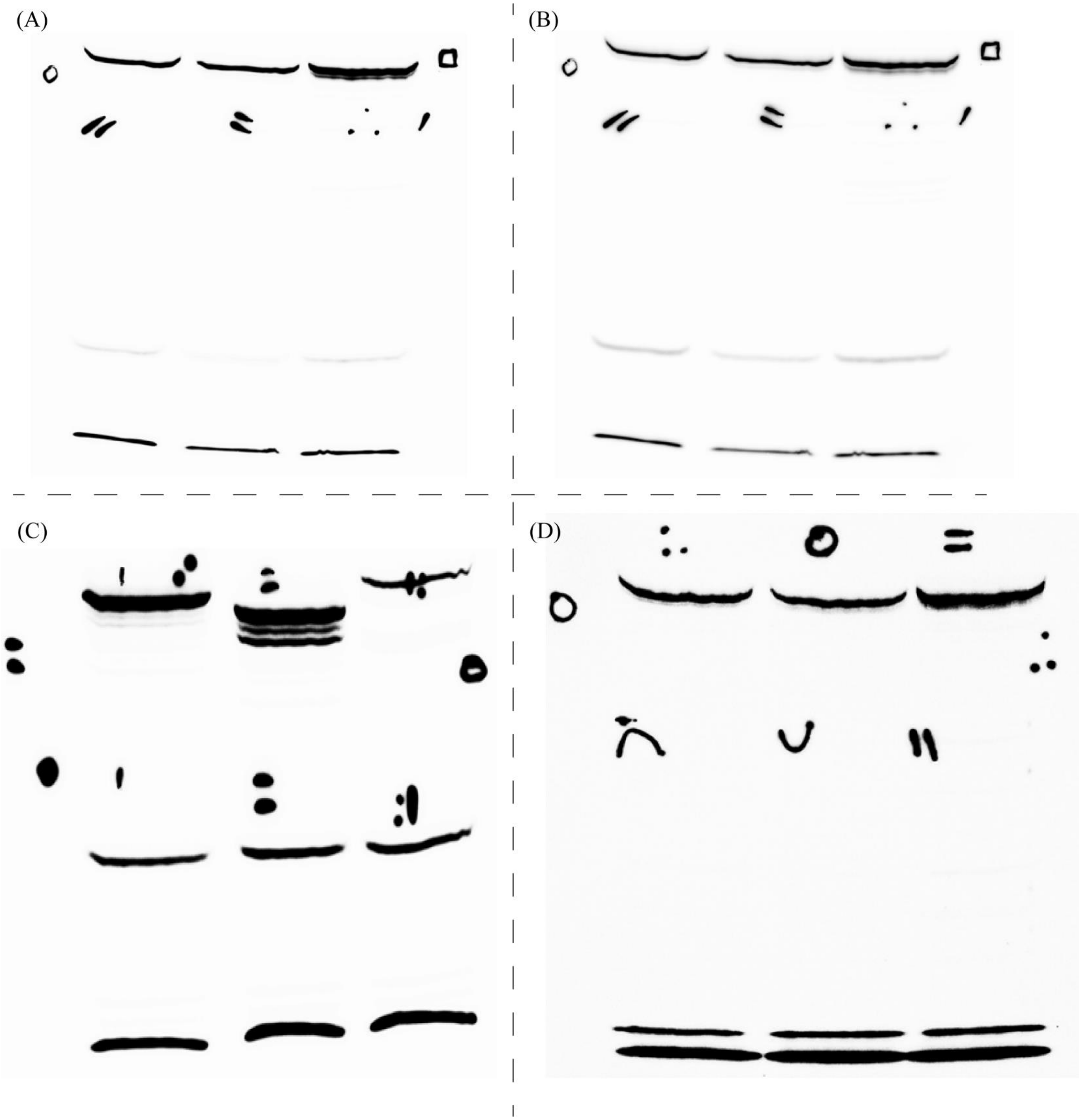

Figure S79. Typical experiments for the 3'-labeling experiments, where the top band was extruded and eluted as described in the experimental section. The examples above provide examples of purified RNAs carried out at different times, and on different gel plates (A: ONs 1-3; B: ONs 6-8; C: ONs 9-12; D: ONs 17-19). The various markings were used as guide to extrude the desired band.

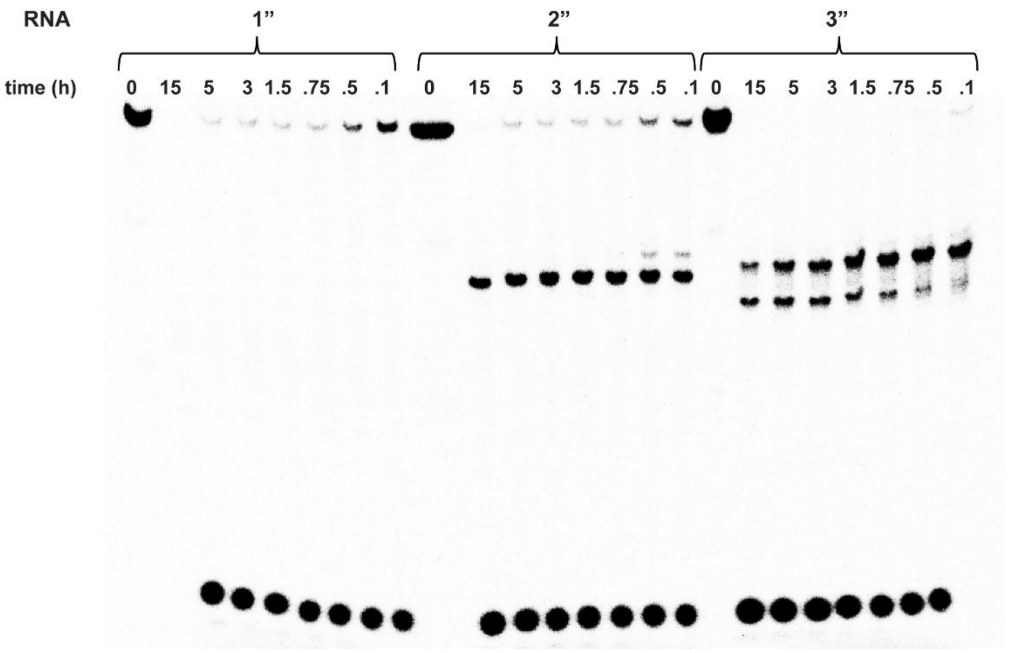

Figure S80. Time dependent experiment as a function of incubation time in the presence of Xrn-1, using RNAs **1”- 3”**.

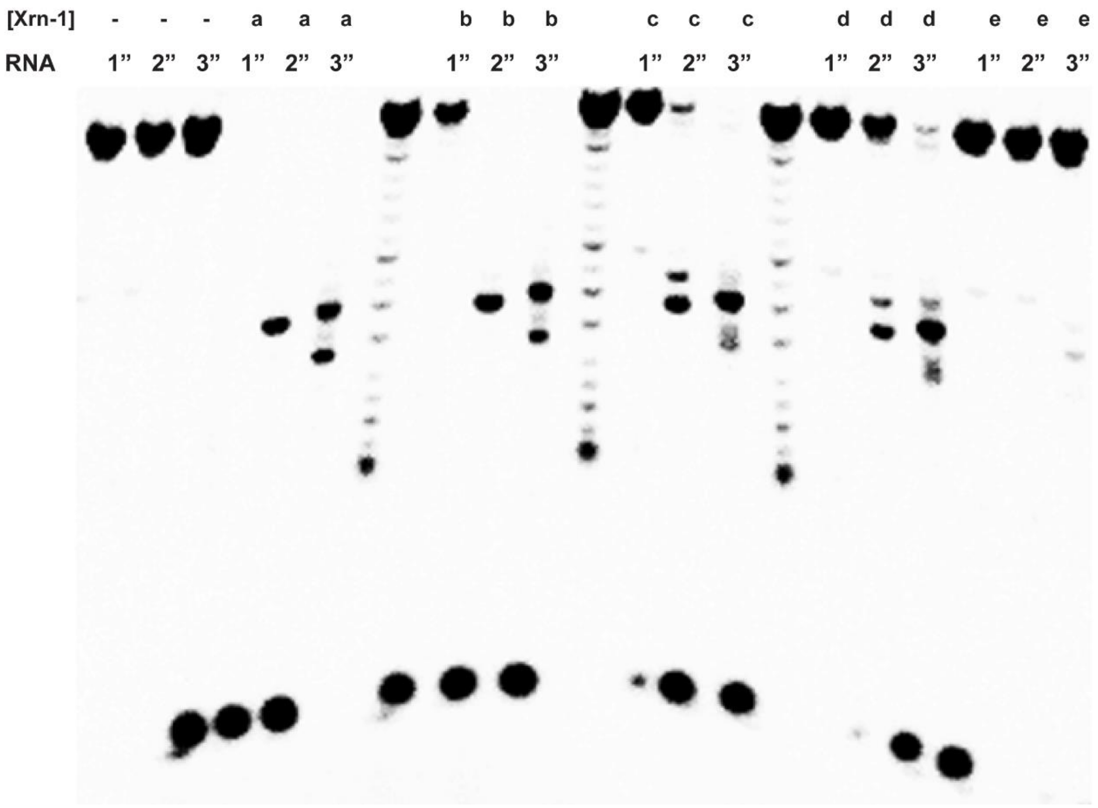

Figure S81. Experiment as a function of [Xrn-1], using RNAs **1”- 3”**. a= 8%; b = 4.5%; c = 1.6%; d = 0.8%; e = 0.1%.

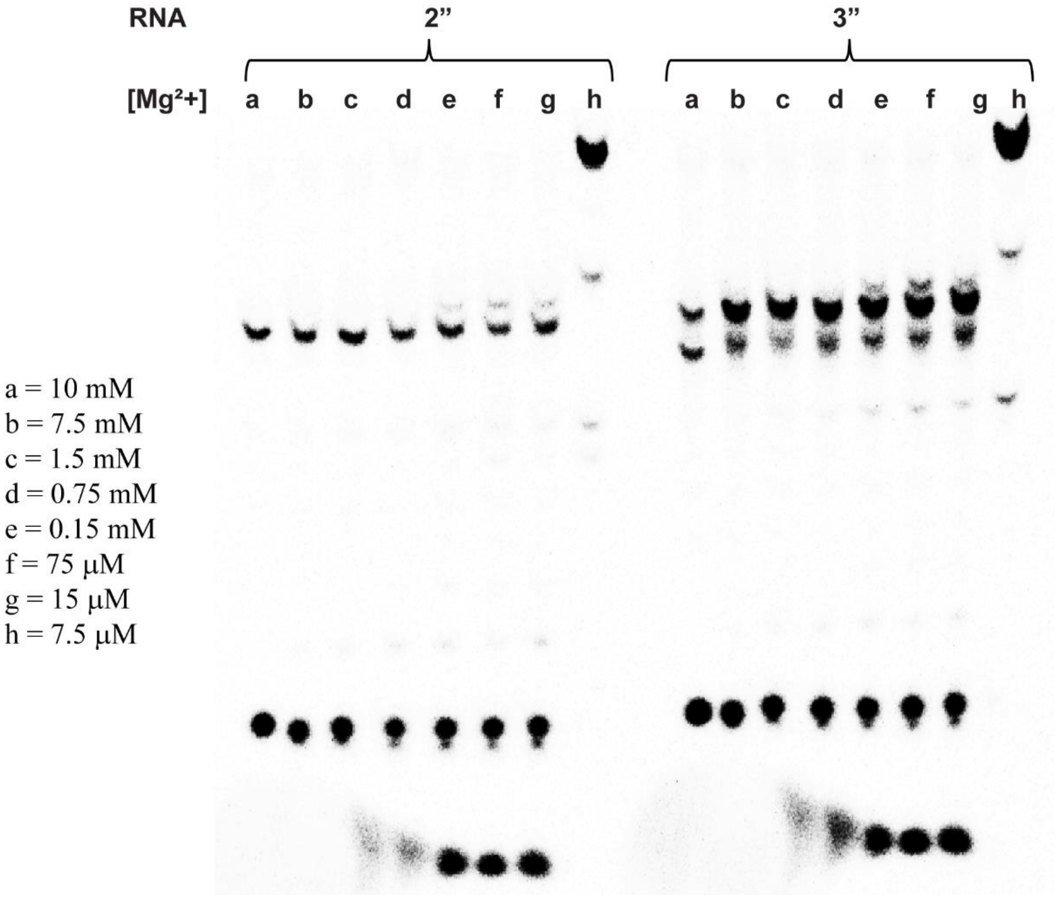

Figure S82. Experiment as a function of [Mg^{2+}], using RNAs **2”**& **3”**.

Predicted structures for ON **1”**

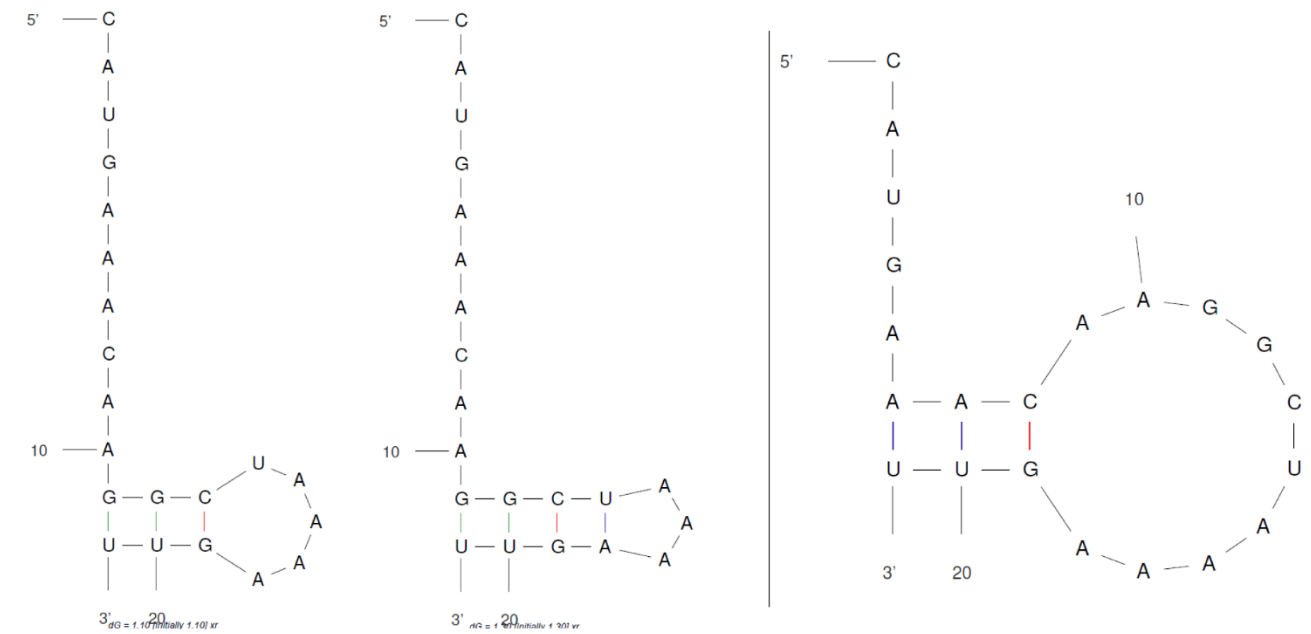

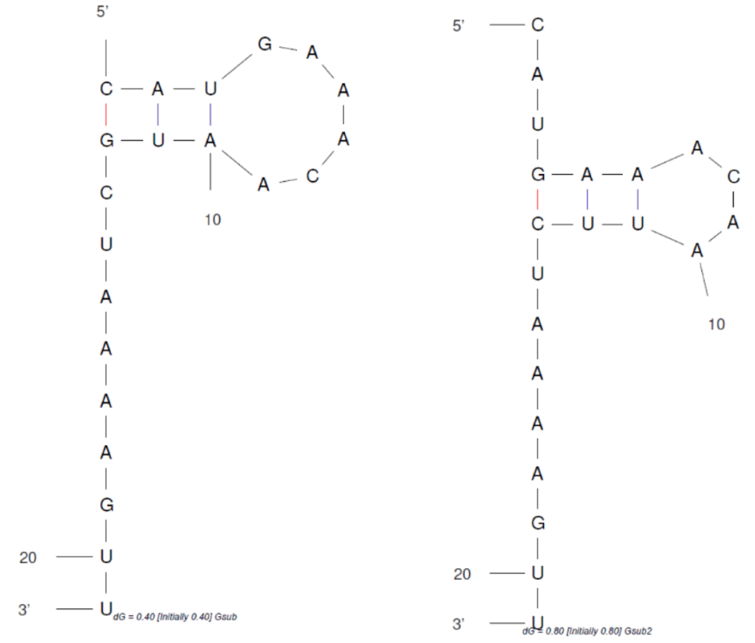

Predicted structures for ON **2”** (left) and **3”** (right), where 8-oxoG was substituted with a U.

Figure S83. Predicted folding, using UNAFold, of ONs **1” - 3”**.

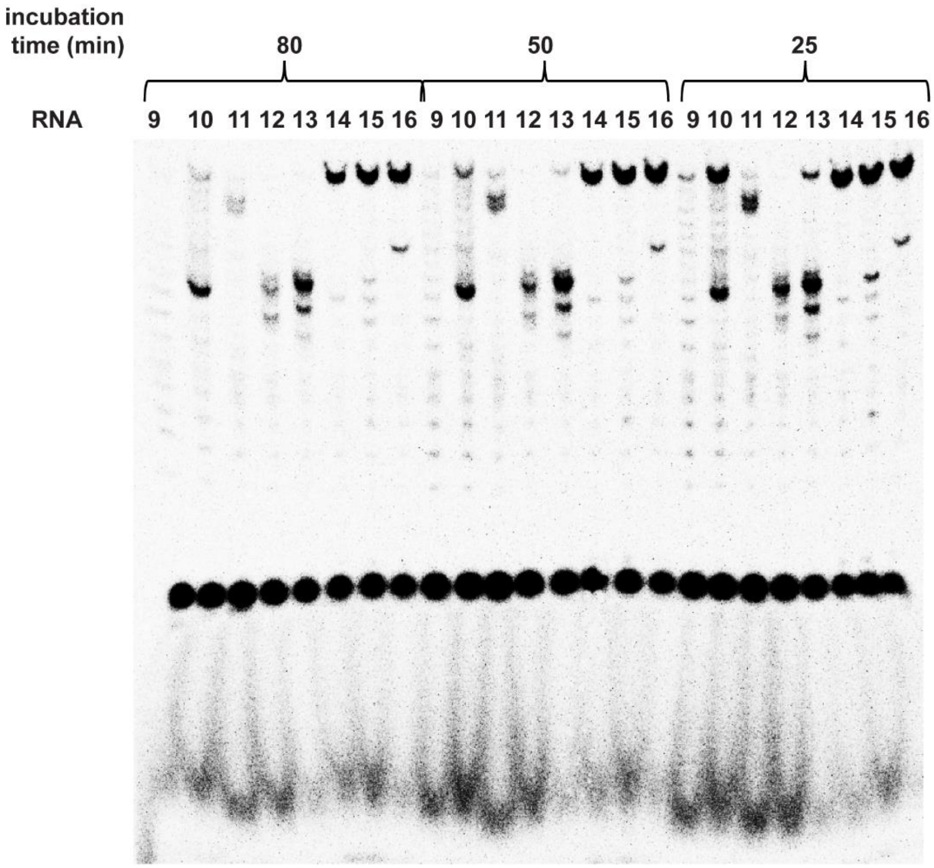

Figure S84. Treatment of ONs **9-16** with Xrn-1 at different time intervals.

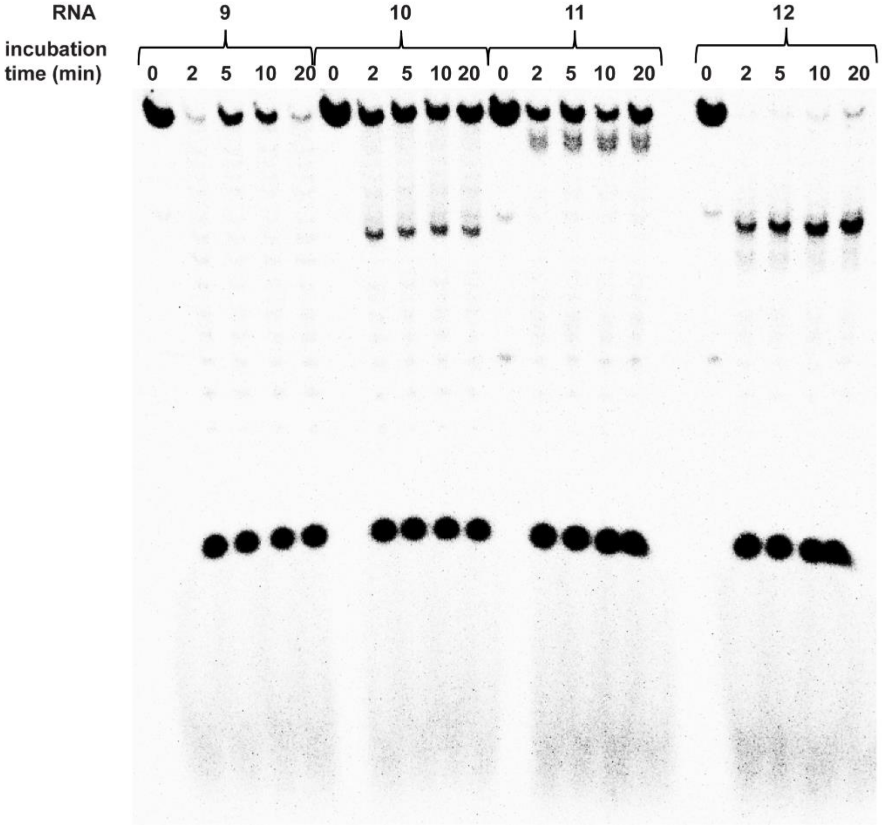

Figure S85. Treatment of ONs **9-12** with Xrn-1 at different time intervals.

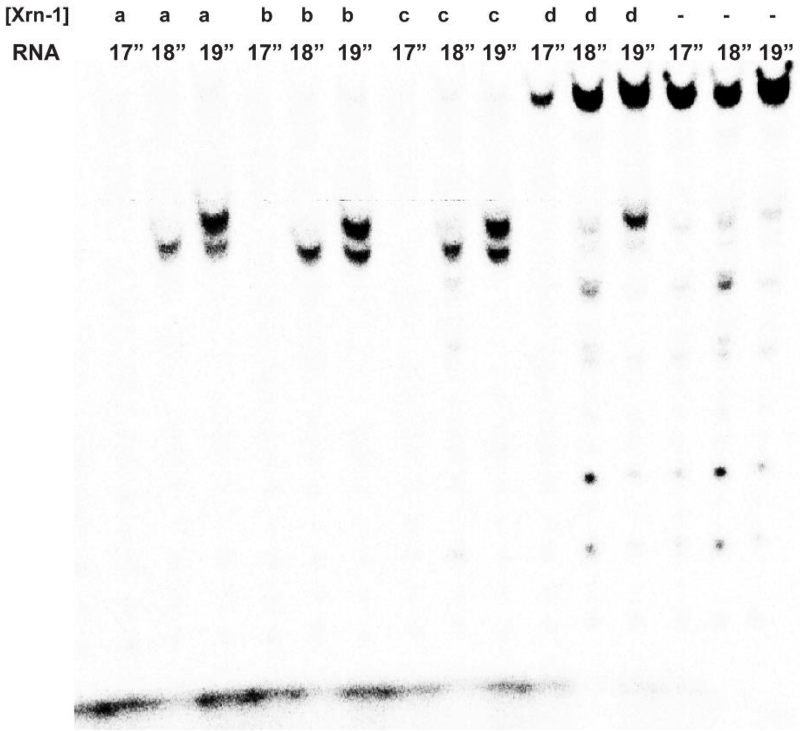

Figure S86. Treatment of ONs **17-19** at various [Xrn-1].

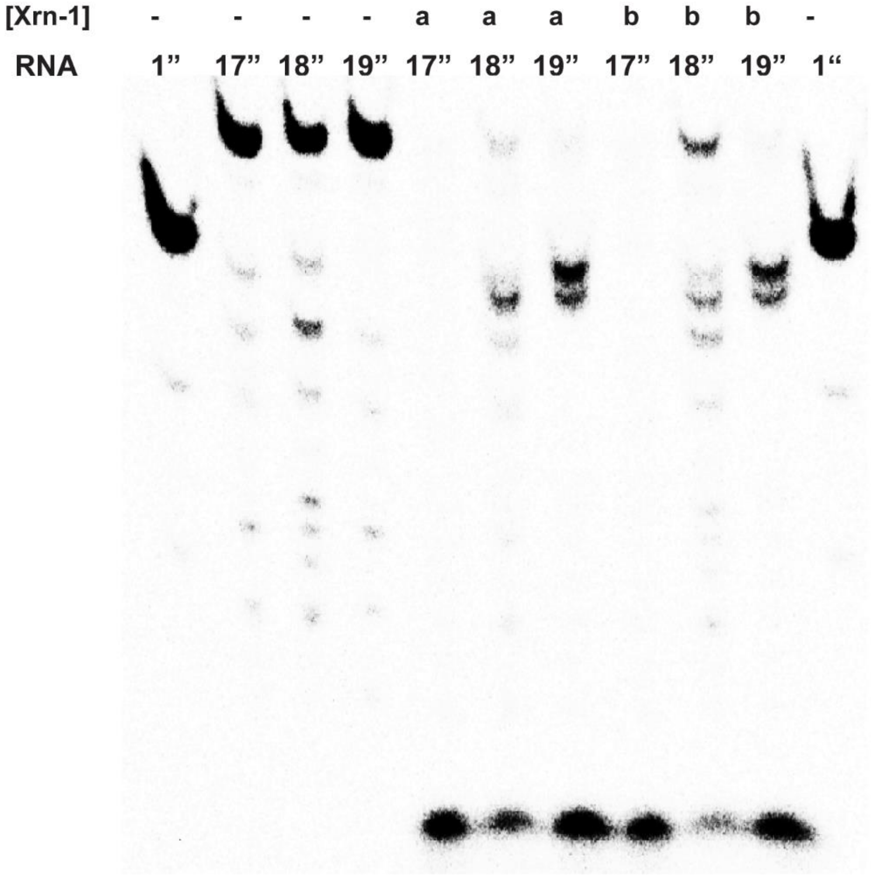

Figure S87. Treatment of ONs **17-19** at various [Xrn-1]. A marker was added that provided indication of the site of stalling.

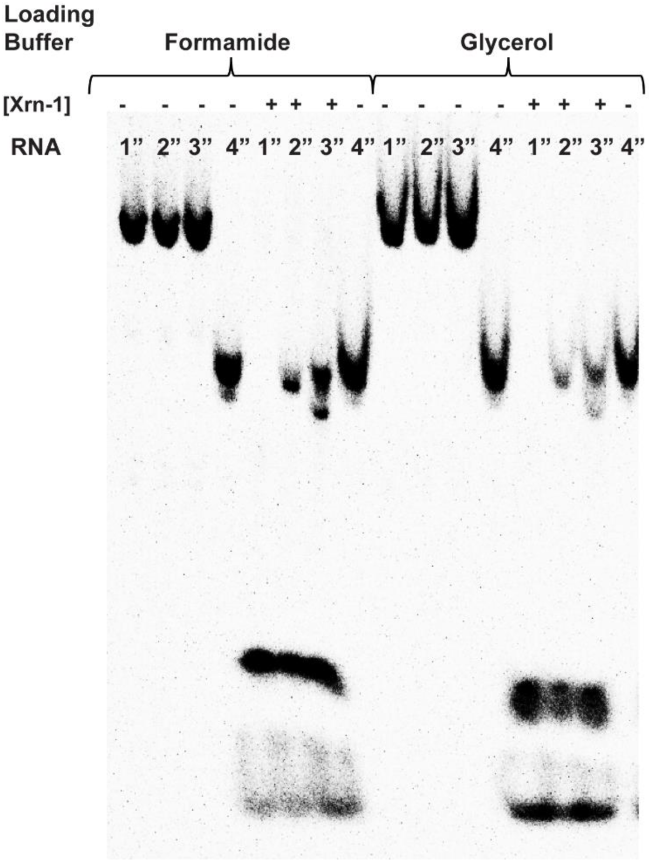

Figure S88. Treatment of ONs **1”-3”** with Xrn-1 using native PAGE. Fragment **4**” was added to track the expected formation of the corresponding fragment.

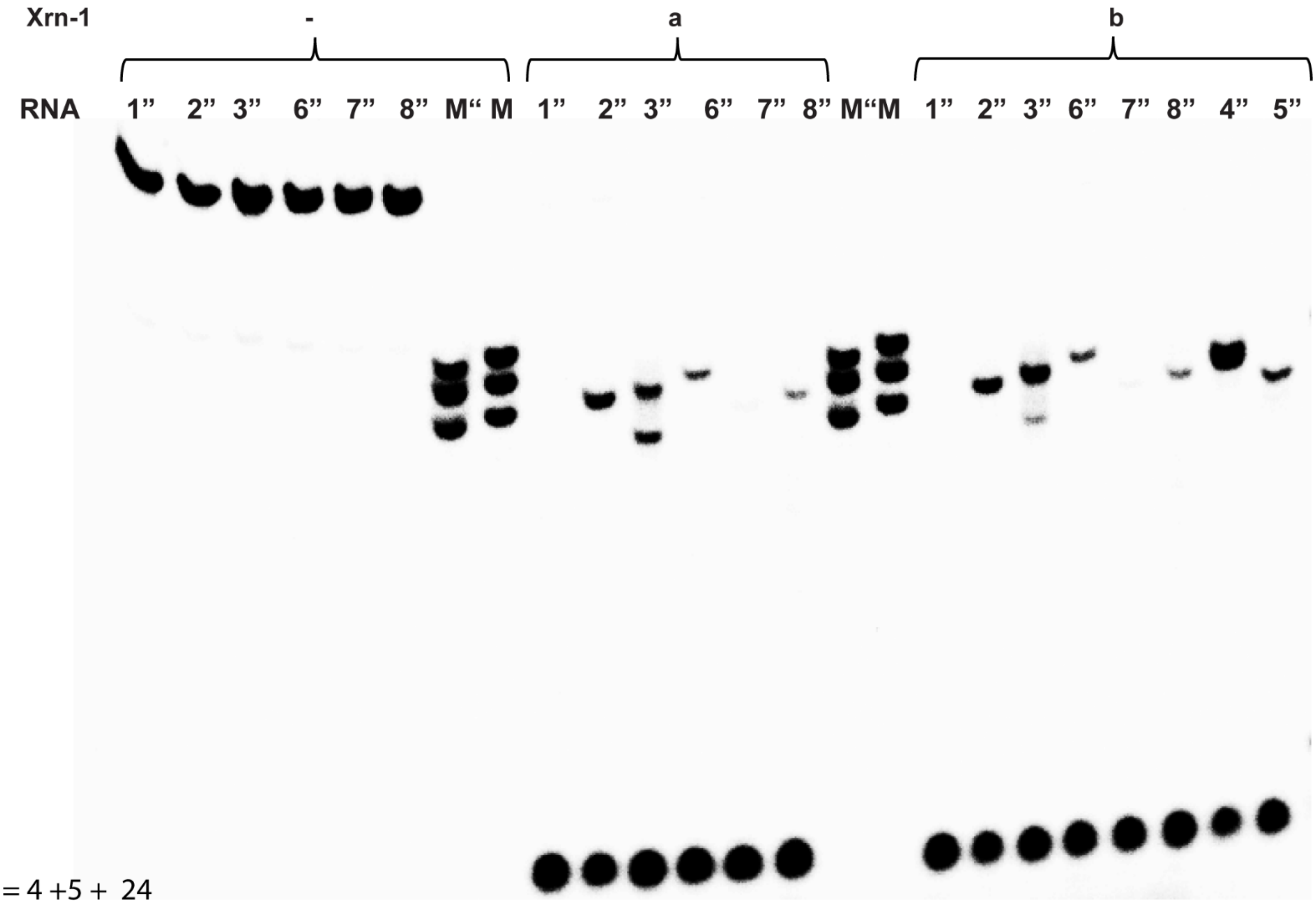

M = 4 +5 + 24

24 = 5′-GAC GAA ACA (8-oxoG)-UMeP

Figure S89. Treatment of ONs **1**”-**8”** with Xrn-1 using dPAGE. M” were 5’-phosphorylated, while M were not phosphorylated.

References

[1] Höbartner, C., Kreutz, C., Flecker, E. Ottenschläger, E. Pils, W. Grubmayr, K., Micura, R., *Monatsh. Chem.* **2003**, 134**,** 851–873.

[2] Sebastien Debarge, Jan Balzarini, & Anita R. Maguire, *J. Org. Chem.* **2011**, *76*, 105-126.

[3] Ostrand, R. V., Jacobsen, C., Delahunty, A., Stringer, C., Noorbehesht, R., Ahmed, H.; Awad, A. M. Nucleosides, Nucleotides Nucleic Acids **2017** 36..3, 181-197

[4] Korhonen H, Mikkola S, Williams NH. Chem. Eur. J. **2012**, 18, 659-670

[5] Kiggins, C., Skinner, A., & Resendiz, M. J. *ChemBioChem,* **2020** 21, 1347-1355.

[6] Glennon, M. M., Skinner, A., Krutsinger, M., Resendiz, M. J., *PLoS ONE* **2020** 15, e02350102
